# Integrated optimization modelling framework for low-carbon and green regional transitions through resource-based industrial symbiosis

**DOI:** 10.1038/s41467-024-48249-6

**Published:** 2024-05-07

**Authors:** Xin Xie, Hang Fu, Qisheng Zhu, Shanying Hu

**Affiliations:** 1 Center for Industrial Ecology, Department of Chemical Engineering, Tsinghua University, Beijing, 100084 China; 2 Watershed Carbon Neutrality Institute, School of Resources & Environment, Nanchang University, Nanchang, 330031 China

**Keywords:** CD4-positive T cells, Sustainability

## Abstract

The development and utilization of bulk resources provide the basic material needs for industrial systems. However, most current resource utilization patterns are unsustainable, with low efficiencies and high carbon emissions. Here, we report a quantitative tool for resource-based industries to facilitate sustainable and low-carbon transitions within the regional economy. To evaluate the effectiveness of this tool, the saline Qinghai Lake region was chosen as a case study. After optimizing the industrial structure, the benefits of economic output, resource efficiency, energy consumption, solid waste reduction, and carbon emission reduction can be obtained. The scenario analyses exhibit disparities in different transition paths, where the carbon mitigation, economic output, and resource efficiency that benefit from optimal development paths are significantly better than those of the traditional path, indicating the urgency of adopting cleaner technology and industrial symbiosis for regional industries.

## Abstract (web summary)

This study reports a quantitative tool for resource-based industries aimed at supporting sustainable, low-carbon regional transitions. The results provide optimal transition paths over traditional methods, highlighting the need for cleaner technology and industrial symbiosis.

## Introduction

The development and utilization of bulk resources, including oil, natural gas, mines, and saline lake brine, meet the basic raw material requirements for industrial systems. Industrialization, urbanization, and the increasing demand for high-value-added industrial products have contributed to the formation of complex resource use patterns for the regional economy. However, the majority of current resource use systems exhibit unsustainable patterns, leading to high greenhouse gas emissions, low resource use efficiency, excessive environmental impacts, and a low input‒output ratio, which promote industrial sustainability^1,2^.

The complex multidimensional characteristics of resource endowments are vital for developing a unique industry structure for the regional economy. Thus, high-quality and sustainable resource use requires systematic planning and optimization measures to determine the optimal path for regional industrial development. The concept of industrial ecology is a multifield discipline that includes system engineering, mathematical programming, and economics to construct a sustainable and stable industrial development pattern^1,3–5^. Industrial symbiosis (IS), which originates from the concept of an eco-industry, is an effective tool for integrating resource endowment and industrial technologies to maximize the benefits of regional resource use^1,2,6–8^.

The concept of IS has been extensively utilized to evaluate the sustainability and carbon emissions of resource use patterns at a regional scale^9^. Currently, the scientific efforts of IS focus mainly on the following routes: qualitative, quantitative, and systematic planning. The qualitative route usually evaluates the formation and construction of an ecological industrial system via self-organizing/self-emerging/serendipitous and planned/designed/goal-directed methods by constructing multidimensional indicators and full-range input‒output process matching^7,8,10–12^, which is extensively used to evaluate IS formation at certain industrial parks, such as those in Kalundborg and Ulsan^7,13^. However, this route is based mainly on experience and expert planning under the current foundation of local industry, and mathematical and universal methods that can be used for planning in other regions are lacking, particularly when addressing multiple resources, products, and technologies^14,15^. Quantitative planning usually involves two or more resource optimizations, such as water^16,17^, energy, carbon, materials^18,19^, and a combination of these methods via energy and mass balance approaches^20–22^. Although analyzing the metabolism of key sources can provide valid information on energy, element, and resource use efficiency, it is still difficult to simultaneously cover and optimize the multiplicity of resource categories and account for their interactions^23–26^. Systematic planning is based on the construction of IS networks (ISNs), and some studies have evaluated the industrial symbiosis system of parks through social network and ecological network approaches using indicators such as resilience and centrality^27–29^. These routes may provide a better understanding of the systematic depiction of the industrial pattern of resource exploration; however, due to the complexity of the network, constructing complete and reliable ISNs from natural resources to products is difficult with the factors of resource endowment, existing industrial conditions, and relevant policies^14,15^. Thus, current IS studies still lack a systematic, quantitative, and general framework for designing a sustainable path transition of resource use patterns for the regional economy.

In this work, we present a resource-based regional industrial economy development optimization model (RRIEDOM). This is a bottom-up model that integrates a multimethodology of industrial ecology in material flow analysis, network analysis, and optimization techniques to address diverse economic, environmental, and resource objectives. The Qinghai Salt Lake Industrial Region is selected as a case study for this model. First, this model incorporates a comprehensive technology database comprising 358 chemical technologies and more than 200 chemical products to establish a complex industrial network, as described in detail in the Methods section. Second, utilizing this database, the current situation of the Qinghai Salt Lake Industry in 2020 and an optimal industrial structure that minimizes resource and environmental costs while maximizing economic output are evaluated. Third, to determine the most efficient pathway for transitioning from the existing industrial framework to the optimal structure with the lowest transition expenses, six scenarios are designed. These scenarios align with increasingly stringent climate policies by incorporating carbon-negative measures such as clean electricity^30,31^, carbon capture, utilization, and storage (CCUS)^32,33^, and steam boiler retrofitting^34,35^.

## Results

### Current status of the regional chemical industry

**Fig. 1:**
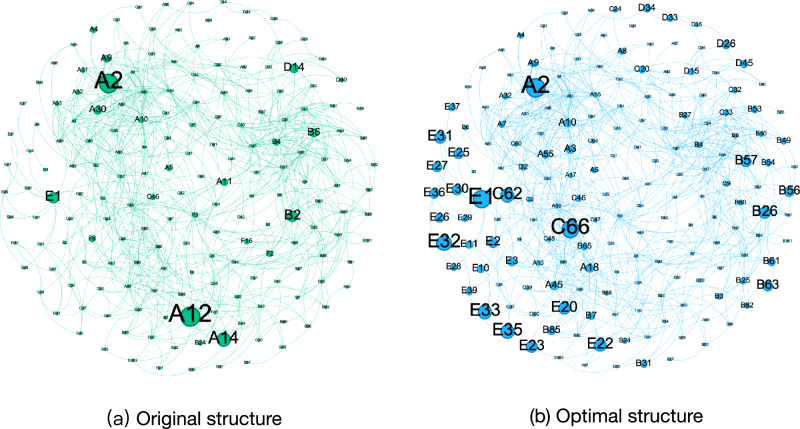
Original and optimal saline lake industrial structure. The original structure (**a**) is obtained by accounting for the industries in the Salt Lake Industrial Zone, and the optimal structure (**b**) of the Salt Lake Regional Industry is obtained after optimization under the guidance of the RRIEDOM. The industrial output value and diversity under the optimal industrial structure are greater than those under the original structure. The nodes represent specific chemical industry technologies, the size represents the industrial output value of each node, and the edges represent the corresponding material interactions. Each node is assigned a unique number, which is used as follows: A - saline lake industry (38 nodes), B - organic chemical industry (55 nodes), C - inorganic chemical industry (44 nodes), D - metal smelting and processing industry (16 nodes), E - lithium deep processing industry (21 nodes), and F - comprehensive waste utilization industry (10 nodes). Detailed information on each node is provided in Supplementary Data 1. The transition from the original structure to the optimal structure is shown in Supplementary Fig. 20. The node information for each industry is shown in Supplementary Tables 1–6. Source data are provided as a source data file.

**Fig. 2:**
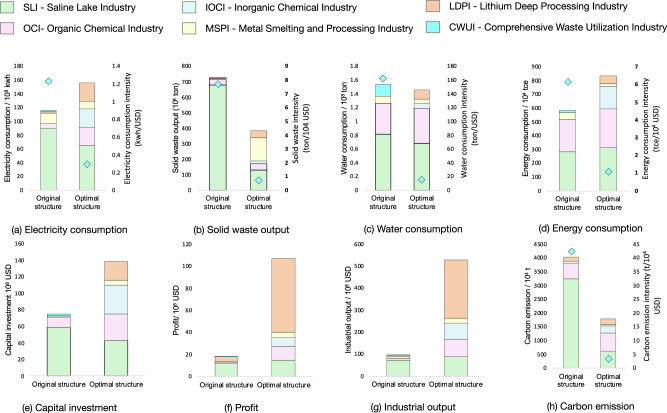
Indicators of the original structure and optimal structure of the saline lake industrial zone. Thirteen indicators are obtained by calculating the economic efficiency, environmental impact, and resource consumption of the original structure of the industry. After modeling, economic efficiency, such as industrial output and profit, of the whole industry improved, environmental impacts, such as carbon emission and solid waste output, decreased, and resource consumption, such as water consumption and energy consumption, decreased. The 13 indicators are electricity consumption (**a**), solid waste output (**b**), water consumption (**c**), energy consumption (**d**), capital investment (**e**), profit (**f**), industrial output (**g**), carbon emission (**h**), electricity consumption intensity (**a**, blue diamond), solid waste intensity (**b**, blue diamond), water consumption intensity (**c**, blue diamond), energy consumption intensity (**d**, blue diamond), and carbon emission intensity (**h**, blue diamond). The abbreviations for each industry are SLI - saline lake industry, OCI - organic chemical industry, IOCI - inorganic chemical industry, MSPI - metal smelting and processing industry, LDPI - lithium deep processing industry, and CWUI - comprehensive waste utilization industry. The indicators of the optimal structure of each node are shown in Supplementary Figs. 22–30. Source data are provided as a source data file.

Utilizing the RRIEDOM model, an assessment was conducted concerning the chemical industry’s initial configurations in the saline Qinghai Lake area. Figure 1 represents the original and optimal resource development patterns. The indicator analysis before and after optimization is shown in Fig. 2. Primary industries with low efficiency, elevated carbon emissions, and limited added value are predominant. Specifically, the gross industrial output value was 9.47 billion United States dollars (USD). This achievement is accompanied by significant electricity, water, and energy consumption of 116 × 10^8^ kWh, 1.54 × 10^8^ tons, and 5.82 million tons, respectively. Notably, the manufacturing of chemical products, including potash, polyvinyl chloride (PVC), methanol, and Li_2_CO_3_, accounted for 7.4 million tons, 2.2 million tons, 1 million tons, and 30 thousand tons, respectively. A comprehensive list of additional products is provided in the ‘sheet input-out matrix’ of Supplementary Data 1.

From the perspective of industrial categories, the primary drivers of industrial output were the saline lake industry (SLI) and organic chemical industry (OCI), contributing 76.32% and 10.4%, respectively; the remaining minor contributors were the inorganic chemical industry (IOCI), metal smelting and processing industry (MSPI), lithium deep processing industry (LDPI), and comprehensive waste utilization industry (CWUI), respectively. Moreover, SLI also emerged as the largest carbon emitter, responsible for 80.42% of emissions, equivalent to 32.31 million tons of CO_2_. Regarding energy consumption, SLI and OCI played pivotal roles, accounting for 48.54% and 39%, respectively, equivalent to 2.82 million tons and 2.33 million tons, respectively. Collectively, these sectors absorbed nearly 80% of the capital investment, totaling 5.9 billion USD, and, within the SLI industry, specializing in producing primary chemical products utilizing saline lake brine as a raw material.

From a technological standpoint, the original structure consisted of 24 distinct technologies and nodes. The potash from brine technology (labeled A2 in the network of Fig. 1) yielded a substantial production of 7.4 million tons of potassium chloride (KCl). The economic contributions within this network stemmed primarily from potash production technology, PVC production from calcium carbide technology, and calcium carbide production itself, at 27%, 28%, and 12%, respectively. Carbon emissions were attributed chiefly to calcium carbide PVC and calcium carbide production, accounting for 54% and 23%, respectively, of the total carbon emissions. Among the nodes, calcium carbide PVC (A12, in Fig. 1) emerged as the most significant energy consumer, utilizing 0.528 million tons of coal annually to support the production of 2.2 million tons of PVC, with the most significant capital investment of 4.85 billion USD. Concerning solid waste output, calcium carbide PVC (A12) and calcium carbide production (A14) constituted the two predominant contributors, accounting for 86.8% of the total solid waste output. Potash from brine, PVC from calcium carbide, and Li_2_CO_3_ production emerged as the top three profit-generating nodes, contributing to nearly 78% of the total network profit.

From the entire sectorial and technological view of the regional chemical industry, substantial potential for optimizing the utilization of local resources could be obtained by applying diverse technologies under the RRIEDOM framework.

### Benefit of optimizing the industrial structure for the regional economy

RRIEDOM determines the optimal structure with the input of the local resource endowment, including the size of each technology, the production of chemical products of the industrial network, and the recommended strategies for introducing technologies. The original optimal structure and indicators of both structures are shown in Figs. 1 and 2. After industrial structure optimization, 86 technologies (from a total of 358 available technologies from the database) and 70 chemical products were chosen by RRIEDOM to maximize the economic output while comprehensively utilizing local resources.

The optimal structural configuration yields an industrial output of 52.7 billion USD and a substantial profit of 10.7 billion USD, representing a 597% increase in industrial output compared to the original structure (industrial output of 9.49 billion USD). Notably, this transition is marked by a noteworthy reduction in critical metrics: electricity intensity plummeted from 1.22 to 0.29 kWh/USD, resulting in a mere 34% increase in electricity consumption. Furthermore, this transformation is characterized by significant reductions across various dimensions. The solid waste output notably decreased by approximately 34.5 million tons, while the electricity consumption intensity substantially decreased from 1.22 to 0.29 kWh/USD. Similarly, the intensity of solid waste output remarkably decreased from 7.67 to 0.73 tons per 10^4^ USD. Water consumption has been significantly curtailed by nearly 8 million tons, and water consumption intensity has substantially decreased from 162.2 to 14.97 tons per 10^4^ USD. The total carbon emissions are projected to decrease by an impressive 56%, decreasing from 37 to 17 million tons.

There is a discernible shift in economic contributions when examining this transition from an industry perspective. The original structure, predominantly SLI-dominated with a contribution of 76.32% amounting to 7.2 billion USD, is transformed into an LDPI-dominated structure, where the LDPI contributes 49.7%, which is equivalent to 26.3 billion USD. Furthermore, carbon emissions are transitioning from being primarily attributed to SLI, accounting for 80.4% and totaling 32.3 million tons, to being driven by OCI, which now accounts for 36.4% and totals 6.52 million tons.

The results of the optimal structure exhibited significant potential for energy savings, resource savings, and environmental protection. It is essential to investigate the possible transition path from the original structure to the optimal structure to reveal the properties of the different paths concerning the environment, resources, and the economy.

### Transition path from the original structure to the optimal structure

**Fig. 3:**
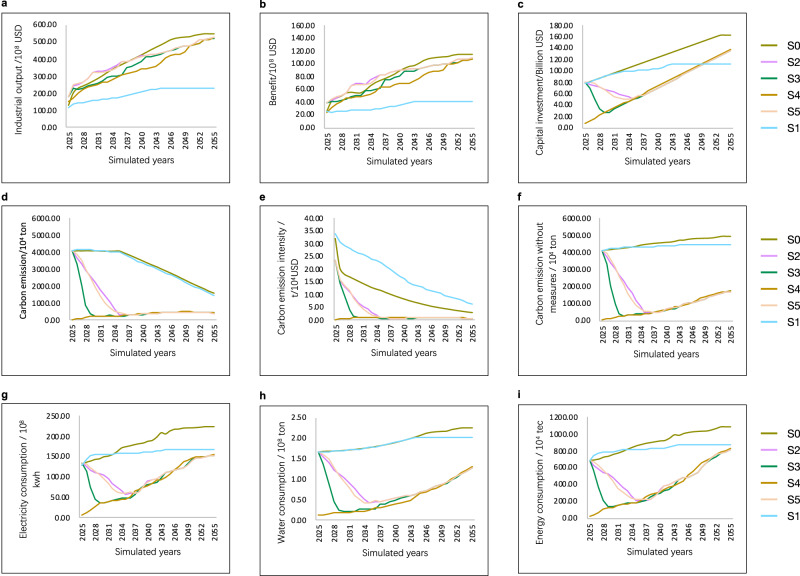
Results of 6 scenarios during a 30-year simulation of saline lake industries from 2025 to 2055. Six scenarios were designed using the resource-based regional industrial economy development optimization model (RRIEDOM) simulation, including S0, which was developed under the original structure and gradually introduced the technologies recommended by RRIEDOM; S1, which was developed with certain constraints on the industrial structure to simulate the empirical pattern; S2, which was developed under the original structure, allowing a 10-year transition to the optimal structure by identifying and eliminating the low-efficiency technologies and gradually deploying the technologies recommended by RRIEDOM; S3, which is similar to S2 but has a transition period of 5 years; S4, which assumes that there is no original structure and develops only under the guidance of RRIEDOM with the interaction of available technologies database; and S5, which is similar to S2 but has a longer transition period of 20 years. Through the simulation of six scenarios, six paths for the development of the Salt Lake Industrial Zone are obtained, and in the optimal development mode (S4 scenario), the whole development path has the least resource consumption, the least environmental impact, and the greatest economic benefits. The indicators are industrial output (**a**), benefit (**b**), capital investment (**c**), carbon emission (**d**), carbon emission intensity (**e**), carbon emission intensity without measures (**f**), electricity consumption (**g**), water consumption (**h**), and energy consumption (**i**). The supplementary results for each scenario are shown in Supplementary Figs. 31–35. Source data are provided as a source data file.

Six scenarios were designed to investigate the sustainable development path for the regional industry and to determine the optimal transition path from the original structure to the optimal structure. S0, which was developed under the original structure and gradually introduced the technologies recommended by RRIEDOM; S1, which was developed with certain constraints on the industrial structure to simulate the empirical pattern; S2, which was developed under the original structure, allowing a 10-year transition to the optimal structure by identifying and eliminating the low-efficiency technologies and gradually deploying the technologies recommended by RRIEDOM; S3, which is similar to S2 but with a transition period of 5 years; S4, which assumes that there is no original structure and develops only under the guidance of RRIEDOM with the interaction of available technologies database; and S5, which is similar to S2 but with a longer transition period of 20 years. Detailed scenario descriptions are provided in the “Supplementary Method 5 Scenario Settings” subsection of the Supplementary Information. A concise overview of the results from the six scenarios is shown in Fig. 3.

The empirical pattern represented by S1 exhibits the lowest economic output and benefits among the six scenarios. S0, S2, S3, S4, and S5 exhibit significant increases in accumulated industrial output and profit that far exceed the levels of S1 (Fig. 3a). However, the environmental impact and resource consumption of these scenarios are considered disparities. Due to its similar industrial structure to S1, S0 presents a favorable economic output but a high energy-cost and low-added value industrial structure, with elevated energy consumption ranging from approximately 91% to 221%, increased water consumption ranging from approximately 96% to 360%, and more significant carbon emissions ranging from approximately 98% to 931%, and it only achieves 48% to 58% of the economic benefits observed in other scenarios.

**Fig. 4:**
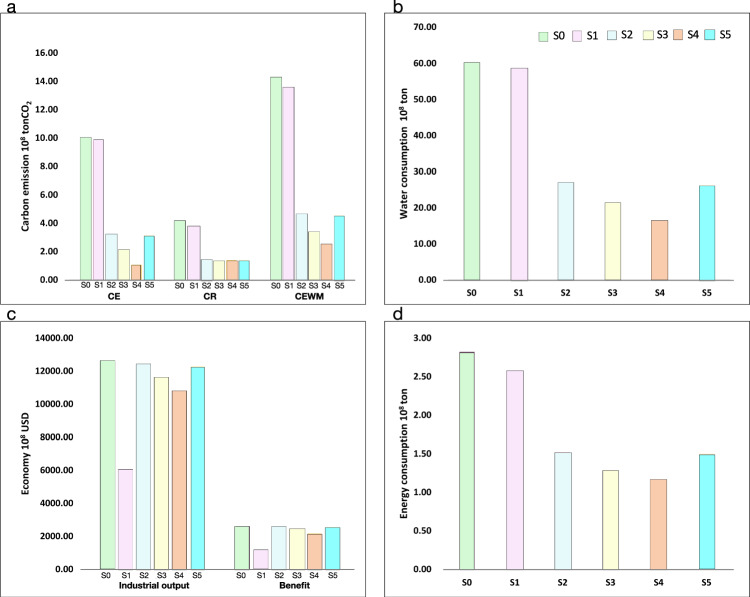
Accumulated indicators of saline lake industries under different scenarios. Analyzing the paths under 6 scenarios (S0 to S5), the scenarios (S0, S1) with no changes to the original industrial structure have greater cumulative resource consumption and environmental impacts, while the paths (S2, S3, S4, S5) with adjustments and optimization to the original industrial structure have greater economic benefits, indicating that the original industrial structure has the characteristics of high resource consumption and low value added. The indicators involved in the analysis include (**a**) carbon emissions with indicators of accumulated carbon emission (CE), accumulated carbon reduction (CR), and accumulated carbon emission without measures (CEWM); (**b**) water consumption with indicators of accumulated water consumption; (**c**) the economy with indicators of accumulated industrial output and accumulated benefit; and (**d**) energy consumption with indicators of accumulated energy consumption. Source data are provided as a source data file.

In scenarios S2, S3, S4, and S5, relatively low energy consumption, carbon emissions, and reduced water consumption with high economic output are obtained. Compared with the similar economic level of S0, carbon emissions decrease by approximately −8.78 to 0.19 × 10^8^ tons, water consumption decreases by approximately −42.11 to 1.9 × 10^8^ tons, and energy consumption decreases by approximately −1.41 to 0.24 × 10^8^ tons. For scenarios S2, S4, and S5, the environmental costs and resource consumption in these transition scenarios initially decrease during the early stage (2025–2035) and subsequently increase (2035–2055). This trend is attributed to the low sustainability of the current industrial system; once the optimal industrial structure is reached, the most efficient industrial development pattern, which is similar to S4, can be achieved by the RRIEDOM model. Notably, S4 is the scenario with no fundamental industry assumed by this study, which is the absolute sustainable solution compared with other transition scenarios. Figure 4 illustrates that the expeditious attainment of the optimal path yields considerable cumulative resource and environmental advantages. Compared to scenario S1, transitioning to the optimal path by 2035 can avert the accumulation of approximately 6.62 × 10^8^ tons of carbon emissions, conserve approximately 31.67 × 10^8^ tons of water resources, and generate additional profits of approximately 6.62 × 10^8^ USD.

Implementing alternative decarbonization measures such as CCUS, clean energy, and steam boiler retrofitting causes a gradual decline in carbon emissions across all scenarios. Compared with whether carbon mitigation measures were established (Fig. 3d, f), S2 to S4 experienced a notable reduction in carbon emissions, reaching their lowest points by 2030. Notably, even under the original structures represented by S0 and S1, carbon emissions are projected to decrease by 50% by 2050. However, we also discover that the carbon mitigation potential of these end-of-pipe measures is less effective than that of industrial transition at the same economic output level. For instance, by implementing carbon emissions reduction measures, a maximum reduction of merely 2.40 × 10^8^ tons of carbon emissions can be achieved across various scenarios (refer to Table 1). However, in the case of industrial restructuring, it becomes possible to prevent a minimum of 8.3 × 10^8^ tons of emissions. Due to the inevitability of accumulated emissions, selecting a technology optimization approach in the earlier stages proves to be more efficient than gradually implementing additional carbon reduction technologies. In essence, prioritizing cleaner production methods over subsequent end-of-pipe treatments is advantageous.

**Table 1:** Variation in cumulative indicators among the S0, S2–S5 scenarios and S1 scenarios

Variation	△(S0-S1)	△(S2-S1)	△(S3-S1)	△(S4-S1)	△(S5-S1)
Industrial output (10^8^ USD)	6573.94	6298.25	5511.21	4752.46	6259.75
Benefit (10^8^ USD)	1546.47	1515.97	1308.23	1090.33	1504.55
CE (10^8^ ton)	0.19	–6.62	–7.83	–8.78	–6.70
CR (10^8^ ton)	0.41	–2.34	–2.40	–2.37	–2.35
CEWM (10^8^ ton)	0.60	–8.96	–10.23	–11.16	–9.04
Water consumption (10^8^ ton)	1.90	–31.67	–37.44	–42.11	–32.04
Energy consumption (10^8^ tce)	0.24	–1.06	–1.29	–1.41	–1.07

The accumulated indicators include industrial output, benefit, accumulated carbon emission (CE), accumulated carbon reduction (CR), accumulated carbon emission without measures (CEWM), water consumption, and energy consumption; △ = variation compared with the S1 scenario, e.g., △(S0-S1) represents the difference between the indicators S0 and S1.

### Potential of alternative carbon reduction measures

**Fig. 5:**
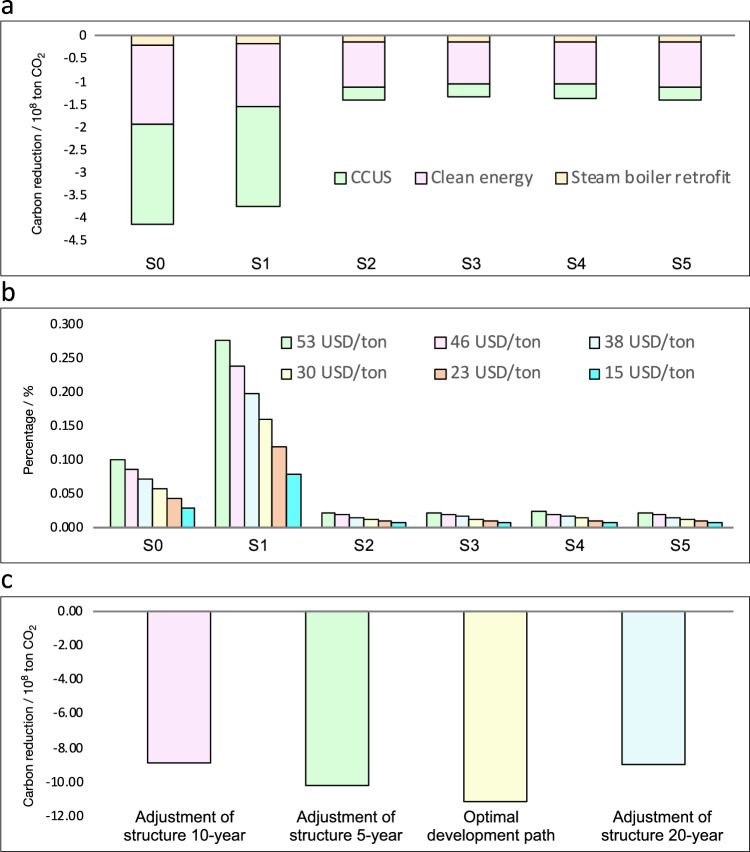
Carbon mitigation cost and benefit of saline lake industries by a resource-based regional industrial economy development optimization model (RRIEDOM). Three types of emission reduction methods were considered to achieve carbon reduction, namely, carbon reduction through carbon capture, utilization, and storage (CCUS), clean energy, and steam boiler retrofitting. The cost of carbon reduction and the amount of carbon reduction under different scenarios are simulated, in which CCUS under the S0 and S1 scenarios involves a greater contribution to carbon reduction, but due to the higher direct carbon emissions of these two paths, the corresponding cost of carbon reduction is also the highest, whereas the optimal path (S4) avoids the largest amount of carbon emissions. **a** CCUS, clean energy, and steam boiler retrofitting under the different scenarios, **b** ratio of the CCUS cost to the CCUS benefit under the different scenarios at the different CCUS prices, and **c** cumulative carbon reduction through the industrial transition. Source data are provided as a source data file.

In addition to technological optimization, the potential of alternative carbon reduction measures (ACRMs) must be addressed. We further discussed the variation in carbon reduction measures in terms of the method, cost-to-benefit ratio, and effectiveness (Fig. 5). Implementing carbon reduction technologies yields substantial reductions in carbon emissions across different scenarios. The emission reductions in these scenarios range from approximately 1.35 to 4.16 × 10^8^ tons, constituting approximately 27% to 56% of the total actual carbon emissions for each scenario.

For S0 and S1, adopting clean energy leads to significant reductions in emissions, which are approximately 42% and 38% of the total ACRM for scenarios S0 and S1, respectively. Additionally, the implementation of CCUS technology resulted in even more significant reductions, reaching approximately 53% and 58% for S0 and S1, respectively. For scenarios S2 to S5, the primary driver of carbon reduction is the deployment of clean energy, which contributes to approximately 68% to 70% of the overall reduction. This finding is primarily attributed to the relatively low direct carbon emissions in these scenarios, with the primary source of carbon emissions stemming from electricity production. In these scenarios, only a small portion of the carbon reduction can be attributed to steam boiler retrofits, accounting for approximately 4% to 10% of the respective emissions reductions.

Compared with clean energy and steam boiler retrofitting, CCUSs are alternative candidates for future emission reductions despite the significant consideration of associated costs. For a more comprehensive cost analysis, the expense of implementing CCUS for emission abatement is evaluated as a percentage of the overall profit within a price range spanning approximately 15–53 USD/ton, as shown in Fig. 5b. In S1, the ratio of achieving carbon emission reductions to profit ranges from 7.9–27.6% under the different CCUS prices. The difficulty in reducing emissions will significantly increase if the cost of CCUS remains high. When considering more stringent emission reduction policies, such as a zero-emission policy, the proportion could increase to 50%, which may prove financially untenable. In the optimal scenario, S4, the predominant carbon emissions stemmed from electricity consumption, with limited process-related emissions. Consequently, the proportion of carbon reduction achieved through CCUS remains relatively modest, and the cost-to-benefit ratio of CCUS ranges from 0.7% to 2.3%.

## Discussion

According to the RRIEDOM simulation, the paths of the different scenarios are minimal environmental impact and resource consumption under the corresponding development goals. Therefore, highly efficient technologies will be given top priority by RRIEDOM until the potential of such technologies is exhausted, and then subefficient technologies will be chosen^25^. Notably, there will be synergistic effects between high-efficiency technologies and subefficient technologies if subefficient technologies provide the necessary materials needed by high-efficiency technologies. Such synergistic effects can be visualized by analyzing industrial networks. For example, the technologies of saline brine extraction of lithium can lead to the development of potassium extraction technologies (A2, in Fig. 1) because the material of the former technology (old brine) can only be provided using the byproduct of the latter technology. In contrast, the cost of extraction directly from saline brine remains high, so the development of lithium extraction will synergistically promote potassium extraction. Consequently, a more efficient and diverse industrial symbiosis network can be constructed.

**Fig. 6:**
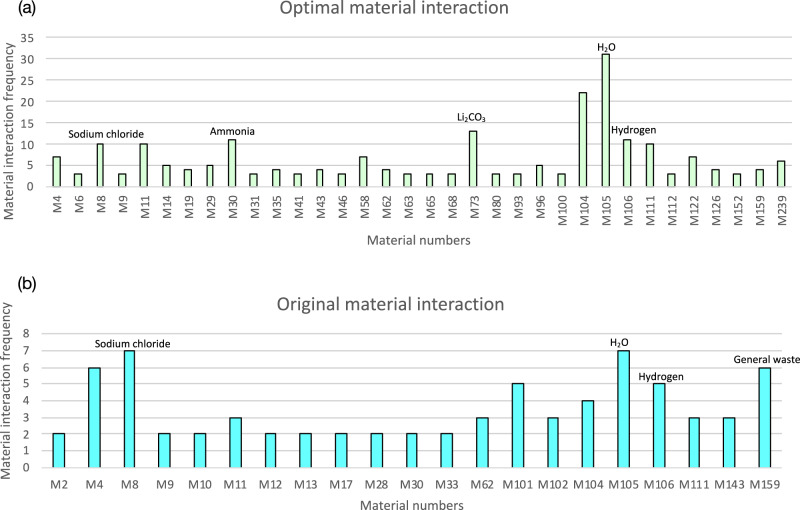
Frequency of material symbiosis for the original structure and the optimal structure. The material interactions in the original and optimal structures are analyzed, and the material interactions in the optimal structure (**a**) exhibit complex diversity compared with those in the original structure (**b**). For example, the number of material interactions for Li_2_CO_3_ in the optimal structure is 13, indicating that 13 exchanges of the input and output nodes occur due to Li_2_CO_3_-related technologies in the optimal structure. For the number of materials, refer to the Supplementary Data 1 file. The production of materials is shown in Supplementary Fig. 21. Source data are provided in a source data file.

To quantify the effectiveness of technological adjustment by RRIEDOM in terms of IS enhancements, a meticulous examination of the number of symbiotic materials within the material input‒output matrix was conducted. There are 21 types of materials (a total of 255 types of materials) that interact twice with other technologies in the original structure, whereas that of the optimal structure has 62 types of materials, indicating that the interaction links between the industrial network are strengthened after optimization. Materials such as ammonia (NH_3_), KCl, salt (NaCl), hydrogen (H_2_), lithium carbonate (Li_2_CO_3_), and sulfuric acid (H_2_SO_4_)_,_ which are traditional saline lake products, are the most common symbiotic materials in the optimal structure. The primary symbiotic materials and frequency of symbiosis in the industrial network are shown in Fig. 6.

Stakeholders prefer to select the alternative carbon reduction measures (ACRMs) mentioned in this study rather than adjust their original industrial structure^36,37^. This study confirms that the transition to the optimal structure for low-energy-consuming technologies will have better benefits than the subsequent deployment of carbon reduction measures. Instead of developing energy-intensive technologies at an early stage and employing carbon reduction measures to compensate for end-of-pipe treatment at a later stage, employing the optimal industrial structure at the early stage is an essential pattern of sustainable development to avoid carbon emissions. Notably, a longer-term transition (10 or 20 years) is recommended to promote resource consumption and increase efficiency.

The contribution of clean energy to carbon reduction was greater than that of CCUS under the optimal path. Achieving the 2 °C target in the Paris Agreement necessitates reducing the electricity factor to 15 g/kWh by the year 2050, assuming that all electricity consumption is sourced from the grid^38^. Despite the substantial gap in the widespread adoption of CCUS in China, the cost of CCUS remains prohibitive for extensive implementation^39,40^. A range of CCUS prices is assumed to determine the cost of carbon reduction within a region. The cost of decarbonizing the structure of high-energy-consumption technologies is high because the direct emissions from such technologies are significantly greater than those from the technologies in the optimal structure. However, the potential for clean energy in S1 and S0 has dissipated, and further decarbonization will show a greater reliance on CCUS and technological improvements.

**Fig. 7:**
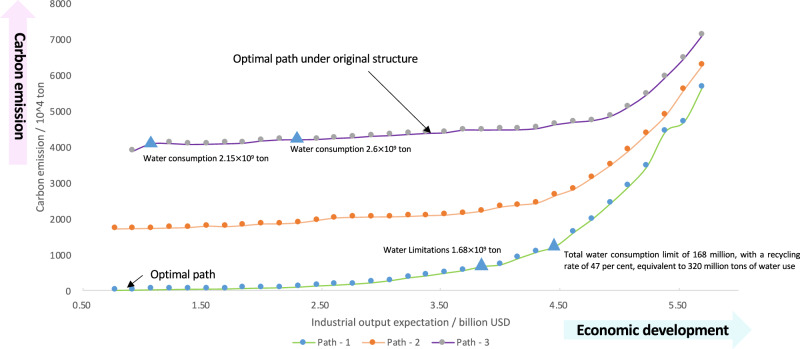
Original development path and optimal development path of saline lake industries. Three paths were designed to explore the properties of the different development paths. Path − 1 (purple solid line): Initial structure of the industrial area as a starting point, followed by resource-based regional industrial economy development optimization model (RRIEDOM) modeling to guide development. Path − 2 (orange solid line): Starting point with polyvinyl chloride (PVC) demand of 1 million tons, followed by RRIEDOM modeling. Path − 3 (green solid line): No initial structure setup, direct development using RRIEDOM modeling. For further discussion of Fig. 7, please refer to Supplementary Discussion 1 in the Supplementary Information. Source data are provided as a source data file.

Notably, there is an optimal path for regional industrial development under local resource endowment, which is composed of a set of deployed technologies. S4 is the optimal path for Qinghai Lake saline exploration with the highest resource efficiency among all the scenarios. Any deviation from such a path will result in more carbon emissions and higher resource consumption for the given development goal. To discuss how the basic industry affects the optimal path transition, three cases with different initial industries for comparison of carbon emissions were selected, namely, with no initial industry, an initial industry with only 1 million tons of PVC, and an initial composition of the local industrial structure. As shown in Fig. 7, the green curve, orange curve, and purple curve represent three cases of initial industries, which have no relatively minor scale of initial industry or large scale of initial industry, respectively. With economic development, all cases of carbon emissions exhibit increasing trends; additionally, the growth rate gradually accelerates with economic growth. The priorities of industrial technologies with low-carbon, high-economic value are selected by RRIEDOM, guided at the first stage of economic growth. Subsequently, the growth rate of carbon emissions continued to accelerate due to the arrival of resource bottlenecks in the development of highly efficient technologies, where supporting upstream industries with low economic value and high carbon emissions must follow. With the continuous development of the economy, the carbon emission intensity of all paths will eventually approach and reach the optimal development model, but a large gap in accumulated carbon emissions still exists, which can be inferred from the integral difference among the three different paths and the comparison of the accumulated carbon emission gaps between S5 and S4, as shown in Fig. 4. Thus, the greater the deviation of the current industry system from the optimal development path is, the greater the accumulated cost and input that should be paid during industrial transformation, indicating the necessity of planning the layout of industrial structure optimization as soon as possible.

Most studies on industrial symbiosis networks have focused only on a single material, such as water, energy, or another specific material^41,42^. It is difficult to provide a comprehensive perspective on the planning and utilization of resources, which is only suitable for making minor adjustments and improvements after a complete industrial structure has been established. However, it is difficult to explore how to optimize the use of resources to achieve sustainable development from a global perspective. Previous studies, including the proposal of interactive inference and optimization, need more technical databases to support this idea^14^. In contrast, the process of interactive inference is complicated and prone to problems such as combinatorial explosions^14,15^. The model has been enhanced to identify suitable industrial chains for large-scale regional resource development. The model provides optimal strategies for resource development and technology deployment. The constructed network encompasses selection relationships for more than 200 types of materials, mitigating empirical issues associated with expert manual selection^15^.

This study has established many complex industrial networks and strategies for the development of local resources. Notably, the demand constraint was not incorporated into the model because its objective is to maximize resource utilization and regional scope. However, promising technologies, such as low-technology readiness level technologies (low-TRL), are not included in establishing the available technology database. For example, technologies for ammonia production were selected based on coal and natural gas consumption. Current studies on the use of ammonia from sustainable energy sources have been widely reported^43–45^. These types of technologies are not included in this database because of the consideration of instant applications. After the development and massive commercial application of low-TRL technologies, these technologies can be selected in the database to plan the optimal path. The incorporation of these promising technologies will promote high-efficiency networks and improve resource efficiency and decarbonization.

## Methods

**Fig. 8:**
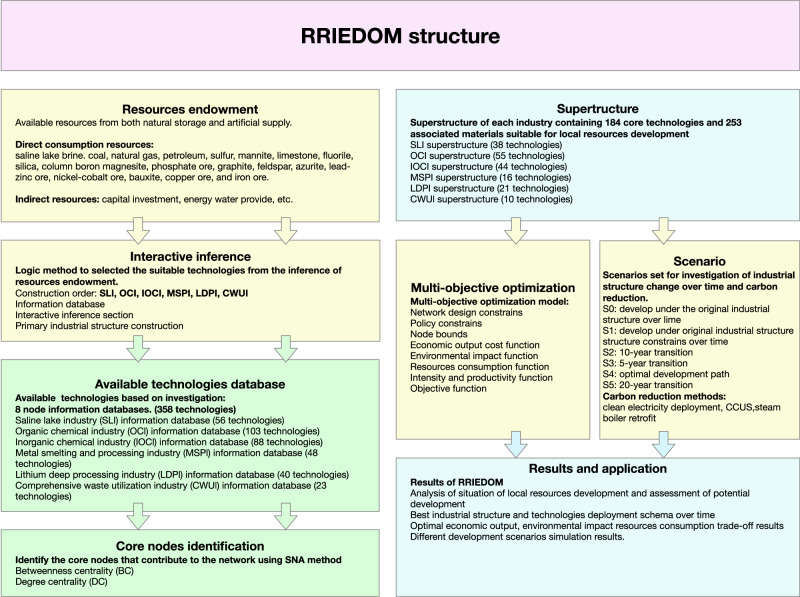
Framework of the resource-based regional industrial economy development optimization model (RRIEDOM). RRIEDOM integrates the various approaches of resource endowment, interactive inference, mathematical optimization of technical databases, social network analysis (SNA), and systems engineering superstructure for quantitative and systematic regional resource development of the saline lake industry (SLI), organic chemical industry (OCI), inorganic chemical industry (IOCI), metal smelting and processing industry (MSPI), lithium deep processing industry (LDPI), and comprehensive waste utilization industry (CWUI).

There are seven sections of RRIEDOM, as shown in Fig. 8: resource endowment investigation, establishment of the node information database, interactive inference, establishment of available technologies database, core node identification, construction of superstructure, multiobjective optimization, and scenario setting. The following section provides a brief description of each section. A detailed description of the RRIEDOM model is provided in the ‘Supplementary Method 3 Optimization Mathematical Model’ subsection of the Supplementary Information.

### Resource endowment survey

Resource endowment investigations necessitate a comprehensive examination of the types and quantities of regional bulk resources, including ores, coal, petroleum, and saline lake brine. In this study, field research was conducted in the saline Qinghai Lake circular economy industrial zone.

The location of the resources that need to be planned and developed should be determined; the information about the resources that can be developed in the target area should be determined through field research, government consultation, enterprise research, expert interviews, etc.; and a database should be constructed. This includes the types of resources that can be developed, potential resource reserves, and the maximum amount of resources that can be developed each year.

Through a field study of the Qinghai Salt Lake Industrial Zone, 19 natural resources were identified as resource endowments. Salt lake brine, coal, oil, natural gas, lead-zinc ore, and limestone are the resources to be developed, and through the data collection, it was determined that the carnallite’s current maximum amount of exploitation per year is 0.17 billion tons.

### Node information base construction

The establishment of a node information database requires a comprehensive investigation of current technologies related to the production of chemical products via literature and government documents.

The overarching objective is to delineate the industry sectors within the designated geographic region that hold potential for exploitation and, subsequently, to establish a comprehensive database.

The procedural framework encompasses the following steps: (1) delimiting industry categories, encompassing the salt lake chemical industry, organic chemical industry, inorganic chemical industry, metal processing industry, lithium power industry, and waste treatment industry; (2) creating node information databases for each industry; and (3) formulating the database via a comprehensive literature review, on-site investigation, and expert interviews.

As exemplified by an extensive series of on-site investigations, a collective sum exceeding 358 nodes was successfully identified, including 6 distinct industrial categories. Detailed node information is provided in the Supplementary Data 1 file.

### Interaction inference

The primary objective is to amalgamate data sourced from the node information database to formulate the network superstructure specific to each respective industry.

The procedure entails the following steps: (1) identifying and categorizing natural resources, (2) organizing and amalgamating these identified resources, (3) correlating nodes, and (4) establishing the network structure, among other pertinent actions.

### Core node identification

Social network analysis (SNA) was conducted during this period. SNA was initiated in the 1920s to focus on the relationships between different social entities^46^. In this study, two major indicators in SNA were applied: betweenness centrality (BC) and degree centrality (DC)^47,48^. The DC represents the number of nodes linking to other nodes, where in this study, it is the number of links of nodes that acquire materials from other nodes and the number of links that deliver materials to other links. The nodes with a high BC measure the degree of the node’s control over the core link utilization; if the node with a high BC fails at one point of operation, it will affect the operation of multiple industrial links and may cause cascading failures^27,49^. The BC and DC results for each node are shown in Supplementary Figs. 13–18 and in Supplementary Tables 7–12.

1 $${{DC}}_{i}=\frac{{{{{{\rm{deg}}}}} (i)}}{N-1}$$

where deg(i) is the degree of node i, i.e., the number of edges directly connected to node i, and N is the total number of nodes in the network.

2 $${{BC}}_{i}={\sum}_{i\ne s\ne t}\frac{{\sigma }_{st}(i)}{{\sigma }_{st}}$$

where i is a node in the network, ${\sigma }_{{st}}$ is the number of shortest paths from node s to node t, and ${\sigma }_{{st}}(i)$ is the number of paths passing through node i in these shortest paths.

### Construction of the superstructure

**Fig. 9:**
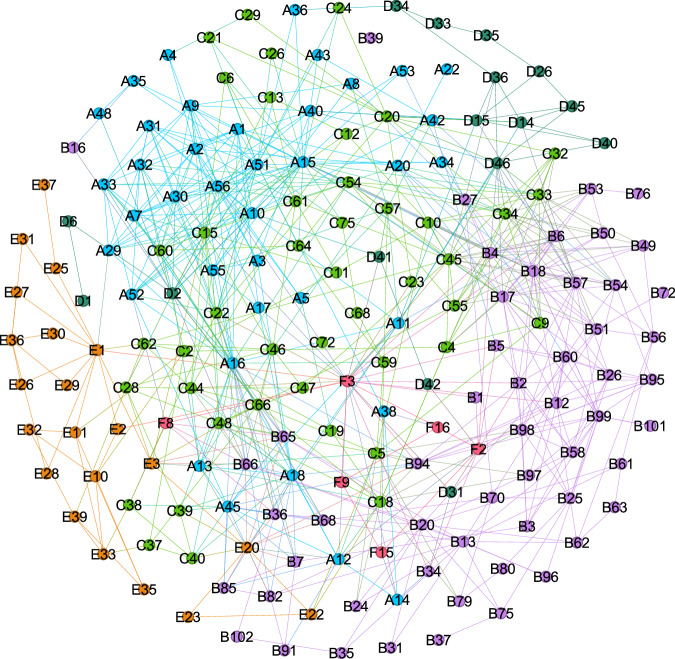
Superstructure of the core industrial network. The databases in the technology database are modeled to represent all industrial structures that may be involved in the development process of the corresponding region. A- saline lake industry, B- organic chemical industry, C- inorganic chemical industry, D- metal smelting and processing industry, E- lithium deep processing industry, and F- comprehensive waste utilization industry. Nodes represent specific chemical industry technologies. Links are possible material exchanges between the nodes. The structure of each industry is shown in Supplementary Figs. 7–12.

A network composed of core nodes was obtained after applying SNA to a comprehensive industrial network. This core network is divided into six industrial superstructures: SLI (38 nodes), OCI (55 nodes), IOCI (44 nodes), MSPI (16 nodes), LDPI (21 nodes), and CWUI (10 nodes). In total, 184 nodes were selected in the core network, with 709 links of possible material exchange. Considering the intricate nature of visualizing the process network, a superstructure visualization is defined, as illustrated in Fig. 9. The colors denote the different categories, such as the industry category; the size of each node indicates the property of nodes, such as scale, CO_2_ emissions, energy consumption, and water consumption; and the links show the possible material exchange between two nodes. Other materials related to a node that do not participate in an exchange are removed in this type of visualization for simplicity.

### Multiobjective optimization

The framework of the optimization section of the core industrial network is illustrated in the ‘Supplementary Method 3 Optimization Mathematical Model’ subsection of the Supplementary Information. The optimization mathematical model is a multiobjective NLP model consisting of eight parts: (i) network design constraints for the production of all nodes and resources; (ii) policy constraints of parks in different years; (iii) constraints of node bounds; (iv) economic output and cost functions of parks, industries, and node levels; (v) environmental impact functions of parks, industries, and node levels; (vi) resource consumption functions of parks, industries, and node levels; (vii) intensity and productivity functions of parks; and (viii) objective functions. The basic target of such a model is to utilize natural resources through the core industrial network to produce the main chemical products while minimizing the environmental impact and resource consumption and maximizing the economic output with limited capital investment and available technology databases.

### Scenario setting

Six scenarios were developed to explore the optimal and transition paths for the simulation of local resource development, labeled S0 to S5.

S0 was developed under the original structure and gradually introduced the technologies recommended by RRIEDOM;

S1 was developed with certain constraints on the industrial structure to simulate the empirical pattern;

S2 was developed under the original structure, allowing a 10-year transition to the optimal structure by identifying and eliminating low-efficiency technologies and gradually deploying the technologies recommended by RRIEDOM;

S3 was similar to S2 but had a transition period of 5 years;

S4 assumes that there is no original structure and develops only under the instructions of RRIEDOM with the interaction of available technology databases;

S5 was similar to S2 but had a longer transition period of 20 years.

In response to increasingly stringent climate policies, three potential carbon reduction strategies have been evaluated: clean energy adoption, CCUS implementation, and steam boiler retrofitting. Scenarios S2, S3, and S5 represent transition paths, while scenario S4 signifies the optimal path.

A detailed description of the scenario settings is provided in the ‘Supplementary Method 5 Scenario setting’ subsection of the Supplementary Information. A detailed description of the scenario settings is shown in Supplementary Fig. 19.

For more detailed information about RRIEDOM, please refer to Supplementary Information Methods 1–5, which provide a detailed description of the model. The Supplementary Data 1 file is also provided.

### Data collection

To substantiate the research presented in this article, we required a substantial volume of technical data. We collected detailed technical data from diverse sources, including the Intergovernmental Panel on Climate Change (IPCC)^50^, China’s National Bureau of Statistics^51^, China’s industry standards^52^, government industry reports^53,54^, park industry reports^54^, and project environmental impact assessment documents. The related data are provided in the Supplementary Data 1 file. For more information on the data, please refer to Supplementary Discussion 2.

### Reporting summary

Further information on research design is available in the Nature Portfolio Reporting Summary linked to this article.

### Supplementary information

Supplementary Information

Description of Additional Supplementary Files

Supplementary Data 1

Reporting Summary

### Source data

Source data

## Data Availability

The source data underlying Figs. 1–6 are provided as a source data file. All data used for this analysis are provided in the Supplementary Data 1 file. Source data are provided with this paper.
