# Supplementary material for: Integrated optimization modelling framework for low-carbon and green regional transitions through resource-based industrial symbiosis
Source: Nat Commun. 2024 May 7;15:3842. doi: 10.1038/s41467-024-48249-6 (PMC11076570; doi:10.1038/s41467-024-48249-6)
Supplement: Supplementary file 1 — Supplementary Information [file 41467_2024_48249_MOESM1_ESM.pdf]

Supplementary Information (SI) for

Integrated Optimisation Modelling Framework for Low Carbon and Green Regional Transitions Through Resource-based Industrial Symbiosis

Xin Xie[1], Hang Fu[1,2], Qisheng Zhu[1], Shanying Hu[1,*]

1, Center for Industrial Ecology, Department of Chemical Engineering, Tsinghua University, Beijing, 100084, China

2, Watershed Carbon Neutrality Institute, School of Resources&Enviroment,Nanchang University, Nanchang, 330031,China

∗ Corresponding author: Prof. Shanying Hu

Phone & Fax: (+)86 10 62794513

E-mail: hxr-dce@tsinghua.edu.cn

This file includes

Supplementary Figure 1-41, Supplementary Table 1-14, Supplementary Note 1-3, Supplementary Method 1-5, Supplementary Discussion 1-2, and Supplementary References.

Contents

CONTENTS **2**

SUPPLEMENTARY METHODS **3**

Supplementary Method 1 Model introduction 3
Supplementary Method 2 Nomenclature 4
Supplementary Method 3 Optimization mathematical model 12
Supplementary Method 4 Method of the construction of eco-industrial network 23
Supplementary Method 5 Scenario settings 56

SUPPLEMENTARY NOTES **74**

Supplementary Note 1 Detailed information of Qinghai saline lake industrial zone 74
Supplementary Note 2 Support of carbon reduction measures 77
Supplementary Note 3 Sensitive analysis 80

SUPPLEMENTARY DISCUSSIONS **83**

Supplementary Discussion 1 Optimal paths and industrial symbiosis times for RRIEDOM decision making 83
Supplementary Discussion 2 Data description 92

SUPPLEMENTARY REFERENCES **94**

Supplementary Methods

Supplementary Method 1 Model introduction

In this study, we introduce a resource utilization model known as the resource-based regional industrial economy development optimization model (RRIEDOM). This model employs a multi-method approach, integrating multi-objective mathematical optimization, super-structure networks, network analysis, material flow analysis, and scenario analysis. RRIEDOM utilizes a built-in technical database to determine the optimal industrial structure based on the resource endowment of the target region and offers insights into the optimal transition path from the existing local industrial structure to the desired one. It operates as a bottom-up integrated model.

There are seven sections of RRIEDOM: resource endowment investigation, establishment of the node information database, interactive inference, establishment of available technologies database, core nodes identification, construction of superstructure, multi-objective optimization, and scenario setting.

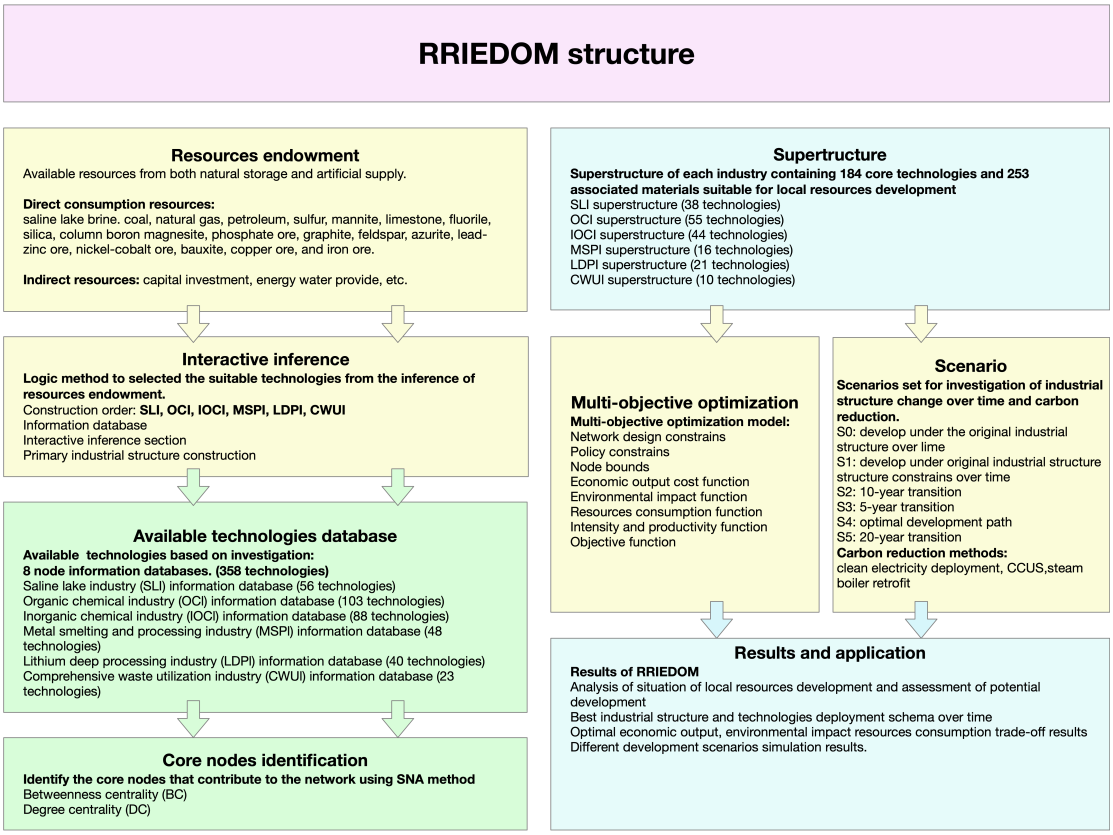

Supplementary Figure 1 Framework of the resource-based regional industrial economy development optimization model (RRIEDOM). RRIEDOM integrates the various approaches of resource endowment, interactive inference, mathematical optimization of technical databases, social network analysis (SNA), and systems engineering superstructure for quantitative and systematic regional resource development of saline lake industry (SLI), organic chemical industry (OCI), inorganic chemical industry (IOCI), metal smelting and processing industry (MSPI), lithium deep processing industry (LDPI), and comprehensive waste utilization industry (CWUI).

Supplementary Method 2 Nomenclature

Parks

park-1	Geermu industrial park
park-2	Dachaidan industrial park
park-3	Delhi industrial park

park-4	Ulan industrial park

Sets

i	nodes	/1*184/
j	resources	/1*255/
a	parks	/park-1, park-2, park-3, park-4/
t	years	/2025*2055/
k	energy category	/steam, diesel oil, biomass, coke, coal/
M	industry category	/Saline lake industry, Organic chemical industry, Inorganic chemical industry, Metal smelting and processing industry, Lithium deep processing industry, Comprehensive waste utilization industry/

Subsets

$Node_{i,m}$	Nodes in the industry category m
$Outreso_{i,j}$	Output of resource j of node i
$Inreso_{i,j}$	Input of resource j of node i

Variables

$x_{i,a,t}$	The size factor of node i in park a of year t

Park-level

$TGV_{a,t}$	Gross value of industrial output of park a in year t

$TCE_{a,t}$	Total amount of carbon emission of park a in year t
$TWC_{a,t}$	Total amount of freshwater consumption of park a in year t
$TEC_{a,t}$	Total amount of electricity consumption of park a in year t
$TSG_{a,t}$	Total amount of solid waste output of park a in year t
$TIN_{a,t}$	Total amount of capital investment of park a in year t
$TP_{a,t}$	Total amount of profit of park a in year t
$TEN_{a,t}$	Total amount of energy consumption of park a in year t
$TWW_{a,t}$	Total amount of sewage of park a in year t
$TWWI_{a,t}$	Total amount of investment of sewage infrastructure of park a in year t
$TWWE_{a,t}$	Total amount of electricity consumption of sewage infrastructure of park a in year t
$TWWAC_{a,t}$	Total amount of annual cost of sewage infrastructure of park a in yeart
$TWI_{a,t}$	Freshwater consumption intensity of park a in year t
$TCI_{a,t}$	Carbon emission intensity of park a in year t
$TELI_{a,t}$	Electricity consumption intensity of park a in year t
$TENI_{a,t}$	Energy consumption intensity of park a in year t
$TSI_{a,t}$	Solid waste output intensity of park a in year t
$TWP_{a,t}$	Freshwater productivity of park a in year t
$TEP_{a,t}$	Energy productivity of park a in year t

Industry-level

$GV_{a,t,m}$	Gross value of industrial output of industry m in park a in year t

$CE_{a,t,m}$	Total amount of carbon emission of industry m in park a in year t
$WC_{a,t,m}$	Total amount of freshwater consumption of industry m in park a in year t
$EC_{a,t,m}$	Total amount of electricity consumption of industry m in park a in year t
$SG_{a,t,m}$	Total amount of solid waste output of industry m in park a in year t
$IN_{a,t,m}$	Total amount of capital investment of industry m in park a in year t
$P_{a,t,m}$	Total amount of profit of industry m in park a in year t
$EN_{a,t,m}$	Total amount of energy consumption of industry m in park a in year t
$WW_{a,t,m}$	Total amount of sewage of industry m in park a in year t
$WWI_{a,t,m}$	Total amount of investment of sewage infrastructure of industry m in park a in year t
$WWE_{a,t,m}$	Total amount of electricity consumption of sewage infrastructure of industry m in park a in year t
$WWAC_{a,t,m}$	Total amount of annual cost of sewage infrastructure of industry m in park a in yeart
$WI_{a,t,m}$	Freshwater consumption intensity of industry m in park a in year t
$CI_{a,t,m}$	Carbon emission intensity of industry m in park a in year t
$ELI_{a,t,m}$	Electricity consumption intensity of industry m in park a in year t
$ENI_{a,t,m}$	Energy consumption intensity of industry m in park a in year t
$SI_{a,t,m}$	Solid waste output intensity of industry m in park a in year t

$WP_{a,t,m}$	Freshwater productivity of industry m in park a in year t
$EP_{a,t,m}$	Energy productivity of industry m in park a in year t

Node-level

$NGV_{a,t,i}$	Gross value of industrial output of node i in park a in year t
$NCE_{a,t,i}$	Amount of carbon emission of node i in park a in year t
$NCEE_{a,t,i}$	Amount of carbon emission due to the electricity consumption of node i in park a in year t
$NCES_{a,t,i}$	Amount of carbon emission due to the steam heating of node i in park a in year t
$NCED_{a,t,i}$	Amount of carbon emission due to the direct emission from the process of node i in park a in year t
$NCEO_{a,t,i}$	Amount of carbon emission due to other fuels consumption of node i in park a in year t
$NWC_{a,t,i}$	Amount of freshwater consumption of node i in park a in year t
$NEC_{a,t,i}$	Amount of electricity consumption of node i in park a in year t
$NSG_{a,t,i}$	Amount of solid waste output of node i in park a in year t
$NIN_{a,t,i}$	Amount of capital investment of node i in park a in year t
$NP_{a,t,i}$	Amount of profit of node i in park a in year t
$NEN_{a,t,i}$	Amount of energy consumption of node i in park a in year t
$NWW_{a,t,i}$	Amount of sewage of node i in park a in year t
$NEXP_{a,t,i}$	Cost of node i of park a in year t

$NEC_{a,t,i}$	Cost of energy consumption of node i of park a in year t
$NRC_{a,t,i}$	Cost of raw material of node i of park a in year t
$NSWC_{a,t,i}$	Cost of solid waste disposal of node i of park a in year t
$NFWC_{a,t,i}$	Cost of freshwater consumption of node i of park a in year t
$NIDC_{a,t,i}$	Cost of investment depreciation of node i of park a in year t
$NOVC_{a,t,i}$	Other variable costs of node i of park a in year t

Parameters

PP_j	Current price of resource j
$PEP_{j,a,t}$	Amount of initial supply of resources j of park a in year t
$PN_{i,j}$	Amount of input/output of resource j of node i with respect to the reference resource per unit production
PWC_i	Amount of freshwater consumption of node i with respect to the reference resource per unit production
$PWCF_i$	Factors of sewage output of node i
$PSWO_i$	Amount of solid waste output of node i with respect to the reference resource per unit production
$PEEC_i$	Amount of electricity consumption of node i with respect to the reference resource per unit production
$PINC_i$	Amount of capital investment of node i with respect to the reference resource per unit production
$PEEC_{k,m}$	Cost of the use of energy k per unit

$PFWC$	Cost of industrial water use
$PNEC_i$	Cost of energy consumption of node i of park a in year t with respect to the reference resource per unit production
PRC_i	Cost of resources consumption of node i of park a in year t with respect to the reference resource per unit production
$PSWC_i$	Cost of solid waste disposal of node i of park a in year t with respect to the reference resource per unit production
$PFWC_i$	Cost of freshwater consumption of node i of park a in year t with respect to the reference resource per unit production
$PIDC_i$	Cost of investment depreciation of node i of park a in year t with respect to the reference resource per unit production
$POVC_i$	Other variable costs of node i of park a in year t with respect to the reference resource per unit production
$PEC_{i,k}$	Amount of consumption of energy k of node i with respect to the reference resource per unit production
PCC_k	Carbon emission factor of energy k
$PCEP_{a,t}$	Constraint of energy productivity of park a in year t
$PCWP_{a,t}$	Constraint of freshwater productivity of park a in year t
$PCWI_{a,t}$	Constraint of freshwater consumption intensity of park a in year t
$PCEI_{a,t}$	Constraint of energy consumption intensity of park a in year t
$PTWC_{a,t}$	Constraint of total fresh water consumption of park a in year t
$PLBN_{a,t,i}$	Lower bound of node i of park a in year t

$PUBN_{a,t,i}$	Upper bound of node i of park a in year t
$MINE_{a,t}$	Minimal economic growth goal of park a in year t
$MAXIN_{a,t}$	Maximal capital investment input of park a in year t
POD_i	Period of depreciation of node i
$PWWI$	Sewage infrastructure capital investment per unit sewage disposal
$PWWA$	Sewage infrastructure annual operational cost per unit sewage disposal
$PWWE$	Sewage infrastructure annual electricity consumption per unit sewage disposal

Supplementary Method 3 Optimization mathematical model

The complete non-linear programming (NLP) model, as shown in Supplementary Figure 2, consists of: (i) the network design constraints for the production of all nodes and resources, (ii) the policy constraints of parks in different years, (iii) the constraints of nodes bounds, (iv) the economic output and cost functions of parks, industries, and nodes levels, (v) the environmental impact functions of parks, industries, and nodes levels, (vi) the resources consumption functions of parks, industries, and nodes levels, (vii) the intensity and productivity functions of parks, (viii) objective function.

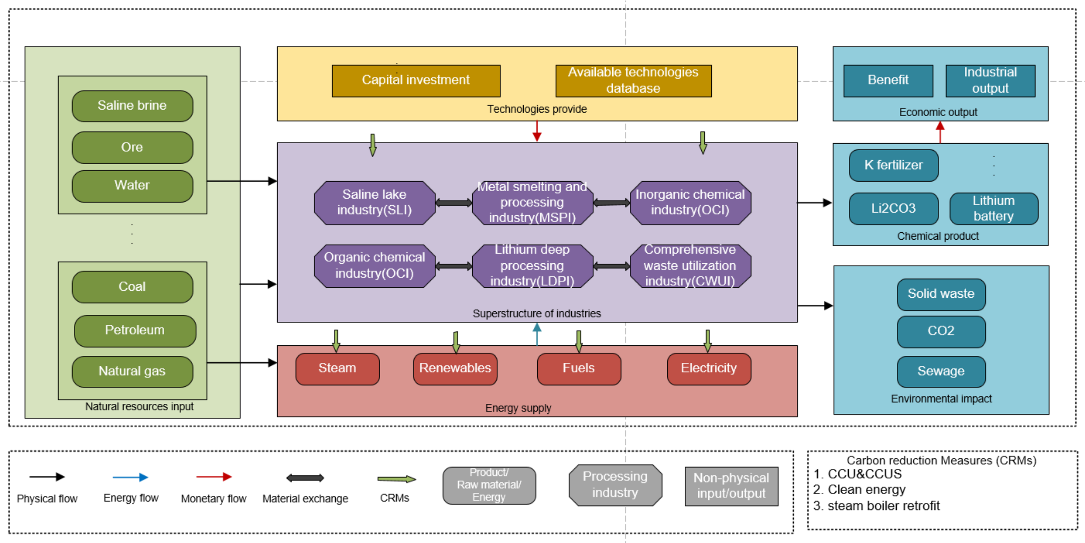

Supplementary Figure 2 Framework of the multi-objective optimization model. This mathematical optimization model utilizes natural resources, including saline lake brine, various types of ores, coal, oil, and natural gas, processed into chemicals, such as potash, lithium carbonate, ammonia, and lithium batteries, etc. The model includes modeling of capital investment, energy consumption, waste emissions, and economic development.

Network Design Constraints

Equations for superstructure network design, that allow all the consumptions of resource j of all nodes to be constrained are shown in Equation (1). The total amount of the consumption of resource j does not be allowed to exceed the initial supply amount of resource j. $PN_{i,j}$ is the element of a sparse matrix that saves the information of nodes' input/output associated with resources.

$$\sum_{i} PN_{i,j}x_{i,a,t} + PEP_{j,a,t} \geq 0 \ \forall j,a,t \quad (1)$$

Policy, Economic, and Resources Consumption Constraints

Equations for policy constraints, that allow constraint of the minimal economic goal, max capital investment, resources consumption, park-level intensity function of resources consumption and contaminants output, and park-level productivity function of resources consumption are shown in Equation (2) to (8). The intensity and productivity of parks should comply with the statutory regulations stipulated by the government. Each park has an economic development target and a total fixed capital investment limit.

$$TWI_{a,t} \leq PCWI_{a,t} \ \forall a,t \quad (2)$$

$$TENI_{a,t} \leq PCEI_{a,t} \ \forall a,t \quad (3)$$

$$TWP_{a,t} \geq PCWP_{a,t} \ \forall a,t \quad (4)$$

$$TEP_{a,t} \geq PCEP_{a,t} \ \forall a,t \quad (5)$$

$$TWC_{a,t} \leq PTWC_{a,t} \ \forall a,t \quad (6)$$

$$TGV_{a,t} \geq MINE_{a,t} \ \forall a,t \quad (7)$$

$$TIN_{a,t} \leq MAXIN_{a,t} \ \forall a,t \quad (8)$$

Nodes Bounds

Equation (9) set the lower and upper bounds of nodes of all nodes. During the optimization, a node may change its size between the lower and upper bounds. The upper bounds are set according

to the current maximal commercial capacity of related technologies.

$$PLBN_{a,t,i} \leq x_{i,a,t} \leq PUBN_{a,t,i} \ \forall a,i,t \tag{9}$$

<u>*Economic Output and Cost Functions*</u>

Park-level

There are three indicators (gross value of industrial output ($TGV_{a,t}$), total capital investment ($TIN_{a,t}$), total profit ($TP_{a,t}$)) selected as the description of industrial park economic development, These three indicators are calculated according to Equation (10) to (12).

$$TGV_{a,t} = \sum_m GV_{a,t,m} \ \forall a,t \tag{10}$$

$$TIN_{a,t} = \sum_m IN_{a,t,m} \ \forall a,t \tag{11}$$

$$TP_{a,t} = \sum_m P_{a,t,m} \ \forall a,t \tag{12}$$

Industry-level

A total of six industry categories are involved in the superstructure of network optimization, which are the saline lake industry (SLI), organic chemical industry (OCI), inorganic chemical industry (IOCI), metal smelting and processing industry (MSPI), lithium deep processing industry (LDPI), and comprehensive waste utilization industry (CWUI). The gross value of industrial output ($GV_{a,t,m}$), capital investment ($IN_{a,t,m}$), and profit ($P_{a,t,m}$) for each industry is the sum of its subset of nodes. The relevant equations are shown below from Equation (13) to (15).

$$GV_{a,t,m} = \sum_i NGV_{a,t,i} \ \forall \mathrm{a,t,i} \in Node_{i,m} \tag{13}$$

$$IN_{a,t,m} = \sum_i NIN_{a,t,i} \ \forall \mathrm{a,t,i} \in Node_{i,m} \tag{14}$$

$$P_{a,t,m} = \sum_i NP_{a,t,i} \ \forall \mathrm{a,t,i} \in Node_{i,m} \tag{15}$$

Node-level

The economic dimension of each node is described in terms of the gross value of industrial

output ($NGV_{a,t,i}$), profit ($NP_{a,t,i}$), and capital investment ($NIN_{a,t,i}$). In regard to $NGV_{a,t,i}$, the economic value of resources out from node i is considered and calculated with the size factor according to the current price. As for $NP_{a,t,i}$. The fixed cost and variable cost ($NEXP_{a,t,i}$) of each node (including raw material cost ($NRC_{a,t,i}$) , investment depreciation ($NIDC_{a,t,i}$), water use cost ($NFWC_{a,t,i}$), solid waste treatment cost ($NSWC_{a,t,i}$), energy use cost ($NEC_{a,t,i}$), other costs ($NOVC_{a,t,i}$)) can be calculated by Equation (16) to (24), and the profit of node i is the total output value minus the total cost of the production process. And $NIN_{a,t,i}$, the capital investment of node i, is calculated based on reference resource per unit production with size factor.

$$NGV_{a,t,i} = \sum_{j} Outreso_{i,j}\, x_{i,a,t} PP_j \;\forall a,t,i \tag{16}$$

$$NP_{a,t,i} = \sum_{j} Outreso_{i,j}\, x_{i,a,t} PP_j - NEXP_{a,t,i} \;\forall a,t,i \tag{17}$$

$$NEXP_{a,t,i} = \big(NRC_{a,t,i} + NIDC_{a,t,i} + NFWC_{a,t,i} + NSWC_{a,t,i} + NEC_{a,t,i} + NOVC_{a,t,i}\big) x_{i,a,t} \;\forall a,t,i$$

$$NRC_{a,t,i} = \sum_{j} Inreso_{i,j} x_{i,a,t} PP_j \;\forall a,t,i \tag{18}$$

$$NIDC_{a,t,i} = \frac{NIN_{a,t,i}}{POD_i} \forall a,t,i \tag{19}$$

$$NSWC_{a,t,i} = NSG_{a,t,i} PSWC_i \;\forall a,t,i \tag{20}$$

$$NFWC_{a,t,i} = PFWC_i x_{i,a,t} \;\forall a,t,i \tag{21}$$

$$NEC_{a,t,i} = PEC_{i,k} x_{i,a,t} \;\forall a,t,i,k \tag{22}$$

$$NOVC_{a,t,i} = POVC_i x_{i,a,t} \;\forall a,t,i \tag{23}$$

$$NIN_{a,t,i} = PINC_i x_{i,a,t} \;\forall a,t,i \tag{24}$$

Environmental Impact Functions

Park-level

Carbon emission ($TCE_{a,t}$), sewage output ($TWW_{a,t}$), solid waste output ($TSG_{a,t}$), sewage infrastructure capital investment ($TWWI_{a,t}$), sewage infrastructure annual operation cost ($TWWAC_{a,t}$), and sewage infrastructure annual electricity consumption ($TWWE_{a,t}$) are considered in environmental impact functions in park-level. The relevant equations for environmental impact functions are shown in Equation (25) to (30).

$$TCE_{a,t} = \sum_{m} CE_{a,t,m} \ \forall a,t \tag{25}$$

$$TWW_{a,t} = \sum_{m} WW_{a,t,m} \ \forall a,t \tag{26}$$

$$TSG_{a,t} = \sum_{m} SG_{a,t,m} \ \forall a,t \tag{27}$$

$$TWWI_{a,t} = \sum_{m} WWI_{a,t,m} \ \forall a,t \tag{28}$$

$$TWWAC_{a,t} = \sum_{m} WWAC_{a,t,m} \ \forall a,t \tag{29}$$

$$TWWE_{a,t} = \sum_{m} WWE_{a,t,m} \ \forall a,t \tag{30}$$

Industry-level

Six environmental impact functions ($CE_{a,t,m}, WW_{a,t,m}$, $SG_{a,t,m}$, $WWI_{a,t,m}, WWAC_{a,t,m}$, $WWE_{a,t,m}$) are selected to describe the environmental cost of different industry (SLI - saline lake industry, OCI - organic chemical industry, IOCI - inorganic chemical industry, MSPI - metal smelting and processing industry, LDPI - lithium deep processing industry, and CWUI - comprehensive waste utilization industry), the equations are shown in Equation (31) to (36).

$$CE_{a,t,m} = \sum_{i} NCE_{a,t,i} \ \forall a,t,i \in Node_{i,m} \tag{31}$$

$$WW_{a,t,m} = \sum_{i} NWW_{a,t,i} \ \forall a,t,i \in Node_{i,m} \tag{32}$$

$$SG_{a,t,m} = \sum_{i} NSG_{a,t,i} \ \forall a,t,i \in Node_{i,m} \tag{33}$$

$$WWI_{a,t,m} = WW_{a,t,m} PWWI \ \forall a,t,i \in Node_{i,m} \tag{34}$$

$$WWAC_{a,t,m} = WW_{a,t,m} PWWA \ \forall a,t,i \in Node_{i,m} \tag{35}$$

$$WWE_{a,t,m} = WW_{a,t,m}PWWE \; \forall a,t,i \in Node_{i,m} \tag{36}$$

Node-level

At the node level, the carbon emission ($NCE_{a,t,i}$) for each node is further divided into four categories: (i) the carbon emission due to the electricity consumption of node production process ($NCEE_{a,t,i}$), (ii) the carbon emission due to the steam heating of node production process ($NCES_{a,t,i}$), (iii) the carbon emission due to the direct emission from the production process of node ($NCED_{a,t,i}$), (iv) the carbon emission due to fuels consumption for the production process of node ($NCEO_{a,t,i}$). The emission factors of each energy consumption are considered in the calculation. Sewage output ($NWW_{a,t,i}$), solid waste output ($NSG_{a,t,i}$) are two functions to describe the environmental impact of nodes. Equations are shown in Equation (37) to (43).

$$NCE_{a,t,i} = NCEE_{a,t,i} + NCES_{a,t,i} + NCED_{a,t,i} + NCEO_{a,t,i} \; \forall a,i,t \tag{37}$$

$$NCEE_{a,t,i} = PEEC_i x_{i,a,t} PCC_{electricity} \; \forall a,i,t \tag{38}$$

$$NCES_{a,t,i} = PEC_{i,steam} x_{i,a,t} PCC_{steam} \; \forall a,i,t \tag{39}$$

$$NCED_{a,t,i} = Outreso_{i,CO_2} x_{i,a,t} \; \forall a,i,t \tag{40}$$

$$NCEO_{a,t,i} = PEC_{i,k,k \neq steam} x_{i,a,t} PCC_{k,k \neq steam} \; \forall a,i,t \tag{41}$$

$$NWW_{a,t,i} = PWCF_i PWC_i x_{i,a,t} \; \forall a,i,t \tag{42}$$

$$NSG_{a,t,i} = PSWO_i x_{i,a,t} \; \forall a,i,t \tag{43}$$

<u>*Resources Consumption Functions*</u>

Park-level

Freshwater consumption ($TWC_{a,t}$), energy consumption ($TEN_{a,t}$, converted to tce), and electricity consumption ($TEC_{a,t}$) are included in the resources consumption functions at the park

level for the measure of industrial park resources consumption, the related equations are shown in Equation (44) to (46).

$$TWC_{a,t} = \sum_{m} WC_{a,t,m} \ \forall a,t \tag{44}$$

$$TEN_{a,t} = \sum_{m} EN_{a,t,m} \ \forall a,t \tag{45}$$

$$TEC_{a,t} = \sum_{m} EC_{a,t,m} \ \forall a,t \tag{46}$$

Industry-level

At the industry level, there are three functions to describe the industry resources consumption, namely, freshwater consumption ($WC_{a,t,m}$), energy consumption ($EN_{a,t,m}$), and electricity consumption ($EC_{a,t,m}$), the related equations are shown in Equation (47) to (49).

$$WC_{a,t,m} = \sum_{i} NWC_{a,t,i} \ \forall a,t,i \in Node_{i,m} \tag{47}$$

$$EN_{a,t,m} = \sum_{i} NEN_{a,t,i} \ \forall a,t,i \in Node_{i,m} \tag{48}$$

$$EC_{a,t,m} = \sum_{i} NEC_{a,t,i} \ \forall a,t,i \in Node_{i,m} \tag{49}$$

Node-level

At the node level, there are three functions to measure the nodes' resource consumption degree, namely, freshwater consumption ($NWC_{a,t,i}$), energy consumption ($NEN_{a,t,i}$), and electricity consumption ($NEC_{a,t,i}$). The relevant equations are Equation (50) to (52).

$$NWC_{a,t,i} = \sum_{i} PWC_i \, x_{i,a,t} \ \forall a,t \tag{50}$$

$$NEN_{a,t,i} = \sum_{i} \sum_{k} PEC_{i,k} \, x_{i,a,t} \ \forall a,t \tag{51}$$

$$NEC_{a,t,i} = \sum_{i} PEEC_i \, x_{i,a,t} \ \forall a,t \tag{52}$$

<u>Intensity and Productivity Functions</u>

The intensity of resource consumption measures the amount of resources consumed per unit of economic output, and through these functions, the relationship between resource consumption and economic development at the park level can be traced, while constraints can be placed on the intensity of a given resource consumption. The functions of productivity can measure the economic output per unit of resource consumption and can measure the degree of optimal allocation of resources in the industrial network. The intensity/productivity of freshwater, solid waste, carbon emission, electricity, and energy are selected. The equations are Equation (53) to (66).

Park-level

$$TELI_{a,t} = TEC_{a,t}/TGV_{a,t} \ \forall a,t \tag{53}$$

$$TENI_{a,t} = TEN_{a,t}/TGV_{a,t} \ \forall a,t \tag{54}$$

$$TSI_{a,t} = TSG_{a,t}/TGV_{a,t} \ \forall a,t \tag{55}$$

$$TWI_{a,t} = TWC_{a,t}/TGV_{a,t} \ \forall a,t \tag{56}$$

$$TEP_{a,t} = TGV_{a,t}/TEN_{a,t} \ \forall a,t \tag{57}$$

$$TWP_{a,t} = TGV_{a,t}/TWC_{a,t} \ \forall a,t \tag{58}$$

$$TCI_{a,t} = TCE_{a,t}/TGV_{a,t} \ \forall a,t \tag{59}$$

Industry-level

$$ELI_{a,t,m} = EC_{a,t,m}/GV_{a,t,m} \ \forall a,t,i \in Node_{i,m} \tag{60}$$

$$ENI_{a,t,m} = EN_{a,t,m}/GV_{a,t,m} \ \forall a,t,i \in Node_{i,m} \tag{61}$$

$$SI_{a,t,m} = SG_{a,t,m}/GV_{a,t,m} \ \forall a,t,i \in Node_{i,m} \tag{62}$$

$$WI_{a,t,m} = WC_{a,t,m}/GV_{a,t,m} \ \forall a,t,i \in Node_{i,m} \tag{63}$$

$$EP_{a,t,m} = GV_{a,t,m}/EN_{a,t,m} \ \forall a,t,i \in Node_{i,m} \tag{64}$$

$$WP_{a,t,m} = GV_{a,t,m}/WC_{a,t,m} \ \forall a,t,i \in Node_{i,m} \tag{65}$$

$$CI_{a,t,m} = CE_{a,t,m}/GV_{a,t,m} \ \forall a,t,i \in Node_{i,m} \tag{66}$$

Objective Function

The model is solved to minimize the intensity functions and maximize the economic output, which is a multi-objective optimization problem. For this purpose, the objective function is designed as Equation (67) to (71).

$$U_{TGV_{a,t}} = \frac{TGV_{a,t} - TGV_{a,t}^{min}}{TGV_{a,t}^{max} - TGV_{a,t}^{min}} \ \forall \mathrm{a,t} \tag{67}$$

$$U_{TWI_{a,t}} = \frac{TWI_{a,t}^{max} - TWI_{a,t}}{TWI_{a,t}^{max} - TWI_{a,t}^{min}} \ \forall \mathrm{a,t} \tag{68}$$

$$U_{TENI_{a,t}} = \frac{TENI_{a,t}^{max} - TENI_{a,t}}{TENI_{a,t}^{max} - TENI_{a,t}^{min}} \ \forall \mathrm{a,t} \tag{69}$$

$$U_{TSI_{a,t}} = \frac{TSI_{a,t}^{max} - TSI_{a,t}}{TSI_{a,t}^{max} - TSI_{a,t}^{min}} \ \forall \mathrm{a,t} \tag{70}$$

$$U = U_{TGV_{a,t}} + U_{TWI_{a,t}} + U_{TENI_{a,t}} + U_{TSI_{a,t}} \ \forall \mathrm{a,t} \tag{71}$$

The resulting optimization problem is:

$$\max \ U \ (Equation\ 71) \tag{72}$$

$$\mathrm{s.t.} \ Equations\ (1) - (70) \tag{73}$$

Transition description

The RRIEDOM model plays a pivotal role in steering the industrial zone toward achieving its economic development targets. This entails a gradual reduction in the minimum technological scale within the existing industrial structure under each development objective. The model is instrumental in making decisions regarding the retention of relevant technologies within the original industrial framework, essentially establishing the constrained optimal path aligned with the present economic

development goals.

When ascertaining the optimal transition path, careful consideration is given to both the original structure and the optimal industrial configuration determined through optimization. Additionally, the time-dependent minimum scale requirements for transitioning between these two structures are factored in.

In summary, the process involves an initial examination of the original industrial structure, followed by optimization of the optimal industrial arrangement, culminating in the simulation of the transition path.

Determining the Transition Steps from the Original to the Optimal Industrial Structure

Investigation of the original structure: The first step involves comprehensive research into the existing industrial framework of the industrial zone. This entails field research, enterprise inspections, government consultations, and expert input. The collected data encompasses details on project types, scales, pollutant profiles, and resource development aspects. Once this information is compiled, it is used to verify the fundamental characteristics of the industrial zone's original structure through the application of the RRIEDOM model.

Identification of the optimal industrial structure: Building upon the data of resource endowment and capital investment within the industrial zone, the optimal industrial configuration is determined through optimization using the RRIEDOM model.

Industrial structure transition: Within the context of the original industrial structure, a defined transition period is set, typically spanning 5, 10, or 20 years. For example, a 10-year transition period might be selected. During the initial stages of this period, there is a gradual reduction in the minimum technical scale constraints associated with the original structure. This strategic reduction

in constraints allows for greater flexibility and decision-making latitude within the RRIEDOM model. Over time, the minimum technical scale of the original industrial structure is progressively diminished until it aligns with the optimal industrial structure.

Under the constraint of equal step changes in economic targets, denoted as $TGV_{a,t} = Expectoutput$, and with regard to technological scale constraints, we systematically relax the lower constraints on $x_i \in Original\ structure$ from $PLBN_{a,t,i}$ to 0. This relaxation occurs over a transition period of 5 years, ensuring that $PLBN_{a,t+5,i} = 0$, then further extends to 10 years, guaranteeing $PLBN_{a,t+10,i} = 0$, and ultimately extends to a 20-year transition period where $PLBN_{a,t+20,i} = 0$ is maintained. This approach aligns to manage these constraints in a phased manner, allowing for a smoother transition and ensuring that the model reaches the desired state within the specified timeframes.

Detailed Clarification

Defining the optimal industrial structure: The optimal industrial structure represents a scenario where, under specified conditions encompassing resource endowment, capital investment, material constraints, price information, and investment depreciation, a solution emerges. It is characterized by a unique configuration that optimally satisfies these conditions.

Model-based simulation process: The model's simulation procedure relies on predefined time settings for relevant parameters. This involves a gradual increment in capital investment, encompassing aspects like resource endowment, capital investment, material constraints, price information, and investment depreciation until the system properties align with those stipulated under the optimal industrial structure.

Supplementary Method 4 Method of the construction of eco-industrial network

<u>*Construction Order*</u>

This study is oriented to the Qinghai industrial park, where the industrial system is built on a large scale, involving a large number of resources and energy, processes, and input and output materials, so there will be massive material combinations of the material group, and the amount of operations for interactive inference with the node information database is huge. In this study, the idea of building each industrial chain step by step in a certain order and finally forming an industrial network is to reduce the complexity of construction: (i) only the information database of the industrial node needs to be called when building a certain industrial chain, thus reducing the amount of reasoning in the interactive process and distributing the inference amount to each round of construction; (ii) the material groups are also reduced before the interactive inference, i.e., the materials in the initial material group that do not appear in the material group of the target industry node information base are removed, thus reducing the invalid material combinations in the interaction process.

The saline lake industry (SLI) directly uses the main resources (saline brine, etc.), and the nodes involved in its industry produce a large number of basic chemicals (such as sodium hydroxide, hydrochloric acid, etc.), so it is in a basic position in the whole industrial system, and the construction of the industrial system starts with the interactive inference process of the original material group and the information base of the nodes of the saline lake resource industry.

In the first step, the primary material group and SLI node information base are inferred interactively, new nodes are selected, new materials are added to the material group, new material combinations are generated, and new nodes will likely be selected, repeating this process until finally no nodes are selected, then the construction of the basic industry network (i.e., saline lake resource industry network) is completed. After the construction is completed, the output materials of the selected nodes are added to the total material group.

The second step is to select the industry for the next round of construction, call its node information base, use the material information of this node information base to reduce the total material group, remove the materials that do not appear in this node information base, and form a new initial material group, and reason with this material group and this industry node information base until no new node is selected and this round of construction is completed.

In the third step, repeat the second step until the industrial networks of all target industries are built, and then the construction of the whole industrial network is completed.

Material Group

A material group is a collection containing various materials and their basic information that contains numbers (e.g., M1) substance names (e.g., saline lake brine), etc. The number is used to facilitate the composition of material combinations and search to avoid duplicate materials in the material group due to different nomenclature of the same substance (e.g., NaOH and sodium hydroxide), the name of the substance needs to be standardized and unified to achieve accurate search.

There are four types of material groups, namely, primary material group (PM group), initial

material group (IM group), node information base material group (NIBM group), and total material group (TM group). The PM group consists of materials directly produced from the resources and energy available in the target area and used in primary industrial processes. The IM group is directly used in each round of construction to form material combinations, interact with the node information database, and determine the material group of selected nodes. Regardless of the sequence of building each industrial network, the PM group is the IM group of the first round of building until the final overall industrial network. The NIBM group contains all the material information (including raw materials and output materials) incorporated into the node in the node information database of a certain industry. In the interactive inference process, as the nodes are continuously selected, the output materials of the selected nodes will also be added into the material group, and the material group composed of all the materials of the selected nodes and the original materials is the TM group.

Node information database

In this study, a "node" is used to represent a specific industrial process/technology, as shown in Supplementary Figure 3. The input/output of the node contains the raw materials required to produce the product and other materials involved in the industrial process (CO_2, sewage, solid waste, etc) and the substances such as process water, energy supply media such as electricity and steam of both direct and indirect resources interaction.

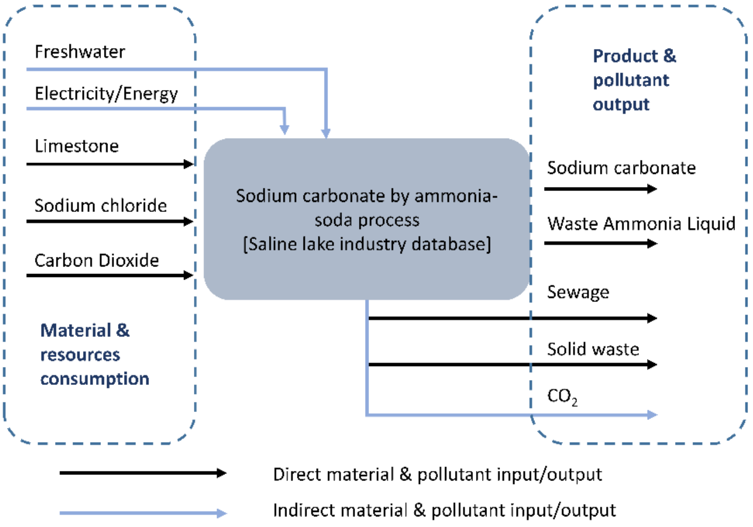

Supplementary Figure 3 Node information schematic diagram of sodium carbonate by ammonia-soda process. Blue lines show the indirect material and pollutant of the input/output of nodes, e.g., energy supply media (electricity or steam). black lines show the direct material and pollutants of nodes, such as limestone, sodium chloride, etc.

The objective is to delineate the industry sectors within the designated geographic region that hold potential for exploitation, and subsequently, to establish a comprehensive database.

The procedural framework encompasses the subsequent steps: (1) Delimiting industry categories, encompassing the salt lake chemical industry, organic chemical industry, inorganic chemical industry, metal processing industry, lithium power industry, and waste treatment industry; (2) Creating the node information databases for each industry; and (3) Populating the database via comprehensive literature review, on-site investigation, and expert interviews.

As exemplified by an extensive series of on-site investigations, a collective sum exceeding 358 nodes has been successfully identified, spanning across 6 distinct industrial categories. Supplementary Figure 4 is a segment of the node information database, specifically numbered 1 to

10, about the saline lake industry.

No.	Node Nomenclature	Industrial type	Input-1	Input-2	Input-3	Input-4	Output-1	Output-2	Output-3
1	N - Saltern to carnallite	Saling lake industry	Brine	Brine			Carnallite	Sodium chloride	Old bittern
2	N - KCl by reverse-current trip flotation	Saling lake industry	Carnallite				Potassium chloride	Old bittern	
3	N - K_2SO_4 by double decomposition	Saling lake industry	Magnesium sulfate	Potassium chloride			Potassium sulfate	Magnesium chloride	
4	N - K_2CO_3 by electrolysis process	Saling lake industry	Potassium chloride	Carbon dioxide	Water		Potassium carbonate	Chlorine	Hydrogen
5	N - KOH	Saling lake industry	Potassium chloride	Water			Potassium hydroxide	Chlorine	Hydrogen
6	N - $KMnO_4$	Saling lake industry	Manganese dioxide	Potassium hydroxide	Water		Potassium permanganate		
7	N - K by substitution method	Saling lake industry	Sodium metal	Potassium chloride			Metal potassium	Sodium chloride	
8	N - $NaNO_3$ by neutralization	Saling lake industry	Nitric acid	Sodium hydroxide			Sodium nitrate	Water	
9	N - KNO_3	Saling lake industry	Sodium nitrate	Potassium chloride			Potassium nitrate	Sodium chloride	
10	N - Chlor-alkali industry	Saling lake industry	Sodium chloride	Water			Sodium hydroxide	chlorine	Hydrogen

Supplementary Figure 4 Examples of node information database. The node information database includes the nomenclature, industry type, major input materials, and major output materials for each technology, and the detailed node information base is provided in Supplementary Data 1.

To build a reasonable industrial network, all currently known advanced processes/technologies should be taken into consideration, so a node information database should include all nodes. Depending on the industries, different industrial node information databases are generated and called when the industrial network construction is carried out step by step. Since the resource endowment and accessible raw materials may be different in different regions, not all nodes in the industry node information database will necessarily be extracted into the industry system by the interactive inference process. To reasonably reduce the number of operations in the interactive reasoning process, the number scale of each industry node information database is constrained with no more than 100 nodes during the construction process. The node information database can be expanded with the continuous accumulation of new technologies. There are 6 node information databases, which are the SLI information database, OCI information database, IOCI information database, MSPI information database, LDPI information database, and CWUI information database.

Interactive inference

The primary objective is to amalgamate data sourced from the node information database to

formulate the network superstructure specific to each respective industry.

The procedure entails the following steps: (1) Identifying and categorizing natural resources; (2) Organizing and amalgamating these identified resources; (3) Correlating nodes; (4) Establishing the network structure, among other pertinent actions.

To elucidate this process, it is supplemented with interactive inference components, such as a logical block diagram and an illustrative reasoning example diagram. In Supplementary Figure 6, this demonstration is conducted briefly utilizing virtual resources labeled A, B, and C, along with virtual nodes designated as 1, 2, and 3. The underlying objective is to ensure the efficiency of the network in harnessing each category of natural resource, thereby minimizing the presence of redundant nodes, while concurrently achieving the intended output targets.

The interactive inference is defined as follows: (i) randomly select several (no more than 4) materials in the IM group to form a material combination and compare the material combination with the input material combinations of all nodes in the industrial node information database, (ii) if the input material combination of the node equals the randomly selected material combination, the node will be selected into the industrial chain network, (iii) add the output materials of all selected nodes into the IM group and new material combinations are generated due to the addition of new materials, (iv) repeat the above comparison, selection and addition process until the material combinations constructed by the final material group can no longer make the nodes in the industrial node information database selected, then the inference process of this round of industrial chain network construction is completed.

The interactive inference of the construction of the industrial chain will continuously carried out until the industrial chains of all target industries are constructed, and all of the industrial chains

will finally form the industrial network. The logic diagram of interactive inference is shown in Supplementary Figure 5 and Supplementary Figure 6.

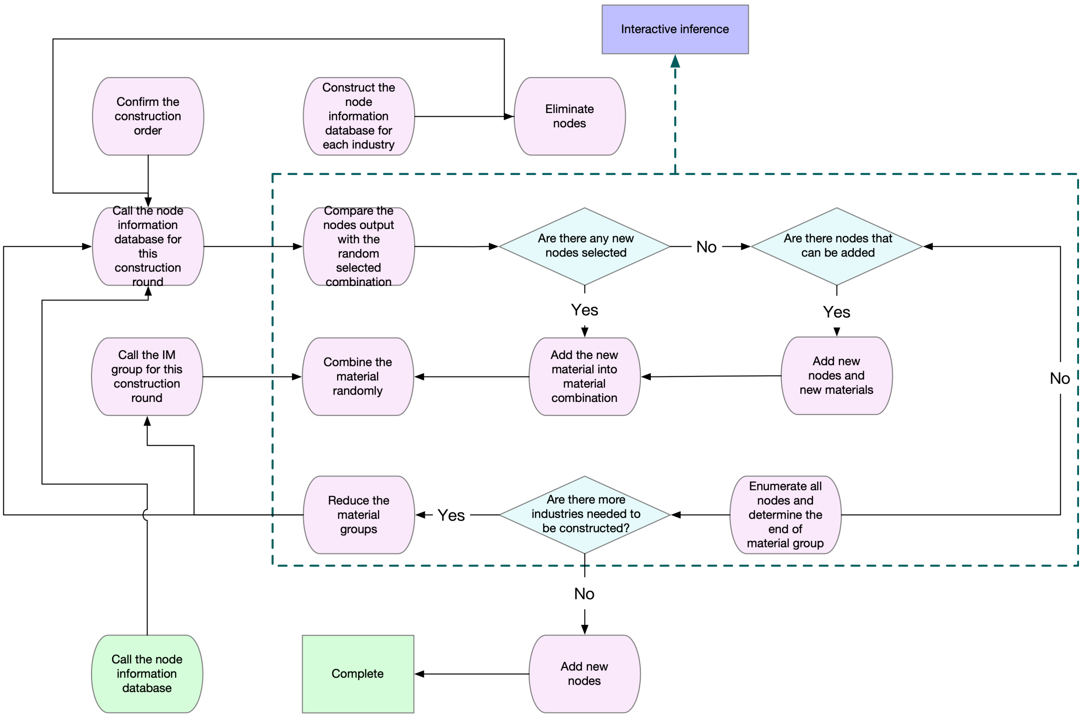

Supplementary Figure 5 Interactive inference logic diagram. The interactive inference method is realized by calling on different node information databases for the construction of industry-specific network superstructures.

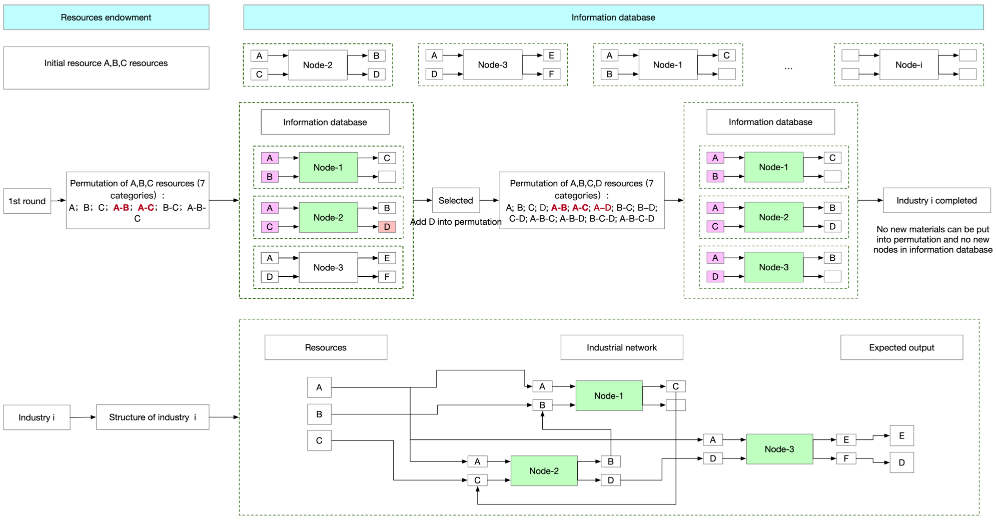

Supplementary Figure 6 Example of interactive inference. Interactive inference determines

whether a technology is suitable to be included in the superstructure of the target industry through the possible combinations of input materials and output materials of different technologies after multiple rounds of construction.

Node information database

The industry node information database stores all the nodes to be selected for several target industries. Nodes are identified based on current industry networks, introduced technologies involved in planning, and emerging technologies with potential in the future. The main content of 6 node information databases is shown as follows.

- SLI information database: the extraction and preparation of potassium, sodium, magnesium, and calcium salts;
- OCI information database: the production of coal chemicals, natural gas chemicals, petrochemical and benzene chemicals, plastic chemicals, and other basic organic chemical products;
- IOCI information database: the production of monomers and compounds of fluorine, phosphorus, boron, silicon, and other elements and new material chemicals;
- MSPI information database: smelting, deep processing, and preparation of related compounds of lead, zinc, strontium, copper, nickel, cobalt, and other metals;
- LDPI information database: lithium extraction from saline brine, preparation of lithium salt, and industrial process of lithium battery;
- CWUI information database: comprehensive utilization of difficult bulk waste generated by the industrial network.

SLI information database

The SLI information database includes 56 nodes, mainly potassium chloride, potassium sulfate, potassium magnesium fertilizer, potassium nitrate, and other potash production, the nodes as shown in Supplementary Table 1.

Supplementary Table 1 The saline lake industry (SLI) information database.

No.	Nomenclature	No.	Nomenclature
1	N - Saltern to carnallite	29	N - Kainite
2	N - KCl by reverse-current trip flotation	30	N - Potash magnesium sulphate fertilizer
3	N - K_2SO_4 by double decomposition	31	N - $KClO_4$
4	N - K_2CO_3 by electrolysis process	32	N - $NaClO_3$
5	N - KOH	33	N - NaCl
6	N - $KMnO_4$	34	N - $C_4H_7NaO_4$ from Na_2CO_3
7	N - K by substitution method	35	N - $Na_2S_2O_3$ from Na_2S
8	N - $NaNO_3$ by neutralization	36	N - $Na_2S_2O_3$ from Na_2SO_3
9	N - KNO_3	37	N - K_2SO_4 from saline lake
10	N - Chlor-alkali industry	38	N - Potassium Oleate
11	N - Production of ADC foaming agent	39	N - $Na_2S_2O_4$ from HCOONa
12	N - PVC by calcium carbide	40	N - $Na_2S_2O_3$ from Na_2CO_3
13	N - HCl	41	N- C_2H_2 from calcium carbide
14	N - Production of calcium carbide	42	N - Compound fertilizer
15	N - Na_2CO_3 by alkaline process	43	N - HCOONa from CO
16	N – Na by electrolysis	44	N - Na_2O_2
17	N - Dehydration of old brine	45	N - Ca by electrolysis
18	N - Mg by electrolysis	46	N - Na_2SO_4 from mirabilite
19	N – Downstream of $MgCO_3$	47	N - Sodium polysulfide
20	N - $Mg(OH)_2$ by NH_3 precipitation	48	N - Na_2CO_3 by ammonia soda process
21	N - Downstream of $Mg(OH)_2$	49	N - $NaNO_3$ by neutralization
22	N - MgO	50	N - $MgCO_3$ by $MgCl_2$
23	N - Fireclay brick by MgO	51	N - $NaNH_2$
24	N - Magnesium alloy and pressure contact-die-casting	52	N - Na_2S
25	N - $MgSO_4$	53	N – Downstream of $CaCl_2$
26	N - Limestone calcination	54	N - $CaCl_2$ from waste ammonia liquid
27	N - CaO	55	N - KNO_3 by neutralization
28	N - Reinforced glass	56	N - Recovery of K and Na from K tail salt

OCI information database

The OCI information database includes 103 nodes, the main processes of OCI are coal-based, mainly N-Coking, N-MTO, and N-Syngas from coal. The OCI nodes as shown in Supplementary Table 2.

Supplementary Table 2 The organic chemical industry (OCI) information database.

No.	Nomenclature	No.	Nomenclature
1	N - Coking	53	N - CH_3OH from coal
2	N - MTO	54	N - NH_3 from coal
3	N - Syngas from coal	55	N - Urea from NH_3
4	N - PVC by oxy-chlorination	56	N - Direct coal liquefaction
5	N - F-T synthesis	57	N - Natural gas from coal
6	N - DME from CH_3OH	58	N - Dimethyl ether from coal
7	N - Thermal cracking of natural gas (1)	59	N - Thermal cracking of natural gas (2)
8	N - Hexamethylenetetramine	60	N - Dimethyl carbonate by methanol carbonyl process
9	N - DMC by direct alcoholysis of urea	61	N - Dimethyl carbonate by indirect urea method
10	N - Oil refining (1)	62	N - Oil refining (2)
11	N - Oil refining (3)	63	N - Oil refining (4)
12	N - Oil refining (5)	64	N - Polypropylene
13	N - CH_3OH to aromatics	65	N - CH_3OH from natural gas
14	N - HNO_3 by ammoxidation	66	N - CPP
15	N - PET synthesis	67	N - PPS from Na_2S
16	N - Polyethylene	68	N - CPE
17	N - Glycol from coal	69	N - Glycol from ethylene oxide
18	N - Ethylene oxide by oxidation	70	N - Ethylene oxide by chlorohydrin method
19	N - Styrene by ethylbenzene dehydrogenation	71	N - Paraxylene by toluene disproportionation method
20	N - P-phthalic acid	72	N - Methylmercaptan
21	N - Methionine	73	N - Lead stearate
22	N - Calcium stearate	74	N - Dioctyl phthalate
23	N - Dioctyl sebacate	75	N - Cadmium stearate
24	N - Triphenyl phosphite	76	N - Barium stearate
25	N - HCN by light oil cracking method	77	N - HCN from CH_4 and NH_3
26	N - Acrylonitrile by propylene ammoxidation	78	N - Acetonitrile from NH_3 and CH_3COOH
27	N - Acetonitrile from NH_3 and C_3H_8	79	N - Acetonitrile from C_3H_8
28	N - NaCN from HCN	80	N - Acetone cyanohydrin
29	N - Methacrylate by acetone cyanohydrin method	81	N - Methyl methacrylate from C_3H_6
30	N - Polymethacrylate	82	N - CH_3COOH from CH_3OH
31	N - Acetone by C_3H_6	83	N - Acetone and phenol
32	N - Bisphenol A	84	N - Epoxy resin

33	N - Epichlorohydrin from C_3H_6	85	N - Epichlorohydrin by acetic acid propylene ester method
34	N - Epichlorohydrin from glycerol	86	N - Nitrochlorobenzene
35	N - Nitrophend	87	N - Nitrobenzene
36	N - Orlon	88	N - Acrylamide
37	N - ABS	89	N - PAM
38	N - Ethylbenzene by benzene alkylation	90	N - Formaldehyde
39	N - Polystyrene	91	N - Styrene butadiene rubber
40	N - Aniline by catalytic hydrogenation of nitrobenzen	92	N - Isocyanate
41	N - Polyurethane	93	N - PVC from C_3H_6
42	N - Rubber absorber antioxidant	94	N - Downstream of nitrochlorobenzene
43	N - CPVC	95	N - UPVC
44	N - Pipe CPVC	96	N - CPVC pipe
45	N - Plate CPVC	97	N - PVC plate
46	N - Speciality resin	98	N - PVC wire
47	N - PVC plastic doors and windows	99	N - Chloromethane
48	N - Methyl acetate from CH_3OH	100	N - Ethyl alcohol from C_2H_4
49	N - CH_3CH_2OH from CH_3COOH	101	N - Ethyl alcohol from CH_3COOH
50	N - Diethyl carbonate	102	N - Ethyl methyl carbonate by ester exchange method
51	N - Ethylene carbonate	103	N - Ethylene carbonate ethylene oxide
52	N - Propylene carbonate		

IOCI information database

The IOCI information database includes 88 nodes. The IOCI nodes as shown in Supplementary Table 3.

Supplementary Table 3 The inorganic chemical industry (IOCI) information database.

No.	Nomenclature	No.	Nomenclature
1	N - HF from fluorspar	45	N - Hydrofluoric acid from HF
2	N - Electronic grade HF	46	N - NF_3 by synthesis
3	N - NF_3 by electrolysis	47	N - NaF from Na_2SiF_6
4	N - KF by neutralization	48	N - KF from K_2SiF_6
5	N - NH_4F from NH_3 and HF	49	N - NH_4HF_2 from H_2SiF_6
6	N - Acidolysis of silicate	50	N - H_2SiF_6 by SiF_4 hydrolysis
7	N - SiF_4 from Na_2SiF_6	51	N - SiF_4 from fluorspar
8	N - Na_2SiF_6	52	N - UF_4
9	N - UF_6	53	N - F_2 by electrolysis
10	N - KHF_2 from KOH	54	N - H_3PO_4 by wet process

11	N - $Na_5P_3O_{10}$	55	N - $CaHPO_4$ from $CaCl_2$
12	N - $CaHPO_4$ from $CaCO_3$	56	N - NaH_2PO_4
13	N - $(NaPO_3)_6$	57	N - Na_3PO_4
14	N - Tributyl phosphate	58	N - H_3PO_3
15	N - KH_2PO_4 from K_2CO_3	59	N - KH_2PO_4 from KOH
16	N - $Na_2HPO_3 \cdot 5H_2O$	60	N - $(NH_4)_3PO_4$
17	N - Ammonium polyphosphate from $(NH_4)_3PO_4$	61	N - Ammonium polyphosphate from H_3PO_4
18	N - Ammonium polyphosphate from H_3PO_4	62	N - BPO_4
19	N - PF_5 from HF	63	N - PF_5 from CaF_2
20	N - PCl_3	64	N - PCl_5
21	N - P	65	N - $FePO_4$
22	N - S	66	N - H_3BO_3 from ludwigite and HCl
23	N - H_3BO_3 from ludwigite	67	N - H_3BO_3 from saline brine
24	N - B_2O_3	68	N - B
25	N - BF_3 from B_2O_3	69	N - BF_3 by pyrolytic process
26	N - 10B acid	70	N - 11B acid
27	N - B_4C	71	N - BN from borax and NH_4Cl
28	N - Boronnitride	72	N - BCl_3
29	N - KBH_4	73	N - $NaBH_4$ by schlesinger method
30	N - $NaBH_4$	74	N - KBF_4 from HF
31	N - KBF_4	75	N - $LiBF_4$
32	N - Borax	76	N - Borax by alkali carbonate method
33	N - Si for industry	77	N - Polysilicon by siemens method
34	N - Monocrystalline Si	78	N - Quartz sand
35	N - Silicon materials	79	N - Optical fiber
36	N - Silica gel	80	N - Organosilicon
37	N - Downstream of organosilicon	81	N - Silane
38	N - $Na_2O \cdot nSiO_2$	82	N - Glass
39	N - SiO_2	83	N - Silica white by precipitation
40	N - Silica white by gas phase method	84	N - Black silicon carbide
41	N - Green silicon carbide	85	N - Crystal by hydrothermal synthesis
42	N - Glimmer	86	N - Quartz product
43	N - Downstream of quartz	87	N - Glass microspheres
44	N - Silicocalcium	88	N - Ferrosilicon

MSPI information database

The MSPI information database includes 48 nodes. The MSPI nodes as shown in

Supplementary Table 4.

Supplementary Table 4 The metal smelting and processing industry (MSPI) information database.

No.	Nomenclature	No.	Nomenclature
1	N - $SrCO_3$ by carbon reduction	25	N - $SrCO_3$ by double decomposition
2	N - $Sr(NO_3)_2$	26	N - $Sr(OH)_2$
3	N - SrO_2	27	N - SrO
4	N - $SrCl_2$	28	N - $SrCrO_4$ from $SrCl_2$
5	N - $SrCrO_4$	29	N - $SrTiO_3$
6	N - Strontium ranelate	30	N - Sr
7	N - Lead-zinc ore dressing	31	N - Pb
8	N - Zinc leaching	32	N - Lead accumulator
9	N - Cable sheath	33	N - Pb profile
10	N - PbO	34	N - Lead alloy
11	N - Pb compound	35	N - PbO_2
12	N - PbO_2 by thermal oxidation method	36	N - Zinc alloy
13	N - Hot-galvanizing	37	N - Brass
14	N - ZnO	38	N - Zn profile
15	N - Brass profile	39	N - Beneficiation of nickel cobalt ore
16	N - Ni	40	N - Low grade Ni matte by blowing
17	N - High grade Ni matte by grinding-flotation	41	N - Electrolysis of nickel concentrate
18	N - Copper concentrate electrolysis	42	N - $NiSO_4$
19	N - $Ni(OH)_2$	43	N - $Ni(CO)_4$
20	N - Co_3O_4	44	N - ZnO from waste bearing zinc
21	N - Al_2O_3	45	N - Al by electrolysis
22	N - Al profile	46	N - Copper pyrometallurgy
23	N - Hydrometallurgical Cu refining	47	N - H_2SO_4
24	N - $AlCl_3$	48	N - AlF_3

LDPI information database

The LDPI information database includes 40 nodes. The LDPI nodes as shown in Supplementary Table 5.

Supplementary Table 5 The lithium deep processing industry (LDPI) information database.

No.	Nomenclature	No.	Nomenclature
1	N - Li_2CO_3 from saline brine	21	N - LiOH

2	N - LiCl	22	N - $LiClO_4$
3	N - CH_3COOLi	23	N - Li_2SO_4
4	N - LiBr	24	N - LiI from LiOH
5	N - LiI from Li_2CO_3	25	N - LiF from LiOH
6	N - LiF from Li_2CO_3	26	N - $LiNO_3$ from LiOH
7	N - $LiNO_3$ from Li_2CO_3	27	N - Li_3PO_4 from LiOH
8	N - Li_3PO_4 from Li_2CO_3	28	N - Li and Al alloy
9	N - Li and Mg alloy	29	N - LiH_2PO_4
10	N - LiO_3P	30	N - Li
11	N - Lithium foil	31	N - LiH
12	N - $LiAlH_4$	32	N - Downstream of Li+
13	N - $Li_4Ti_5O_{12}$	33	N - $LiFePO_4$
14	N - NMC	34	N - $LiNiO_2$
15	N - $LiMn_2O_4$	35	N - Lithium nickel cobalt aluminum oxide
16	N - $LiCoO_2$	36	N - $LiPF_6$ from ion exchange method
17	N - $LiPF_6$ from PF_5	37	N - C_4BLiO_8
18	N - $LiBF_4$ from BF_3	38	N - Cathode material of lithium ion batteries
19	N - Anode materials for lithium-ion batteries	39	N - Li-ion battery separator material
20	N - Lithium-ion battery electrolyte	40	N - Lithium ion battery

CWUI information database

The CWUI information database includes 23 nodes. The CWUI nodes as shown in Supplementary Table 6.

Supplementary Table 6 The comprehensive waste utilization industry (CWUI) information database.

No.	Nomenclature	No.	Nomenclature
1	N - Dinas bricks	13	N - Geomembrane
2	N - Concrete	14	N - Magnesium oxychloride cement
3	N - PE tube	15	N - Cement from coalgangue
4	N - Pyrites from coalgangue	16	N - Molecular sieve from coalgangue
5	N - Baking-free bricks from coalgangue	17	N - $AlCl_3$ from coalgangue
6	N - Manure from coalgangue	18	N - $Ca(OH)_2$ from carbide slag
7	N - $Al_2(SO_4)_3$ from coalgangue	19	N - Epoxyethane
8	N - $CaCO_3$ from carbide slag	20	N - Cement from carbide slag
9	N - K_2SO_4 by IMI method	21	N - Molten salt

10	N - Extraction of Rb and Cs	22	N - Refine of Rb
11	N - Refine of Cs	23	N - Fly ash disposal
12	N - Aerated block		

The superstructure of industrial network

The initial resource conditions for the construction of the industrial network superstructure are set as follows: saline lake brine, coal, natural gas, petroleum, natural sulfur, mannite, limestone, fluorite, silica, column boron magnesite, phosphate ore, graphite, feldspar, azurite, lead-zinc ore, nickel-cobalt ore, bauxite, copper ore, and iron ore.

1st round construction: call SLI information database. After 11 times interactive inferences, there are 55 nodes selected into the superstructure and 72 resources added to the material group. The superstructure of SLI is shown in Supplementary Figure 7.

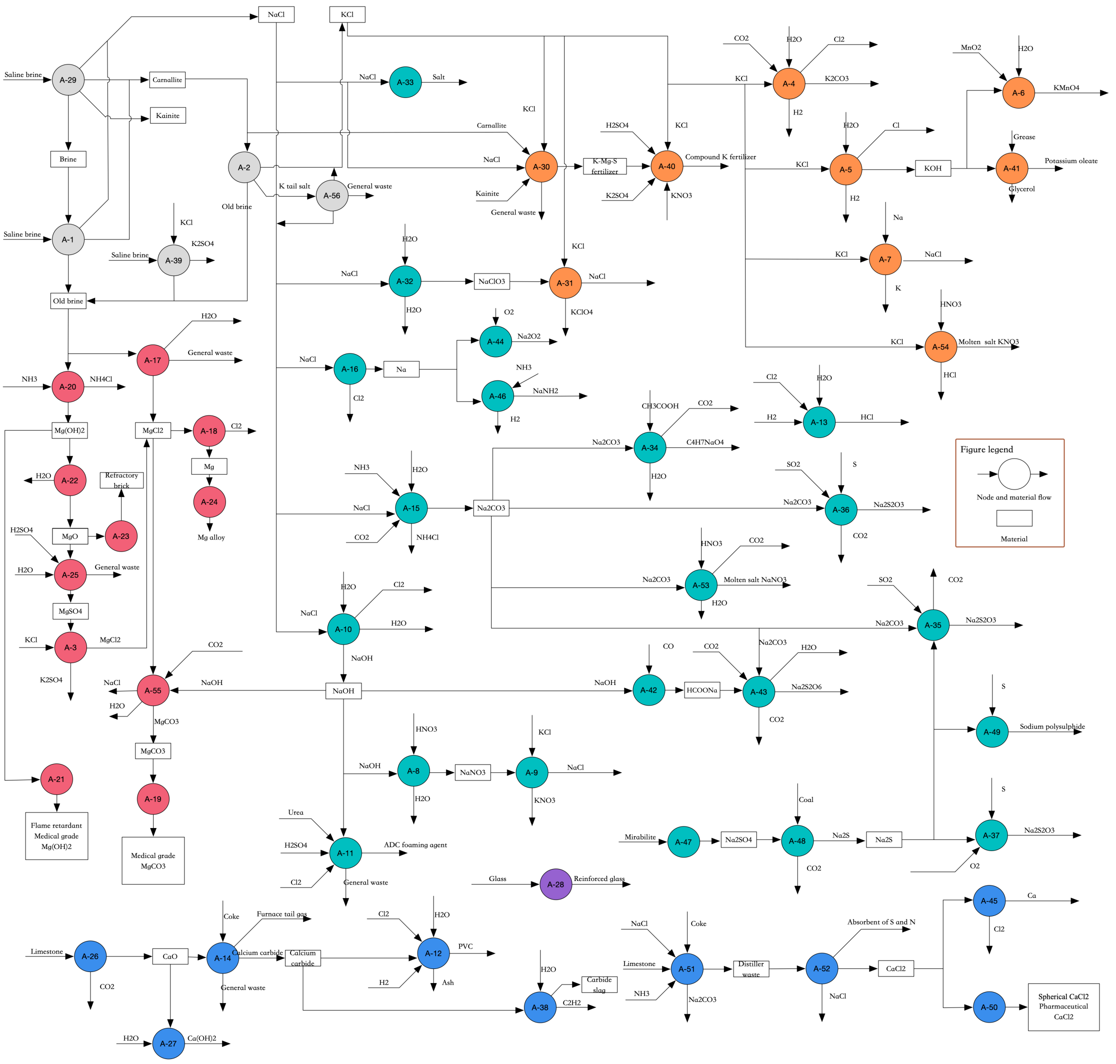

Supplementary Figure 7 The saline lake industry (SLI) superstructure. The saline lake industry includes the utilization of salt lake resources and includes 55 technologies and 72 materials related to the salt lake chemical industry.

2st round construction: call OCI information database. After 10 times interactive inferences, there are 90 nodes selected into the superstructure and 105 resources added to the material group. The superstructure of OCI is shown in Supplementary Figure 8.

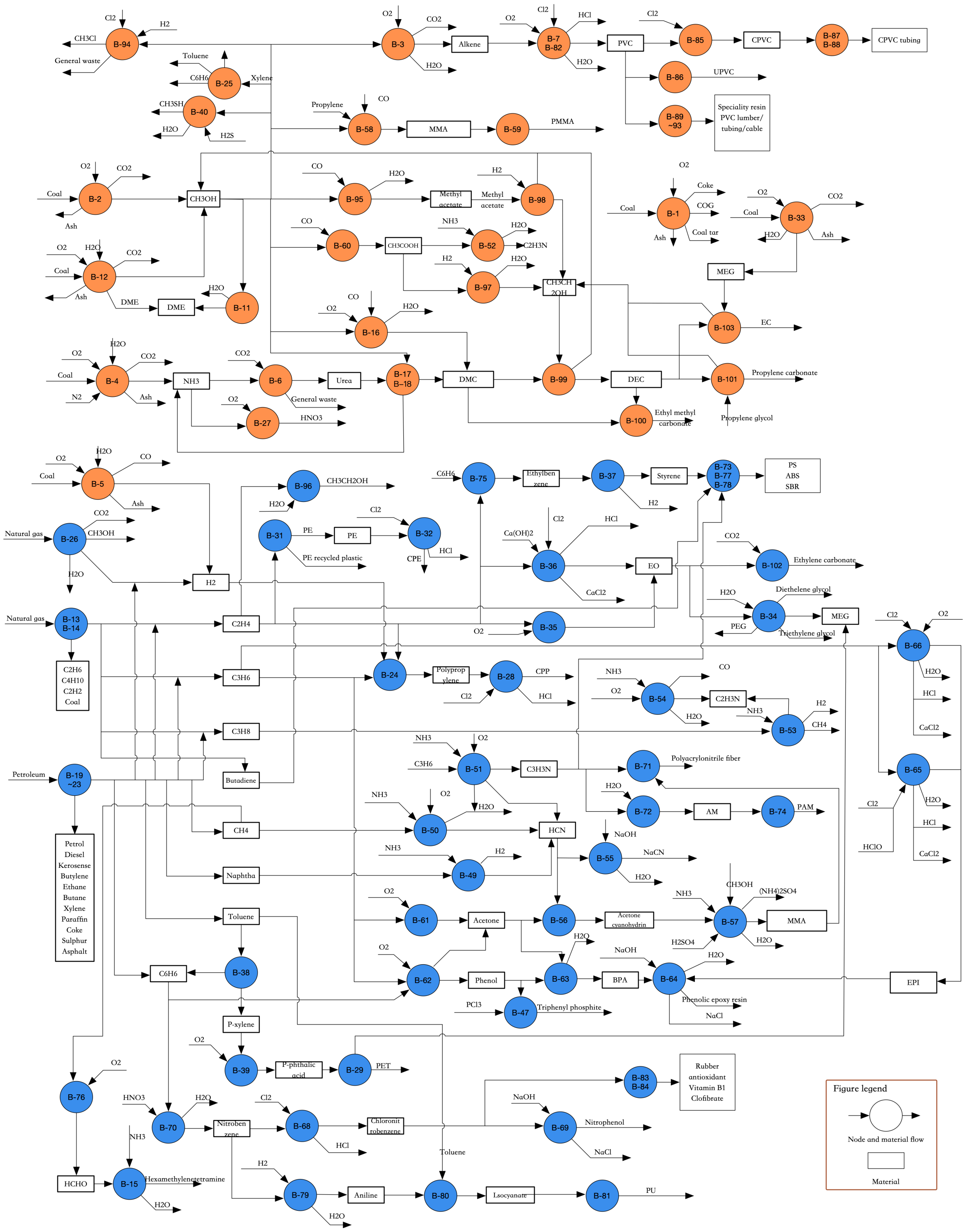

Supplementary Figure 8 The organic chemical industry (OCI) superstructure. The organic chemical industry includes the comprehensive utilization of coal, oil, natural gas, and other resources, involving a total of 90 organic chemical technologies and 105 related materials.

3rd round construction: call IOCI information database. After 8 times interactive inferences, there are 77 nodes selected into the superstructure and 81 resources added to the material group.

The superstructure of IOCI is shown in Supplementary Figure 9.

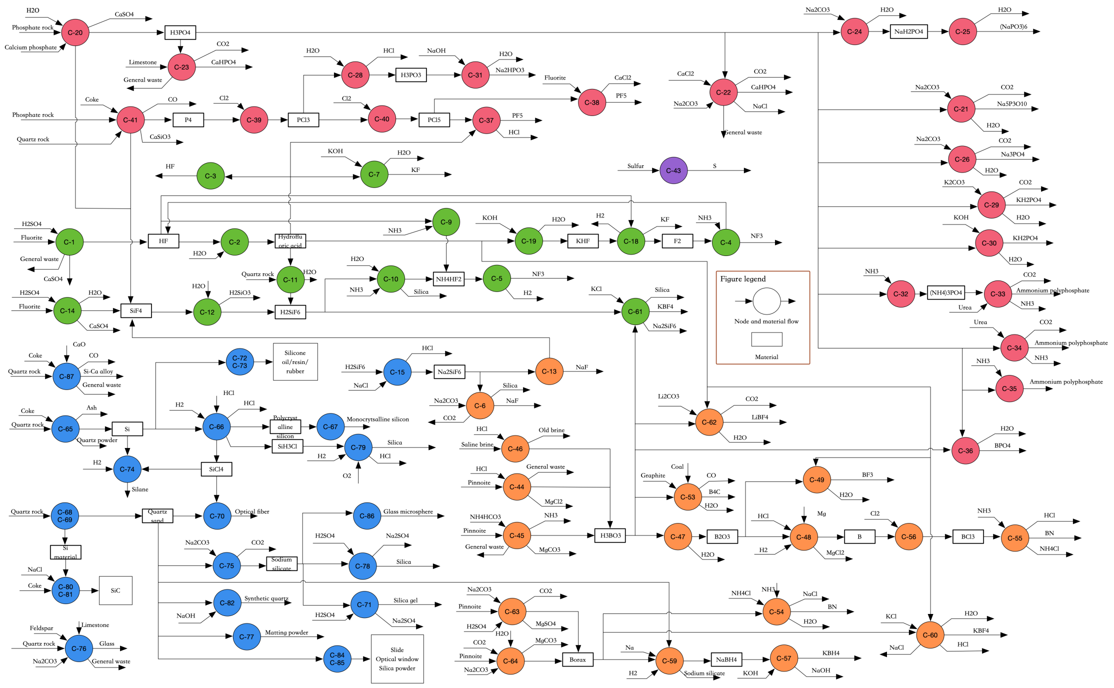

Supplementary Figure 9 The inorganic chemical industry (IOCI) superstructure. The inorganic chemical industry includes the comprehensive utilization of fluorine-containing, silica-containing, and other ore resources, and contains a total of 77 inorganic chemical technologies and the corresponding 81 kinds of materials.

4st round construction: call MSPI information database. After 8 times interactive inferences, there are 40 nodes selected into the superstructure and 54 resources added to the material group. The superstructure of MSPI is shown in Supplementary Figure 10.

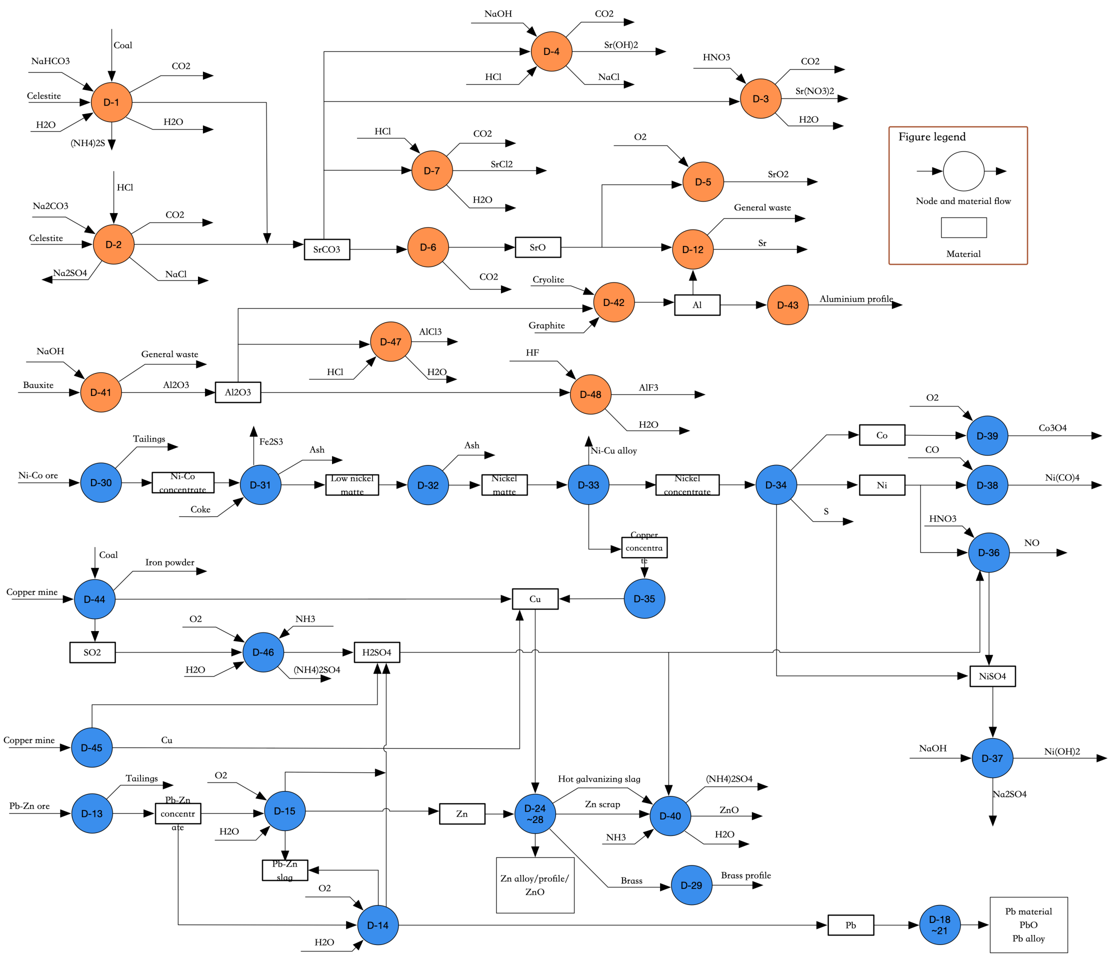

Supplementary Figure 10 The metal smelting and processing industry (MSPI) superstructure.

The metal smelting and processing industry includes the utilization of ores containing copper, aluminum, and nickel, and involves a total of 40 metal smelting and processing technologies and 54 related materials.

5st round construction: call LDPI information database. After 7 times interactive inferences, there are 36 nodes selected into the superstructure and 39 resources added to the material group. The superstructure of LDPI is shown in Supplementary Figure 11.

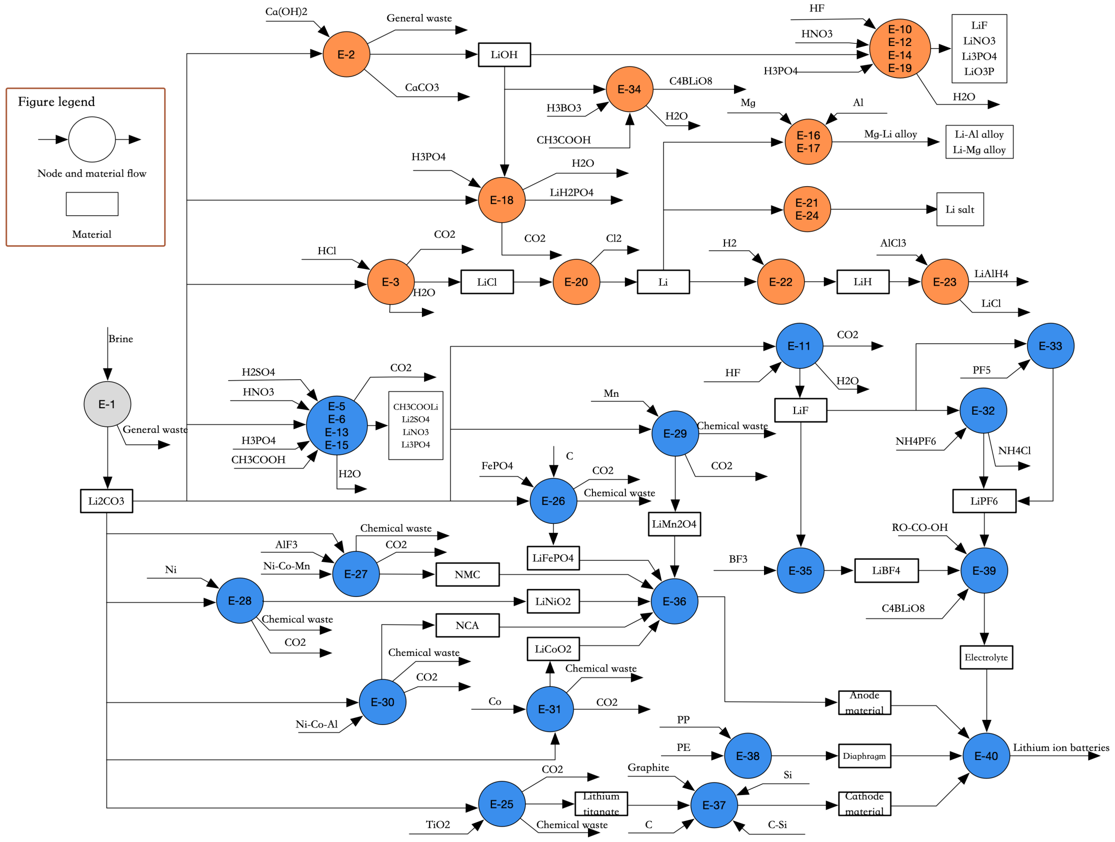

Supplementary Figure 11 The lithium deep processing industry (LDPI) superstructure. Lithium deep processing industry includes the processing and utilization of lithium brine, lithium ore and other resources, including a total of 36 kinds of lithium resource processing technology and 39 kinds of related materials.

6st round construction: call CWUI information database. After 3 times interactive inferences, there are 22 nodes selected into the superstructure and 30 resources added to the material group. The superstructure of CWUI is shown in Supplementary Figure 12.

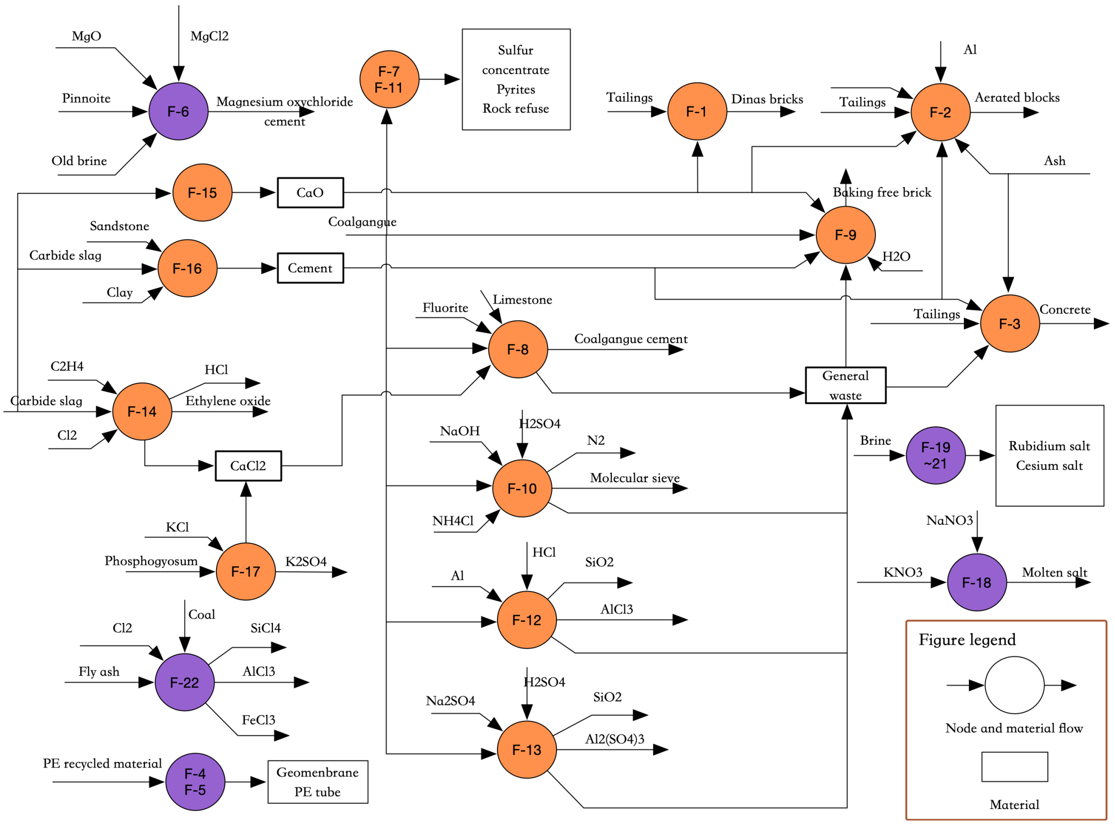

Supplementary Figure 12 The comprehensive waste utilization industry (CWUI) superstructure. The comprehensive utilization of waste industry includes the comprehensive utilization of various types of waste technology, involving a total of 22 types of waste utilization technology and 30 types of related materials.

After six rounds of the construction of the industrial network, the superstructure network of 322 nodes is finally obtained. Six industries (SLI, OCI, IOCI, MSPI, LDPI, and CWUI) are numbered as A, B, C, D, E, F, respectively.

The superstructure of each industry shows the input materials and the output products/by-products of each node and the connection of different nodes, the direct output of pollutants (e.g. solid waste, sewage), and indirect consumption (e.g. electricity, energy, and water consumption) are neglected in the visualization of industrial superstructure for simplicity. The connection of nodes represents the symbiosis that there are materials of the source nodes provided for the target nodes.

The construction of core superstructure

The superstructure of each industry describes all the possible processes and industry chains of the utilization of initial resources (e.g. saline lake brine, coal, natural gas, petroleum) which are transformed into products/by-products of higher economic value. Only the core network needs to be included in the optimization, the core network is the network constituted by the core nodes that are the nodes with higher betweenness centrality (BC) and degree centrality (DC) in the network. The node with higher betweenness centrality as well as degree centrality is in the core position in the network. To identify the core nodes in the network and consider the possible future cascading failures when designing the network, the social network analysis (SNA) method is introduced to design the network's core superstructure.

The degree centrality represents the number of nodes linking to other nodes, more specifically, in this study, it is the number of links of nodes acquiring materials from other nodes, the number of links delivering materials to others. The equation of degree centrality is shown in Equation (74).

$$DC_i = \frac{\deg(i)}{N-1} \tag{74}$$

where deg(i) is the degree of node i, i.e., the number of edges directly connected to node i, and N is the total number of nodes in the network.

The node with high betweenness centrality measures the degree of the node's control over the core link utilization, and the node with high betweenness centrality fails in one point of operation, which will affect the operation of multiple industrial links and may generate cascading failures. The equation of betweenness centrality (BC) is shown in Equation (75).

$$BC_i = \sum_{i \neq s \neq t} \frac{\sigma_{st}(i)}{\sigma_{st}} \tag{75}$$

where i is a node in the network, σ_{st} is the number of shortest paths from node s to node t,

and $\sigma_{st}(i)$ is the number of paths passing through node i in these shortest paths.

According to the node material exchange in each industrial network, the adjacency matrix of each network is determined, and the network topology structure and the DC and BC of each node are obtained in combination with UCINET 6.0, the topological structures of each industry are shown in Supplementary Figure 13 to Supplementary Figure 18 and Supplementary Table 7 to Supplementary Table 12.

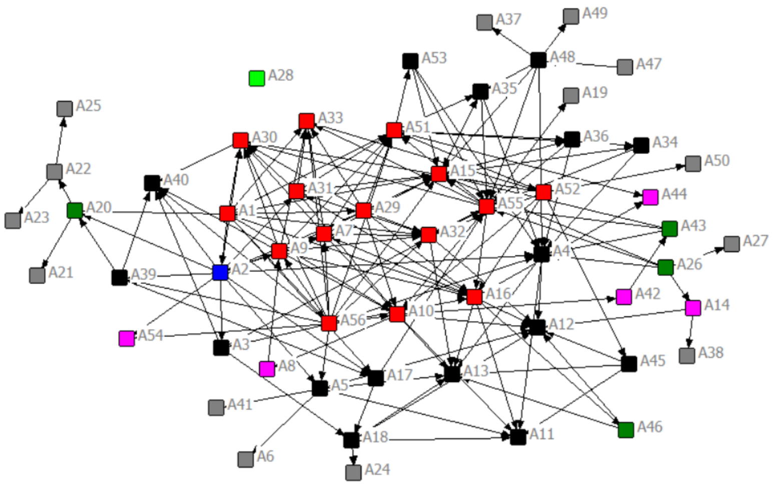

Supplementary Figure 13 Saline lake industry (SLI) topological structure. Number A represents the saline lake Industry, and different serial numbers represent different technologies, with information specific to each technology provided in Supplementary Data 1.

Supplementary Table 7 The degree centrality (DC) and betweenness centrality (BC) of each node in the saline lake industry (SLI) network.

No.	Nomenclature	DC(%)	BC(%)	Core nodes identification
A1	N - Saltern to carnallite	12.73	0.16	1
A2	N - KCl by reverse-current trip flotation	12.73	0.64	1
A3	N - K_2SO_4 by double decomposition	4.55	0.19	1
A4	N - K_2CO_3 by electrolysis process	12.73	1.12	1
A5	N - KOH	8.18	0.34	1
A6	N - $KMnO_4$	0.91	0	0

A7	N - K by substitution method	9.09	0.94	1
A8	N - $NaNO_3$ by neutralization	3.64	0.72	1
A9	N - KNO_3	10	1.13	1
A10	N - Chlor-alkali industry	15.46	4.13	1
A11	N - Production of ADC foaming agent	6.36	0	0
A12	N - PVC by calcium carbide	10.91	0	1
A13	N - HCl	10	0	1
A14	N - Production of calcium carbide	2.73	0.05	1
A15	N - Na_2CO_3 by alkaline process	16.36	1.71	1
A16	N - Na by electrolysis	12.73	2.39	1
A17	N - Dehydration of old brine	4.55	1.22	1
A18	N - Mg by electrolysis	5.46	0.54	1
A19	N – Downstream of $MgCO_3$	0.91	0	0
A20	N - $Mg(OH)_2$ by NH_3 precipitation	4.55	0.67	1
A21	N - Downstream of $Mg(OH)_2$	0.91	0	0
A22	N - MgO	2.73	0.4	1
A23	N - Fireclay brick by MgO	0.91	0	0
A24	N - Magnesium alloy and pressure contact-die-casting	0.91	0	0
A25	N - $MgSO_4$	0.91	0	0
A26	N - Limestone calcination	4.55	0	0
A27	N - CaO	0.91	0	0
A28	N - Reinforced glass	0	0	0
A29	N - Kainite	10.91	0	1
A30	N - Potash magnesium sulphate fertilizer	12.73	0.65	1
A31	N - $KClO_4$	8.18	1.04	1
A32	N - $NaClO_3$	10	0.97	1
A33	N - NaCl	7.27	0	1
A34	N - $C_4H_7NaO_4$ from Na_2CO_3	4.55	0.43	1
A35	N - $Na_2S_2O_3$ from Na_2S	5.46	0.43	1
A36	N - $Na_2S_2O_3$ from Na_2SO_3	4.56	0.43	1
A37	N - $Na_2S_2O_3$ from Na_2S	0.91	0	0
A38	N- C_2H_2 from calcium carbide	0.91	0.2	1
A39	N - K_2SO_4 from saline lake	4.55	0	0
A40	N - Compound fertilizer	5.46	0	1
A41	N - Potassium Oleate	0.91	0	0
A42	N - HCOONa from CO	1.82	0.88	0
A43	N - $Na_2S_2O_4$ from HCOONa	3.64	0.94	1
A44	N - Na_2O_2	1.82	0	0
A45	N - Ca by electrolysis	3.64	0.04	1
A46	N - $NaNH_2$	2.73	0	0
A47	N - Na_2SO_4 from mirabilite	0.91	0	0
A48	N - Na_2S	6.36	1.04	1
A49	N - Sodium polysulfide	0.91	0	0

A50	N – Downstream of $CaCl_2$	0.91	0	0
A51	N - Na_2CO_3 by ammonia soda process	12.73	4.81	1
A52	N - $CaCl_2$ from waste ammonia liquid	9.09	2.24	1
A53	N - $NaNO_3$ by neutralization	3.64	0.1	1
A54	N - KNO_3 by neutralization	1.82	0	0
A55	N - $MgCO_3$ by $MgCl_2$	16.36	10.08	1
A56	N - Recovery of K and Na from K tail salt	15.46	0.16	1

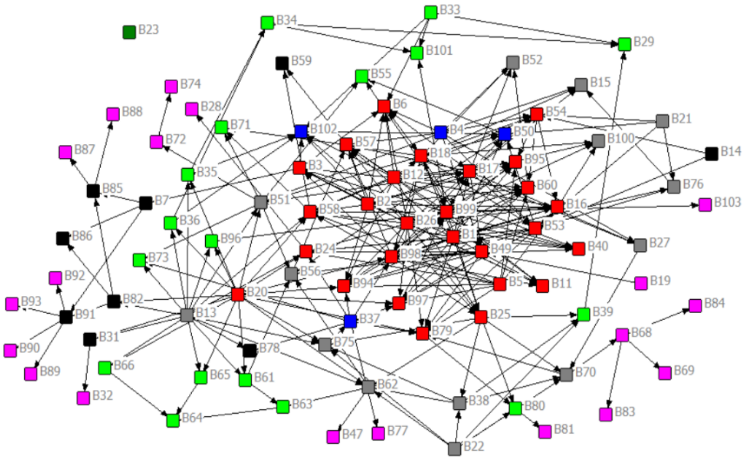

Supplementary Figure 14 Organic chemical industry (OCI) topological structure. Number B represents the organic chemical industry, and different serial numbers represent different technologies, with information specific to each technology provided in Supplementary Data 1.

Supplementary Table 8 The degree centrality (DC) and betweenness centrality (BC) of each node in the organic chemical industry (OCI) network.

No.	Nomenclature	DC(%)	BC(%)	Core nodes identification
B1	N - Coking	10.56	0	1
B2	N - CH_3OH from coal	7.78	0	1
B3	N - MTO	5	4.58	1
B4	N - NH_3 from coal	6.67	0	1
B5	N - Syngas from coal	5	0	1
B6	N - Urea from NH_3	6.11	1.06	1
B7	N - PVC by oxy-chlorination	2.22	3.82	0
B11	N - DME from CH_3OH	3.33	0	0
B12	N - Dimethyl ether from coal	7.78	0	1

B13	N - Thermal cracking of natural gas (1)	8.89	0	1
B14	N - Thermal cracking of natural gas (2)	1.11	0	0
B15	N - Hexamethylenetetramine	2.22	0	0
B16	N - Dimethyl carbonate by methanol carbonyl process	5.56	0.55	1
B17	N - DMC by direct alcoholysis of urea	10.56	3.55	1
B18	N - Dimethyl carbonate by indirect urea method	10.56	3.55	1
B19	N - Oil refining (1)	0.56	0	0
B20	N - Oil refining (2)	10.56	0	1
B21	N - Oil refining (3)	2.22	0	0
B22	N - Oil refining (4)	3.33	0	0
B23	N - Oil refining (5)	0	0	0
B24	N - Polypropylene	6.67	0.45	1
B25	N - CH_3OH to aromatics	6.67	6.42	1
B26	N - CH_3OH from natural gas	10.56	0	1
B27	N - HNO_3 by ammoxidation	2.22	0.35	1
B28	N - CPP	0.56	0	0
B29	N - PET synthesis	1.67	0	0
B31	N - Polyethylene	1.67	0.03	1
B32	N - CPE	0.56	0	0
B33	N - Glycol from coal	2.22	0	0
B34	N - Glycol from ethylene oxide	2.22	1.69	1
B35	N - Ethylene oxide by oxidation	2.22	0.04	1
B36	N - Ethylene oxide by chlorohydrin method	2.22	0.04	1
B37	N - Styrene by ethylbenzene dehydrogenation	5	3.34	1
B38	N - Paraxylene by toluene disproportionation method	3.33	0	0
B39	N - P-phthalic acid	2.22	0.38	1
B40	N - Methylmercaptan	3.33	0	0
B47	N - Triphenyl phosphite	0.56	0	0
B49	N - HCN by light oil cracking method	6.11	1.72	1
B50	N - HCN from CH_4 and NH_3	4.44	0.32	1
B51	N - Acrylonitrile by propylene ammoxidation	5.56	1.57	1
B52	N - Acetonitrile from NH_3 and CH_3COOH	2.22	0	0
B53	N - Acetonitrile from NH_3 and C_3H_8	6.67	2.41	1
B80	N - Isocyanate	2.22	0.48	1
B81	N - Polyurethane	0.56	0	0
B82	N - PVC from C_3H_6	2.78	0.23	1
B83	N - Rubber absorber antioxidant	0.56	0	0
B84	N - Downstream of nitrochlorobenzene	0.56	0	0
B85	N - CPVC	2.22	0.95	1
B86	N - UPVC	1.11	0	0
B87	N - Pipe CPVC	0.56	0	0
B88	N - CPVC pipe	0.56	0	0

B89	N - Plate CPVC	0.56	0	0
B90	N - Pipe CPVC	0.56	0	0
B91	N - Speciality resin	3.33	1.9	1
B92	N - PVC wire	0.56	0	0
B93	N - PVC plastic doors and windows	0.56	0	0
B94	N - Chloromethane	7.22	0	1
B95	N - Methyl acetate from CH_3OH	5	0.5	1
B96	N - Ethyl alcohol from C_2H_4	1.67	0.4	1
B97	N - CH_3CH_2OH from CH_3COOH	5	1.05	1
B98	N - Ethyl alcohol from CH_3COOH	11.67	8.12	1
B99	N - Diethyl carbonate	12.78	10.32	1
B100	N - Ethyl methyl carbonate by ester exchange method	2.22	0	0
B101	N - Ethylene carbonate	2.22	2.87	1
B102	N - Ethylene carbonate ethylene oxide	4.44	0	1
B103	N - Propylene carbonate	1.11	0	0

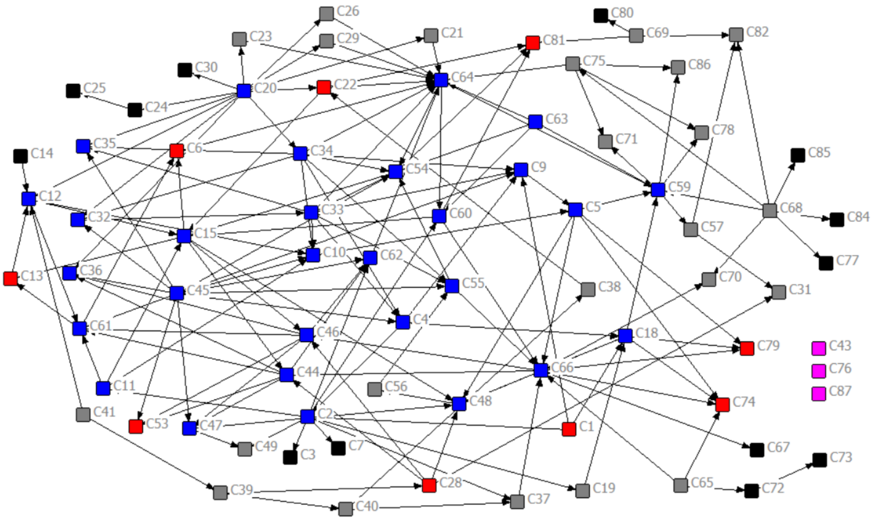

Supplementary Figure 15 Inorganic chemical industry (IOCI) topological structure. Number C represents the inorganic chemical industry, and different serial numbers represent different technologies, with information specific to each technology provided in Supplementary Data 1.

Supplementary Table 9 The degree centrality (DC) and betweenness centrality (BC) of each node in the inorganic chemical industry (IOCI) network.

No.	Nomenclature	DC(%)	BC(%)	Core nodes identification
C1	N - HF from fluorspar	1.97	0	0

C2	N - Hydrofluoric acid from HF	6.58	1.54	1
C3	N - Electronic grade HF	0.66	0	0
C4	N - NF_3 by synthesis	4.61	1.33	0
C5	N - NF_3 by electrolysis	4.61	2.35	1
C6	N - NaF from Na_2SiF_6	1.97	1.39	1
C7	N - KF by neutralization	0.66	0	0
C9	N - NH_4F from NH_3 and HF	3.95	0.21	1
C10	N - NH_4HF_2 from H_2SiF_6	3.95	1.81	1
C11	N - Acidolysis of silicate	2.63	0.35	1
C12	N - H_2SiF_6 by SiF_4 hydrolysis	4.61	2.98	1
C13	N - SiF_4 from Na_2SiF_6	1.97	2.07	1
C14	N - SiF_4 from fluorspar	0.66	0	0
C15	N - Na_2SiF_6	6.58	6.12	1
C18	N - F_2 by electrolysis	5.92	1.63	1
C19	N - KHF_2 from KOH	1.32	0.14	1
C20	N - H_3PO_4 by wet process	7.9	0	1
C21	N - $Na_5P_3O_{10}$	1.32	0.03	1
C22	N - $CaHPO_4$ from $CaCl_2$	3.29	1.11	1
C23	N - $CaHPO_4$ from $CaCO_3$	1.32	0.03	1
C24	N - NaH_2PO_4	1.32	0.02	1
C25	N - $(NaPO_3)_6$	0.66	0	0
C26	N - Na_3PO_4	1.32	0.03	1
C28	N - H_3PO_3	3.29	0.51	1
C29	N - KH_2PO_4 from K_2CO_3	1.32	0.03	1
C30	N - KH_2PO_4 from KOH	0.66	0	0
C31	N - $Na_2HPO_3 \cdot 5H_2O$	1.32	0	0
C32	N - $(NH_4)_3PO_4$	3.29	0.05	1
C33	N - Ammonium polyphosphate from $(NH_4)_3PO_4$	5.92	0.75	1
C34	N - Ammonium polyphosphate from H_3PO_4	5.92	0.34	1
C35	N - Ammonium polyphosphate from H_3PO_4	2.63	0	0
C36	N - BPO_4	2.63	0	0
C37	N - PF_5 from HF	1.97	0.47	1
C38	N - PF_5 from CaF_2	1.32	0.46	1
C39	N - PCl_3	1.97	0.24	1
C40	N - PCl_5	1.97	0.32	1
C41	N - P	1.32	0	0
C43	N - S	0	0	0
C44	N - H_3BO_3 from ludwigite and HCl	5.26	3.28	1
C45	N - H_3BO_3 from ludwigite	7.9	0	1
C46	N - H_3BO_3 from saline brine	5.26	3.28	1
C47	N - B_2O_3	3.29	0.79	1

C48	N - B	4.61	2.39	1
C49	N - BF_3 from B_2O_3	1.32	0	0
C53	N - B_4C	1.97	0	0
C54	N - BN from borax and NH4Cl	5.26	2.72	1
C55	N - Boronnitride	3.95	2.48	1
C56	N - BCl_3	1.32	0	0
C57	N - KBH_4	1.97	1.61	1
C59	N - $NaBH_4$	5.92	4.8	1
C60	N - KBF_4 from HF	3.95	3.68	1
C61	N - KBF_4	4.61	1.42	1
C62	N - $LiBF_4$	3.29	2.45	1
C63	N - Borax	2.63	0	0
C64	N - Borax by alkali carbonate method	9.21	8.84	1
C65	N - Si for industry	1.97	0	0
C66	N - Polysilicon by siemens method	9.21	7.49	1
C67	N - Monocrystalline Si	0.66	0	0
C68	N - Quartz sand	4.61	0	1
C69	N - Silicon materials	1.97	0	0
C70	N - Optical fiber	1.32	0	0
C71	N - Silica gel	1.32	0	0
C72	N - Organosilicon	1.32	0.02	1
C73	N - Downstream of organosilicon	0.66	0	0
C74	N - Silane	2.63	0	0
C75	N - $Na_2O{\cdot}nSiO_2$	3.29	0.47	1
C76	N - Glass	0	0	0
C77	N - SiO_2	0.66	0	0
C78	N - Silica white by precipitation	1.32	0	0
C79	N - Silica white by gas phase method	2.63	0	0
C80	N - Black silicon carbide	0.66	0	0
C81	N - Green silicon carbide	2.63	0	0
C82	N - Crystal by hydrothermal synthesis	1.97	0	0
C84	N - Quartz product	0.66	0	0
C85	N - Downstream of quartz	0.66	0	0
C86	N - Glass microspheres	1.32	0	0
C87	N - Silicocalcium	0	0	0

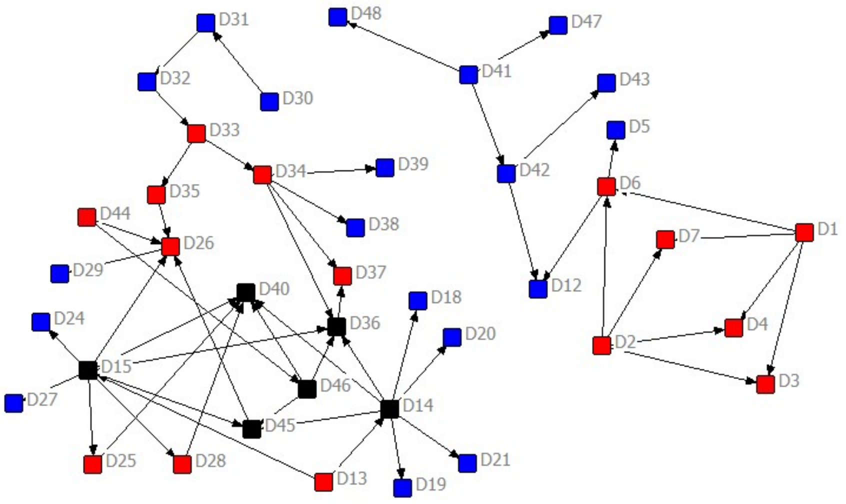

Supplementary Figure 16 Metal smelting and processing industry (MSPI) topological structure. Number D represents the metal smelting and processing industry, and different serial numbers represent different technologies, with information specific to each technology provided in Supplementary Data 1.

Supplementary Table 10 The degree centrality (DC) and betweenness centrality (BC) of each node in the metal smelting and processing industry (MSPI) network.

No.	Nomenclature	DC(%)	BC(%)	Core nodes identification
D1	N - $SrCO_3$ by carbon reduction	5.13	0	1
D2	N - $SrCO_3$ by double decomposition	5.13	0	1
D3	N - $Sr(NO_3)_2$	2.56	0	0
D4	N - $Sr(OH)_2$	2.56	0	0
D5	N - SrO_2	1.28	0	0
D6	N - SrO	5.13	0.27	1
D7	N - $SrCl_2$	2.56	0	0
D12	N - Sr	2.56	0	0
D13	N - Lead-zinc ore dressing	2.56	0	0
D14	N - Pb	10.26	0.41	1
D15	N - Zinc leaching	11.54	0.54	1
D18	N - Pb profile	1.28	0	0
D19	N - PbO	1.28	0	0
D20	N - Lead alloy	1.28	0	0
D21	N - Pb compound	1.28	0	0
D24	N - Zinc alloy	1.28	0	0
D25	N - Hot-galvanizing	2.56	0	0

D26	N - Brass	6.41	0.74	1
D27	N - ZnO	1.28	0	0
D28	N - Zn profile	2.56	0	0
D29	N - Brass profile	1.28	0	0
D30	N - Beneficiation of nickel cobalt ore	1.28	0	0
D31	N - Ni	2.56	0.68	1
D32	N - Low grade Ni matte by blowing	2.56	1.22	0
D33	N - High grade Ni matte by grinding-flotation	3.85	1.62	1
D34	N - Electrolysis of nickel concentrate	6.41	1.08	1
D35	N - Copper concentrate electrolysis	2.56	0.54	1
D36	N - $NiSO_4$	6.41	0.34	1
D37	N - $Ni(OH)_2$	2.56	0	0
D38	N - $Ni(CO)_4$	1.28	0	0
D39	N - Co_3O_4	1.28	0	0
D40	N - ZnO from waste bearing zinc	6.41	0	1
D41	N - Al_2O_3	3.85	0	1
D42	N - Al by electrolysis	3.85	0.14	1
D43	N - Al profile	1.28	0	0
D44	N - Copper pyrometallurgy	2.56	0	0
D45	N - Hydrometallurgical Cu refining	5.13	0.27	1
D46	N - H_2SO_4	5.13	0.27	1
D47	N - $AlCl_3$	1.28	0	0
D48	N - AlF_3	1.28	0	0

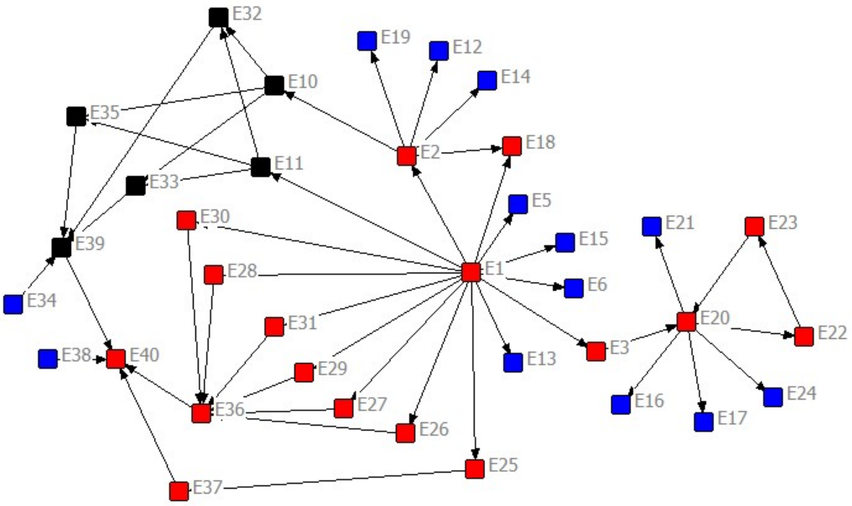

Supplementary Figure 17 Lithium deep processing industry (LDPI) topological structure. Number E represents the lithium deep processing industry, and different serial numbers represent different technologies, with information specific to each technology provided in Supplementary Data 1.

Supplementary Table 11 The degree centrality (DC) and betweenness centrality (BC) of each node in the lithium deep processing industry (LDPI) network.

No.	Nomenclature	DC(%)	BC(%)	Core nodes identification
E1	N - Li_2CO_3 from saline brine	21.43	0	1
E2	N - LiOH	8.57	0.34	1
E3	N - LiCl	2.86	0.59	1
E5	N - CH_3COOLi	1.43	0	0
E6	N - Li_2SO_4	1.43	0	0
E10	N - LiF from LiOH	5.71	0.42	1
E11	N - LiF from Li_2CO_3	5.71	0.34	1
E12	N - $LiNO_3$ from LiOH	1.43	0	0
E13	N - $LiNO_3$ from Li_2CO_3	1.43	0	0
E14	N - Li_3PO_4 from LiOH	1.43	0	0
E15	N - Li_3PO_4 from Li_2CO_3	1.43	0	0
E16	N - Li and Al alloy	1.43	0	0
E17	N - Li and Mg alloy	1.43	0	0
E18	N - LiH_2PO_4	2.86	0	0
E19	N - LiO_3P	1.43	0	0
E20	N - Li	10	1.77	1
E21	N - Lithium foil	1.43	0	0
E22	N - LiH	2.86	0.25	1
E23	N - $LiAlH_4$	2.86	0.42	1
E24	N - Downstream of Li+	1.43	0	0
E25	N - $Li_4Ti_5O_{12}$	2.86	0.1	1
E26	N - $LiFePO_4$	2.86	0.03	1
E27	N - NMC	2.86	0.03	1
E28	N - $LiNiO_2$	2.86	0.03	1
E29	N - $LiMn_2O_4$	2.86	0.03	1
E30	N - Lithium nickel cobalt aluminum oxide	2.86	0.03	1
E31	N - $LiCoO_2$	2.86	0.03	1
E32	N - $LiPF_6$ from ion exchange method	4.29	0.2	1
E33	N - $LiPF_6$ from PF_5	4.29	0.2	1
E34	N - C_4BLiO_8	1.43	0	0

E35	N - $LiBF_4$ from BF_3	4.29	0.2	1
E36	N - Cathode material of lithium ion batteries	10	0.58	1
E37	N - Anode materials for lithium-ion batteries	2.86	0.1	1
E38	N - Li-ion battery separator material	1.43	0	0
E39	N - Lithium-ion battery electrolyte	7.14	0.59	1
E40	N - Lithium ion battery	5.71	0	0

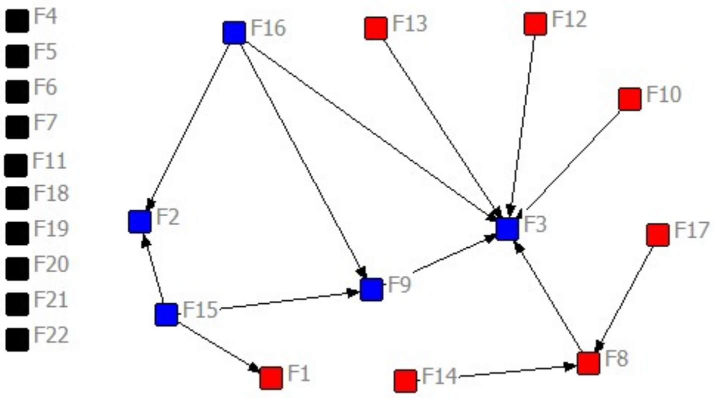

Supplementary Figure 18 Comprehensive waste utilization industry (CWUI) topological structure. Number F represents the comprehensive waste utilization industry, and different serial numbers represent different technologies, with information specific to each technology provided in Supplementary Data 1.

Supplementary Table 12 The degree centrality (DC) and betweenness centrality (BC) of each node in the comprehensive waste utilization industry (CWUI) network.

No.	Nomenclature	DC(%)	BC(%)	Core nodes identification
F1	N - Dinas bricks	2.38	0	0
F2	N - Aerated block	4.76	0	1
F3	N - Concrete	14.29	0	1
F4	N - Geomembrane	0	0	0
F5	N - PE tube	0	0	0
F6	N - Magnesium oxychloride cement	0	0	0
F7	N - Pyrites from coalgangue	0	0	0

F8	N - Cement from coalgangue	7.14	0.48	1
F9	N - Baking-free bricks from coalgangue	7.14	0.24	1
F10	N - Molecular sieve from coalgangue	2.38	0	0
F11	N - Manure from coalgangue	0	0	0
F12	N - $AlCl_3$ from coalgangue	2.38	0	0
F13	N - $Al_2(SO_4)_3$ from coalgangue	2.38	0	0
F14	N - Epoxyethane	2.38	0	0
F15	N - $CaCO_3$ from carbide slag	7.14	0	1
F16	N - Cement from carbide slag	7.14	0	1
F17	N - K_2SO_4 by IMI method	2.38	0	0
F18	N - Molten salt	0	0	0
F19	N - Extraction of Rb and Cs	0	0	0
F20	N - Refine of Rb	0	0	0
F21	N - Refine of Cs	0	0	0
F22	N - Fly ash disposal	0	0	0

Supplementary Method 5 Scenario settings

To meet the exigencies of climate policy and accomplish decarbonization within the ambit of development, several approaches are designed. These include: i) gradually decarbonizing from the initial high-energy-consuming industrial structure through model guidance, by regulating node size, eradicating inefficient nodes, and cultivating high-efficiency nodes through heuristic strategies; ii) gradually decarbonizing by introducing certain measures of carbon reduction, such as carbon capture, utilization, and storage (CCUS), clean electricity, and steam boiler retrofit. We analyze the reduction effects of these two approaches both separately and in conjunction while contemplating the evolutionary trend of the industrial structure by allotting the degree of implementation of these measures at different junctures, to foster a dynamic evolution of the industrial structure that pursues sustainable developmental objectives. Here, our sustainable developmental objective is defined as maximizing economic objectives while minimizing energy consumption, resource consumption, and environmental impact as much as feasible. For instance,

in the process of developing the same amount of brine from the saline lake, we can augment the economic added value of raw materials by implementing measures such as extending the industrial chain, regulating the industrial structure, introducing high-end nodes, and eliminating inefficient processes. This will enable scarce resources to realize long-term development with heightened economic value, the scenario settings are shown in Supplementary Figure 19.

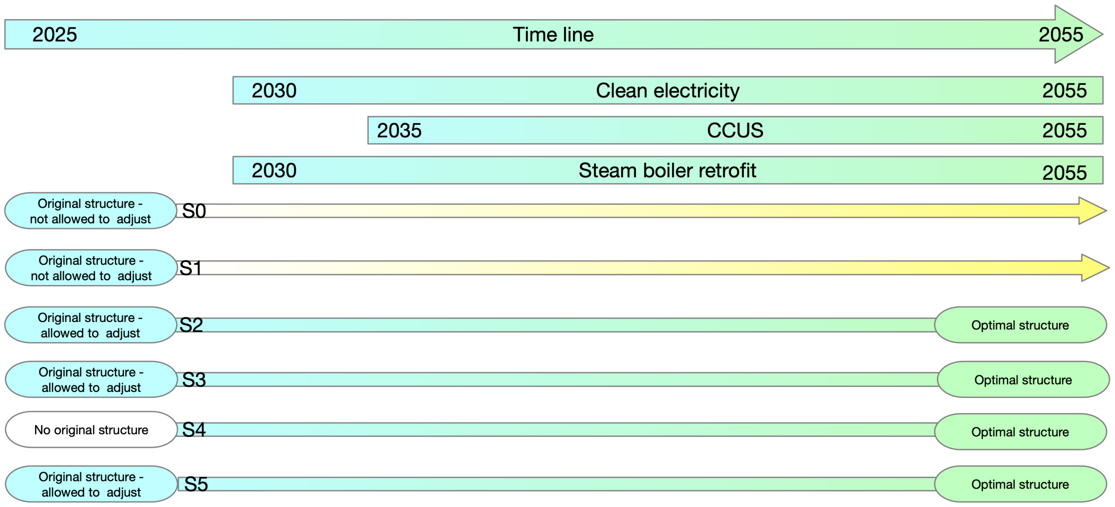

Scenarios	T1	T2	T3	T4	T5
S0	Conservative	No	/	Yes	Yes
S1	Conservative	No	/	No	Yes
S2	Radical	Yes	10 years	Yes	Yes
S3	Radical	Yes	5 years	Yes	Yes
S4	Radical	Yes	/	Yes	Yes
S5	Radical	Yes	20 years	Yes	Yes

Supplementary Figure 19 Scenario settings of S0 to S5. Detailed scenarios include S0 to S5, such as S0, developed under the original structure and gradually introduced the technologies recommended by the resource-based regional industrial economy development optimization model (RRIEDOM); S1, developed with certain constraints on the industrial structure to simulate the empirical pattern; S2, developed under the original structure, allowing a 10-year transition to the optimal structure by identifying and eliminating the low-efficiency technologies and gradually deploying the technologies recommended by RRIEDOM; S3, similar to S2, but with a transition period of 5 years; S4, assumes that there is no original

structure and develops only under the instruction of RRIEDOM with the interaction of available technologies database; S5, similar to S2, but with a longer transition period of 20 years. There are 5 settings about T1 to T5, such as T1: radical/conservative attitude towards the transformation process; T2: whether to consider changing the original industrial structure; T3: number of years of transition from the original industrial structure; T4: whether to develop according to the guidance of RRIEDOM model; T5: whether to gradually impose carbon-negative technologies.

We initially derived the optimal industrial structure based on the local resource endowment within the industrial zone using the RRIEDOM model. However, we recognized the importance of understanding how to transition from the existing local structure to the optimal one, as such a transition carries significant relevance and can offer valuable guidance to local policymakers. We were particularly concerned about the potential impact of the transition duration. For instance, an excessively long transition period, such as 20 years, might signify a more radical approach, possibly leading to issues related to alignment with the current economic landscape and financial challenges. Conversely, a very short transition period, such as 5 years, might result in excessive emissions and technology iteration challenges. To address this concern, we devised three distinct scenarios spanning 5 years (S3), 10 years (S2), and 20 years (S5) to assess and compare the consequences of varying transition durations.

In the context of traditional industrial planning, characterized by expert-driven decision-making, there may be a predisposition to bolster existing strong industries. For example, if a particular region is predominantly associated with the coal chemical industry, subsequent

development efforts may lean toward downstream sectors linked to coal chemicals. While this inertia leverages existing industrial infrastructure and well-established expertise, it can also foster industry dependency, stymying the dynamism required for structural transformation. Consequently, in response to this concern, we established scenario S1, which imposes limitations on the growth of each industry and restricts fluctuations in the contribution of each industry to the overall network by no more than 5% relative to the base structure. This scenario allows us to explore the outcomes under the influence of traditional inertia-driven decision-making.

To investigate the model's guiding implications, we introduced scenario S0, wherein future development steps on the existing local industrial structure within the industrial zone are meticulously guided by the model. This approach represents a more conservative development strategy, as it involves making incremental changes to the existing local industries.

Lastly, to explore the maximum developmental potential of the local area, we introduced scenario S4. In this scenario, there are no pre-existing local industrial structures, substantial capital and technological reserves are available for harnessing local resource endowments, and the government exhibits ambitious economic and environmental expectations to stimulate development. Within this context, scenario S4 represents the epitome of local developmental potential for the industrial zone, at least in terms of optimizing the utilization of available chemical industry technologies. The following are the detailed descriptions of each scenario setting.

In scenario S0, we posit that a fully-fledged commercial industrial framework is already functioning efficiently in the local industrial district, encapsulating the current authentic local industrial configuration. Utilizing the RRIEDOM model as a developmental compass, we aspire that each prospective local developmental objective adheres to the RRIEDOM model. Despite the

presence of certain local inefficiencies, these are neither eliminated nor diminished of technologies throughout the development process, instead, they persist and evolve within the pre-existing infrastructure. This depicts an optimal development trajectory under the auspices of the model while maintaining the original structure intact. Under this scenario, our objective is to scrutinize the full developmental landscape when the local government adopts the most conservative posture, hypothesizing that the local government is unable to accommodate any potential detriment or impairment to the capital invested in ongoing projects. The model is designed in a way where each prevailing local industrial technology scale is subject to a lower-bound constraint. This necessitates that the model, during the development process, should operate at least at the minimum scale of the respective industrial technology, prohibiting any scale reductions.

In scenario S1, we hypothesize that the industrial district evolves from the original starting structure, maintaining the initial industrial configuration unaltered, and does not discard the initial inefficient technology during the development process. Within this scenario, we employ a constraint to curtail the evolution of the industrial structure, i.e., the development still fluctuates up and down around the original industrial ratio by no more than 5%. Through this constraint, we aim to investigate the limitations on the industrial district's evolution predicated on the initial industrial structure, thus simulating the feasibility and drawbacks of the empirical development. As based on the empirical development, the industrial growth of the industrial district is likely to lean towards a structure more or less akin to the original. Because the industrial expansion of the district is likely to be built upon the existing industrial structure to amplify the scale or judiciously extend the downstream industry chain, and consequently, the proportion of the industrial structure may not experience significant fluctuation, we adopt a 5% constraint as a means of exploration. However,

this constraint can also be progressively relaxed.

In scenario S2, we postulate that the local evolution of the industrial district is still predicated on the extant industrial projects. We use the RRIEDOM model as a developmental guide, such that every future goal of local evolution will adhere to the RRIEDOM model. However, differing from S0, we permit a certain elimination gradient as a criterion during the development process, which gradually diminishes the scale or eliminates the inefficient technologies within the original industrial structure, also as adjudicated by RRIEDOM. In this scenario, we envisage a time constraint of 10 years to explore the potential changes in the path of transitioning from the original industrial structure to the optimal industrial structure. In this scenario, we seek to comprehend the opportunities and challenges that may stem from a change in the path from the original industrial structure to the optimal one. Here, we anticipate a more realistic developmental model or path, and a feasible trajectory for regional industrial evolution, since we allow for inefficient technologies to be reduced or eradicated to satisfy the model's target requirements. The same holds for actual industrial evolution, which will enact additional adjustments to the existing industrial structure due to increasingly stringent environmental policies, technological progress, and efficiency demands. However, unlike these empirical adjustments, we make amendments based on a machine simulation perspective, offering a more objective and generalizable approach.

In scenario S3, which parallels S2 in many ways, we venture to examine the differential outcomes if the rate of transformation were accelerated. In this scenario, we aspire to simulate the conduct of a more transformative government, wherein policymakers aim to swiftly alter paths to fulfill their responsive environmental, economic, and other objectives as swiftly as possible.

In scenario S4, we opt to adopt a decidedly optimistic perspective regarding the development

and utilization path of regional resources. We posit that local resource development within the industrial zone is in its nascent stages, with the local government possessing both the ample funding and ambition necessary for resource development. We assume the existence of robust and transparent technical information, with decisions grounded in objective facts, following established planning principles and minimizing potential human factors. This scenario seeks to simulate the dynamics of an optimal regional resource development pathway, where the development plans for each type of resource, the deployment pattern of each technology, and the extension of each industrial chain are guided by the RRIEDOM model, all while achieving predetermined economic objectives.

In scenario S5, we maintain similarities to S2 and S3, yet deviate in our exploration of the probable behavior of a more conservative policymaker. This manifests in a slower, more conservative pace of industrial restructuring, like 20 20-year transition, where the government elects to take a cautious approach, allowing for additional time to advance industry securely. The aim is to realize a more robust development model, while concurrently minimizing the negative impacts associated with the phasing out of inefficient technologies, such as potential capital losses.

To illustrate the transition from the original industrial structure to the optimal structure guided by RRIEDOM, we created Supplementary Figure 20 by randomly selecting two data panels from the time interval. Additionally, we have generated figures for various aspects, including all types of products and production achievable within the industrial network under the optimal structure, as well as the size and capacity of each node (Supplementary Figure 22), electricity consumption (Supplementary Figure 23), solid waste generation (Supplementary Figure 24), water consumption (Supplementary Figure 25), capital investment (Supplementary Figure 26), energy consumption

(Supplementary Figure 27), profit (Supplementary Figure 28), production value (Supplementary Figure 29), and carbon emissions Supplementary Figure 30). Detailed results for the remaining data are provided in the Supplementary Data 1.

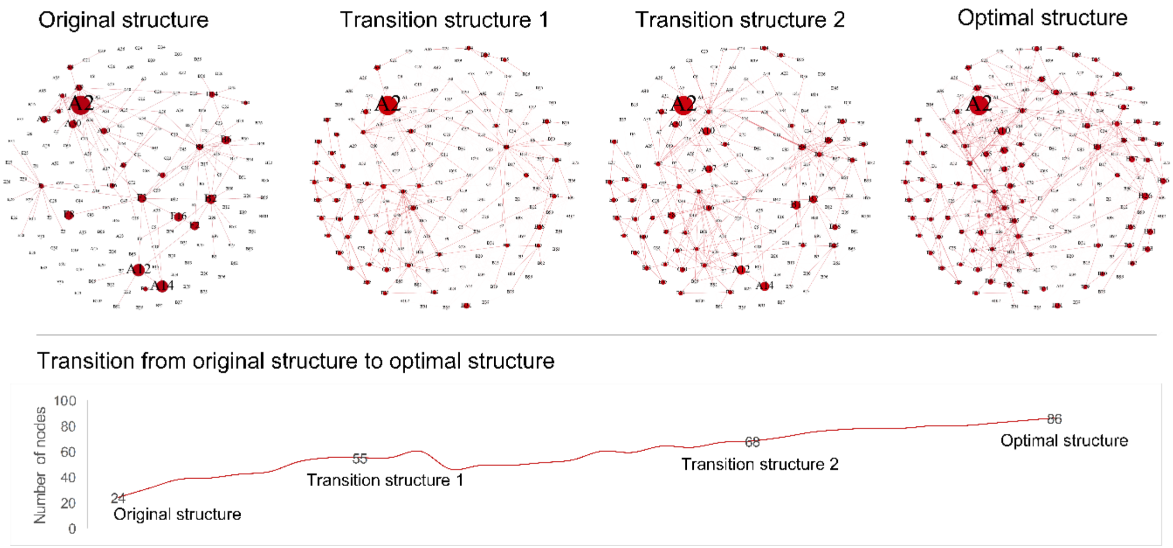

Supplementary Figure 20 Transition from original structure to optimal structure. The original structure has 24 technologies and the optimal structure has 86 technologies, and the model guides the phasing out of existing technologies and investment in new ones until the network is optimized. transition structure 1 and transition structure 2 are randomly selected as examples to illustrate the process of transition.

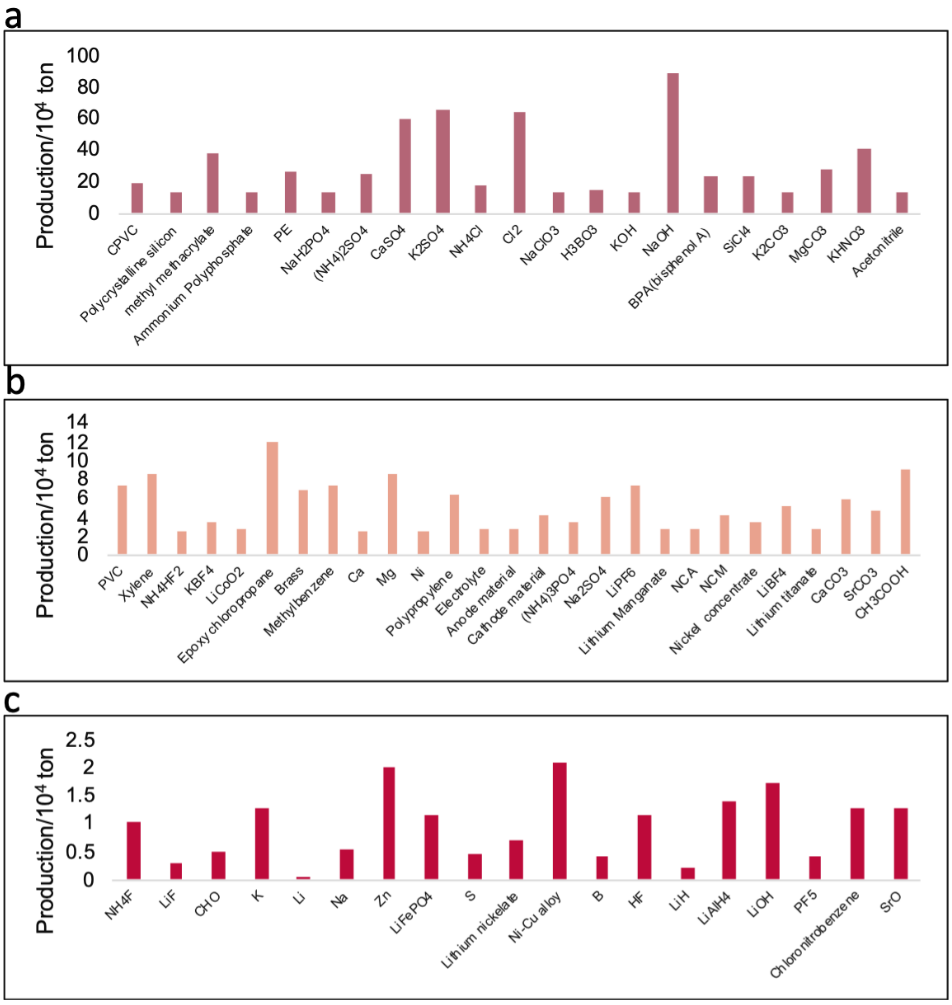

Supplementary Figure 21 Main products and the capacity of the optimal industrial structure. The industrial network under the optimal structure would produce major products, Including large-scale products (a), e.g. caustic soda, sodium sulfate, etc.; medium-scale products (b), e.g. polyvinyl chloride (PVC), magnesium metal, calcium metal, etc.; small-scale products (c), e.g. lithium metal, lithium hydroxide, etc.

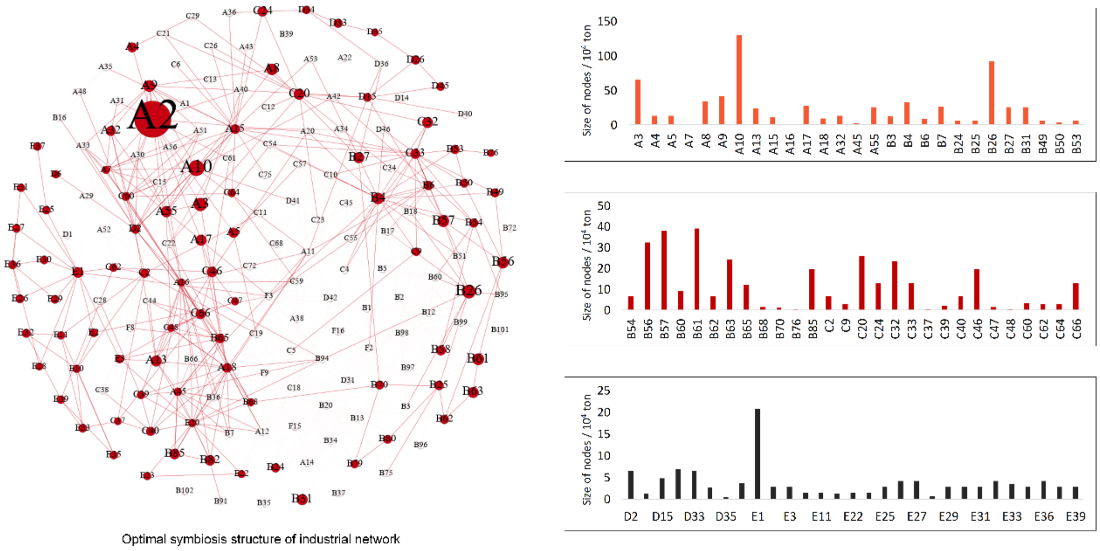

Supplementary Figure 22 Capacity of each node of the optimal industrial structure. Most of the lithium-related technologies under the optimal structure have capacity, with the largest technology across the network being A2 - Potassium Extraction from Salt Lakes. Detailed information about each technology can be found in Supplementary Data 1.

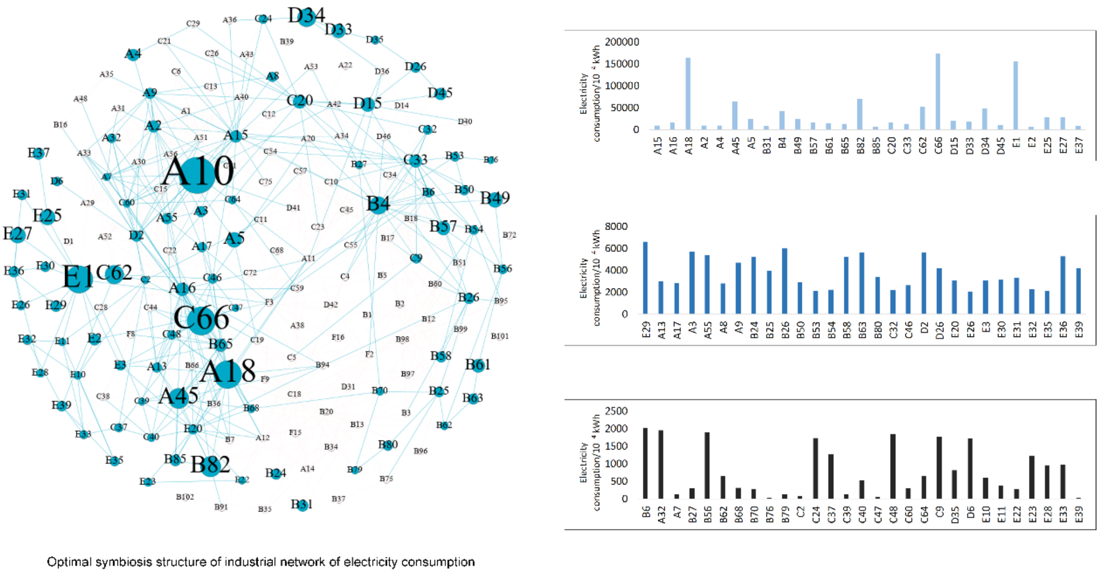

Supplementary Figure 23 Electricity consumption of each node of the optimal industrial structure. The technologies that consume the most power across the network in the optimal

structure are A10, C66, A18, E1, etc. Detailed information about each technology can be found in Supplementary Data 1.

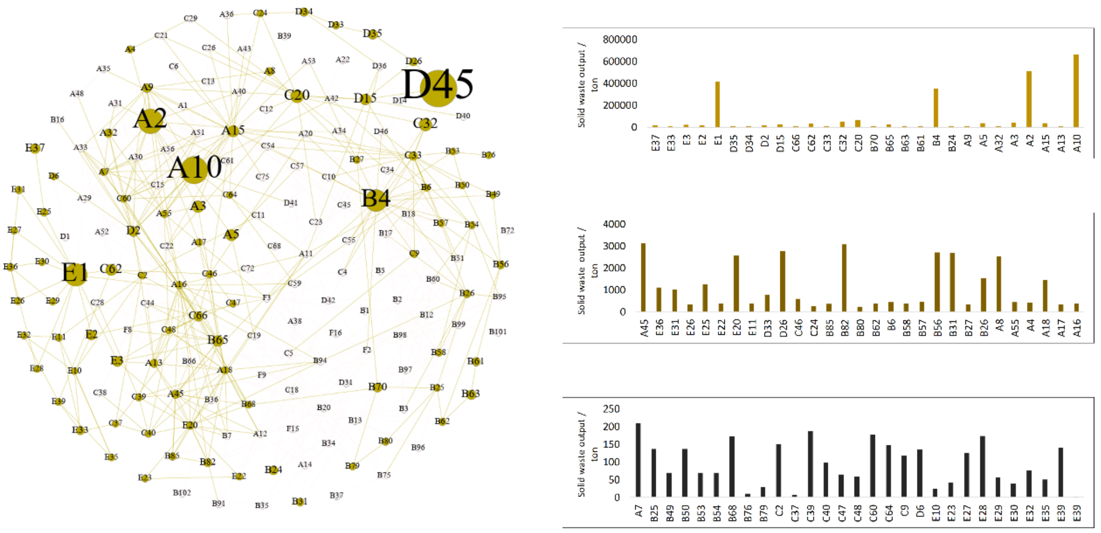

Optimal symbiosis structure of industrial network of solid waste output

Supplementary Figure 24 Solid waste output of each node of the optimal industrial structure. The technologies with the highest solid waste generation across the network under the optimal structure are A2, A10, B4, D45, and E1. Detailed information about each technology can be found in Supplementary Data 1.

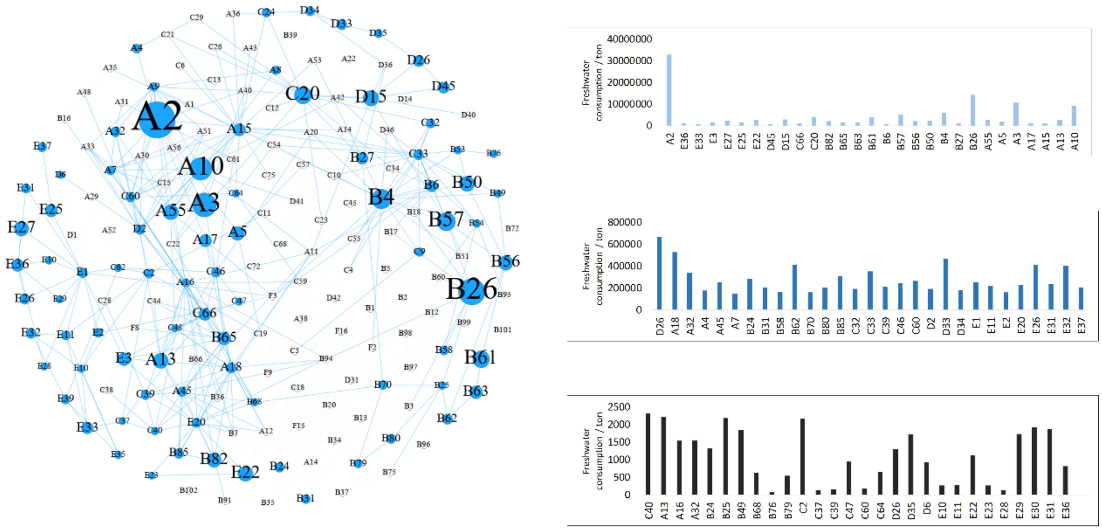

Optimal symbiosis structure of industrial network of freshwater consumption

Supplementary Figure 25 Freshwater consumption of each node of the optimal industrial structure. The technologies with the highest fresh water consumption across the network under the optimal structure are A10, C66, A18, B26, B56, B57, C20, and D15. Detailed information on each technology can be found in Supplementary Data 1.

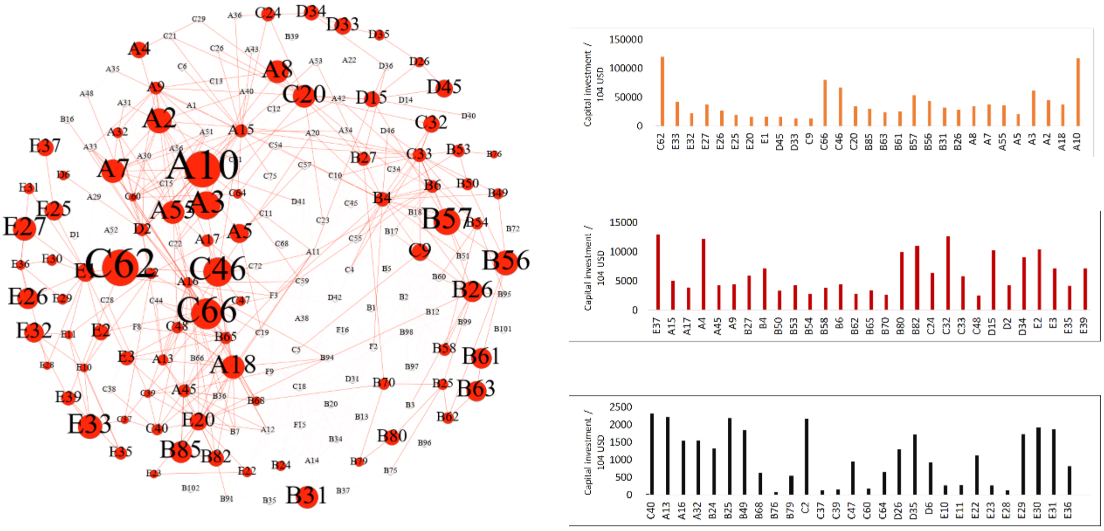

Optimal symbiosis structure of industrial network of capital investment

Supplementary Figure 26 Capital investment of each node of the optimal industrial structure. The techniques with the largest investment in the entire network under the optimal architecture are

A10, C46, C62, C66, B57, B56, etc. Detailed information on each technology can be found in Supplementary Data 1.

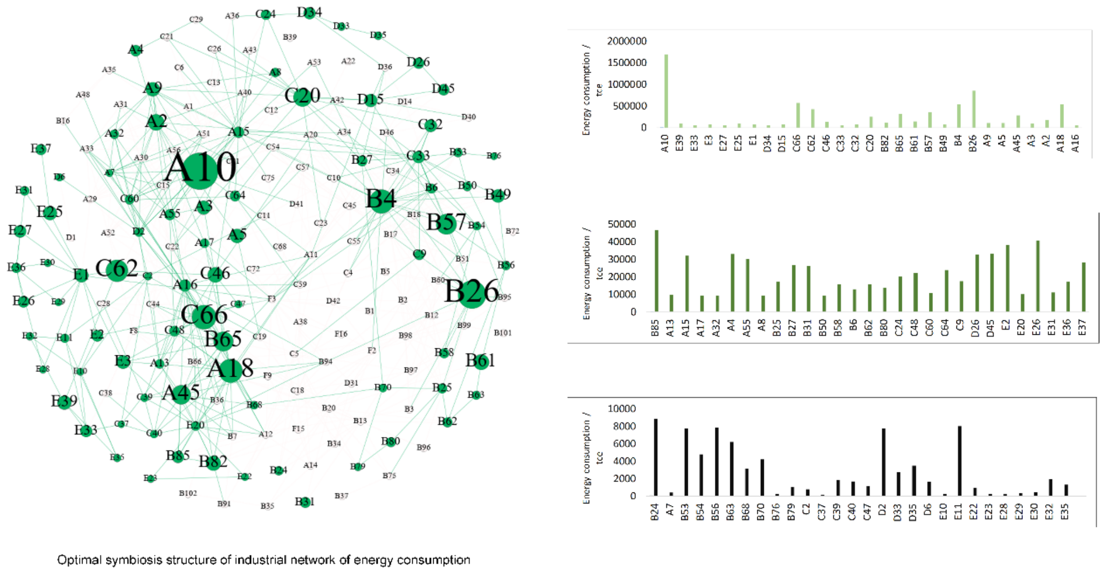

Supplementary Figure 27 Energy consumption of each node of the optimal industrial structure. The techniques with the largest energy consumption of the entire network under the optimal structure are A10, B26, B4, B57, B65, and C66. Detailed information on each technology can be found in Supplementary Data 1.

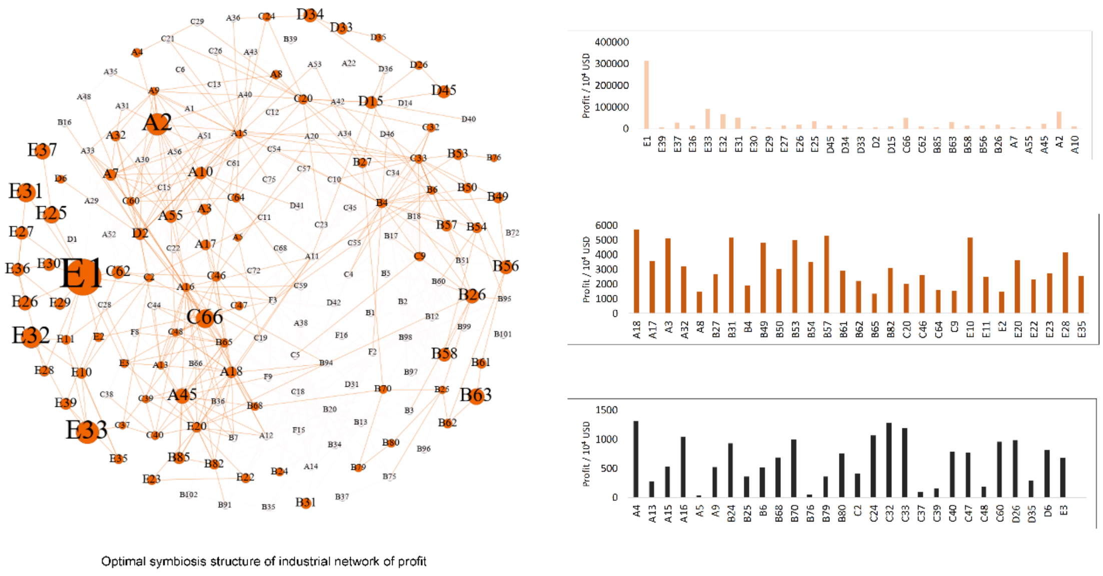

Supplementary Figure 28 Profit of each node of the optimal industrial structure. The most profitable technologies for the entire network under the optimal architecture are mainly lithium resource related technologies such as E1, E33, E32, E31, etc. For more information on each of these technologies, please refer to Supplementary Data 1.

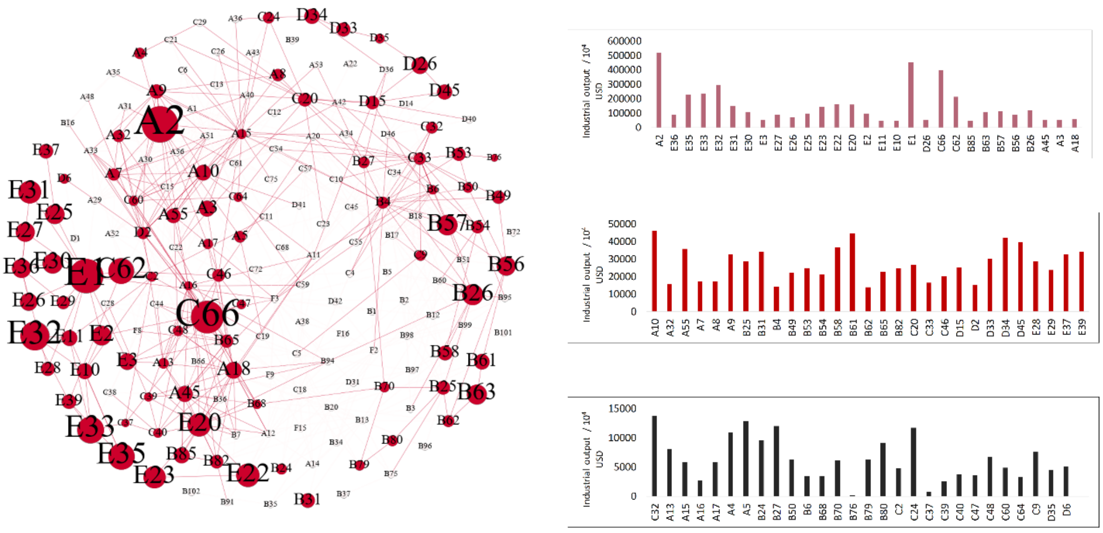

Optimal symbiosis structure of industrial network of industrial output

Supplementary Figure 29 Industrial output of each node of the optimal industrial structure. The technologies with the highest industrial output value of the whole network under the optimal structure are mainly lithium resource related technologies such as E1, E33, E32, E35, etc. as well as salt lake chemical technology A2, organic chemical technology B26, B27, etc. For details of each technology, please refer to Supplementary Data 1.

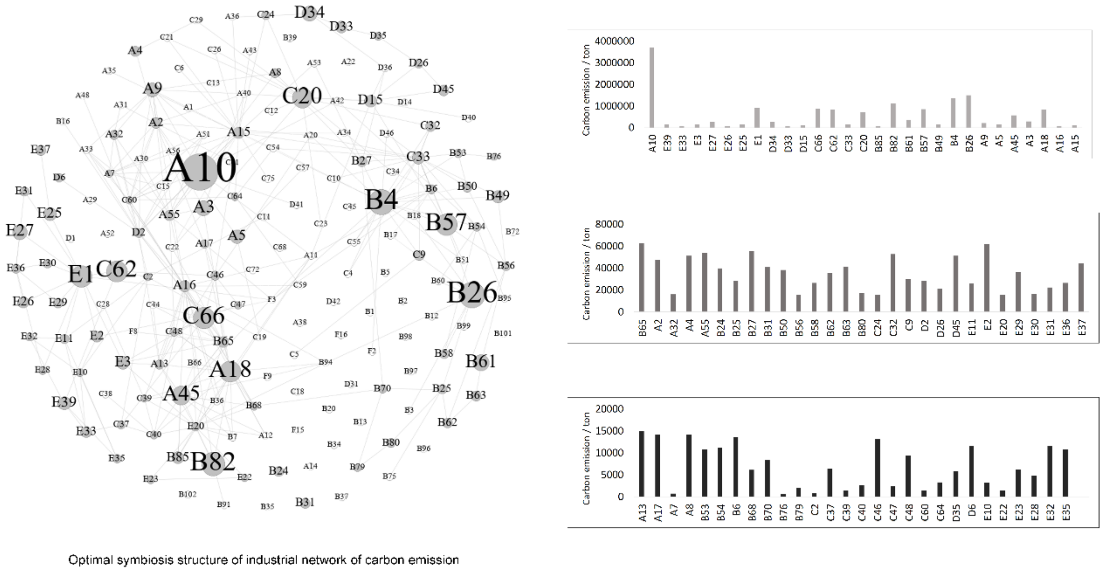

Supplementary Figure 30 Carbon emission of each node of the optimal industrial structure. The technologies with the highest carbon emissions in the entire network under the optimal structure are mainly organic chemical related technologies such as B4, B56, B26, B82, and salt lake chemical technology A10. Detailed information of each technology can be found in Supplementary Data 1.

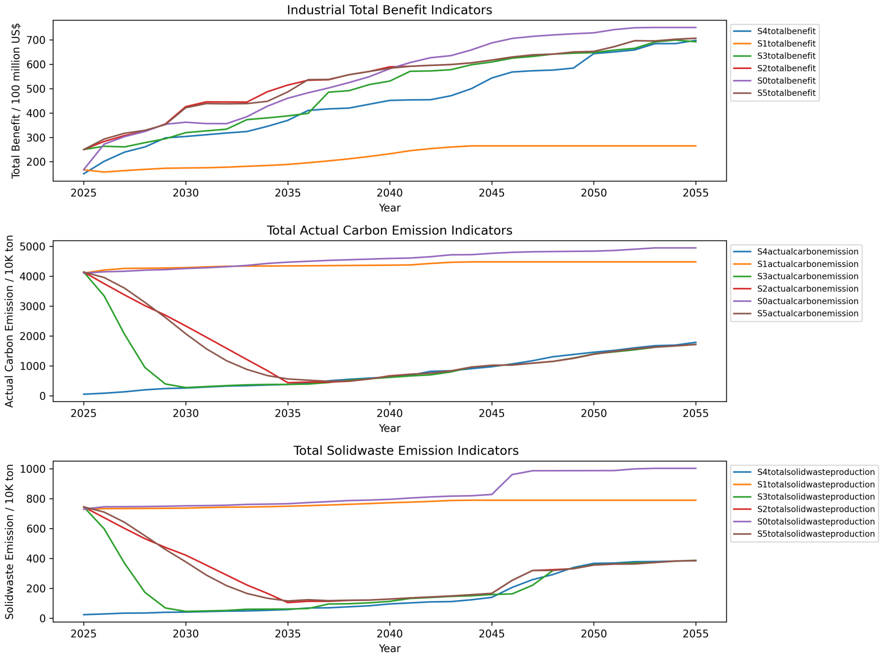

Supplementary Figure 31 Indicators of total benefit, actual carbon emission and total solid waste emission. Through the model simulation, the total benefit, actual carbon emission and total solid waste emission are obtained from 2025 to 2055, where the profit indicator is increasing in each scenario, but the S1 scenario has the smallest increase. For adjusting the original industrial structure S2, S3 and S5, the carbon emission decreases, while the carbon emission of S0 and S1 without adjusting the industrial structure increases.

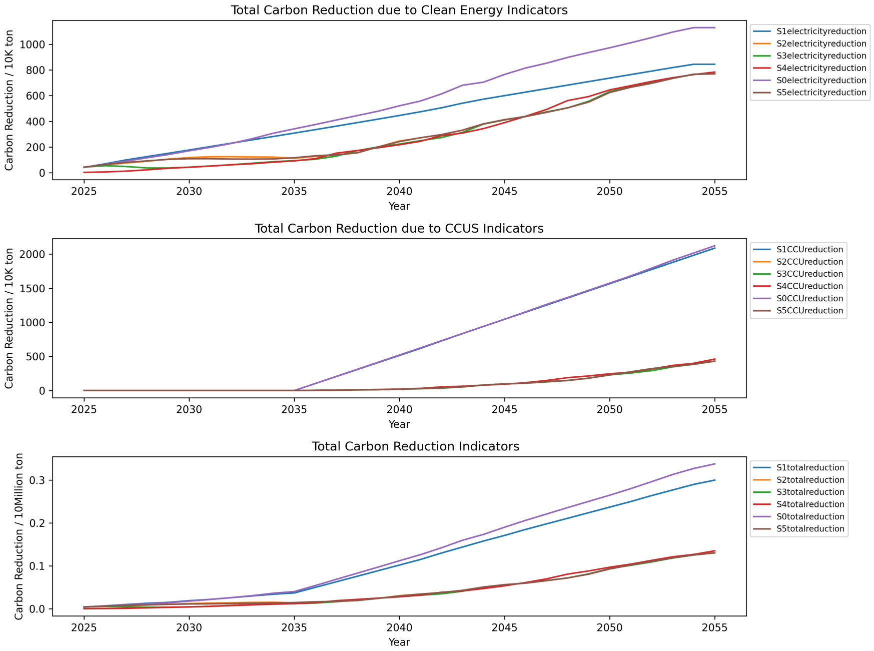

Supplementary Figure 32 Indicators of carbon reduction of clean energy, carbon capture, utilization, and storage (CCUS), and Total carbon reduction. Through the simulation of the model, the carbon emission reduction of clean energy from 2025 to 2055, the carbon emission reduction of CCUS, and the total carbon emission reduction are obtained, and the total emission reduction increases every year. S0 and S1 require more emission reductions than the other scenarios.

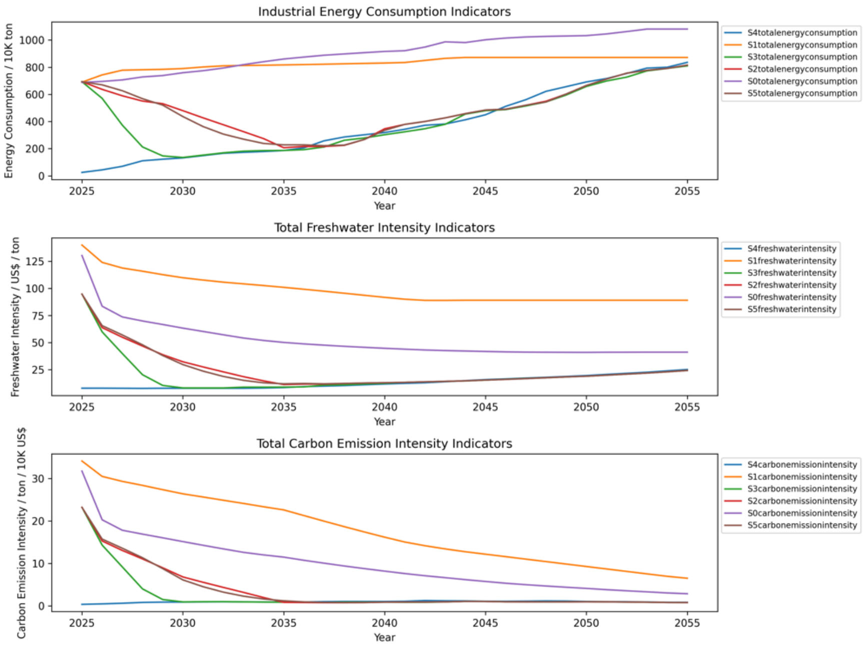

Supplementary Figure 33 Indicators of energy consumption, freshwater intensity, and carbon emission intensity. Through the simulation of the model, from 2025 to 2055energy consumption, freshwater intensity, and carbon emission intensity, for scenarios S0 and S1, energy consumption increases every year, while for scenarios S2, S3, and S5, due to the adjustment of the original industrial structure, energy consumption decreases but still increases subsequently, but the total energy consumption decreases. freshwater intensity and carbon emission intensity gradually decrease in each scenario.

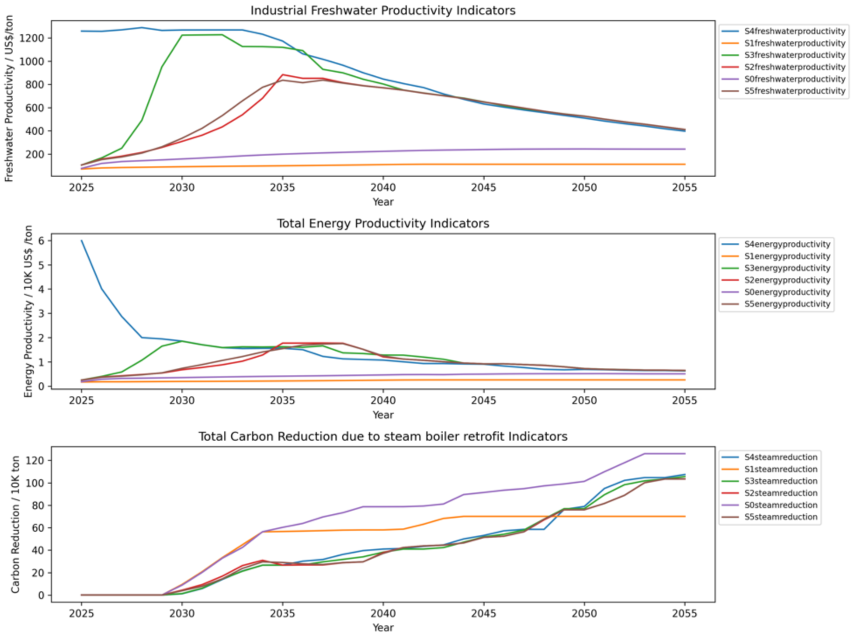

Supplementary Figure 34 Indicators of freshwater productivity, energy productivity, and carbon reduction due to steam boiler retrofit. The model simulations obtained from 2025 to 2055 freshwater productivity, energy productivity, and carbon reduction due to steam boiler retrofit. For S2, S3, and S5 scenarios freshwater productivity increases and then decreases, but for S0 and S1 scenarios freshwater productivity does not change significantly; for S4 scenarios energy productivity is higher at the beginning but then it changes insignificantly. For S2, S3, and S5, freshwater productivity increases and then decreases, but for S0 and S1, freshwater productivity does not change significantly; due to steam boiler retrofit. is increasing, but the total amount is small.

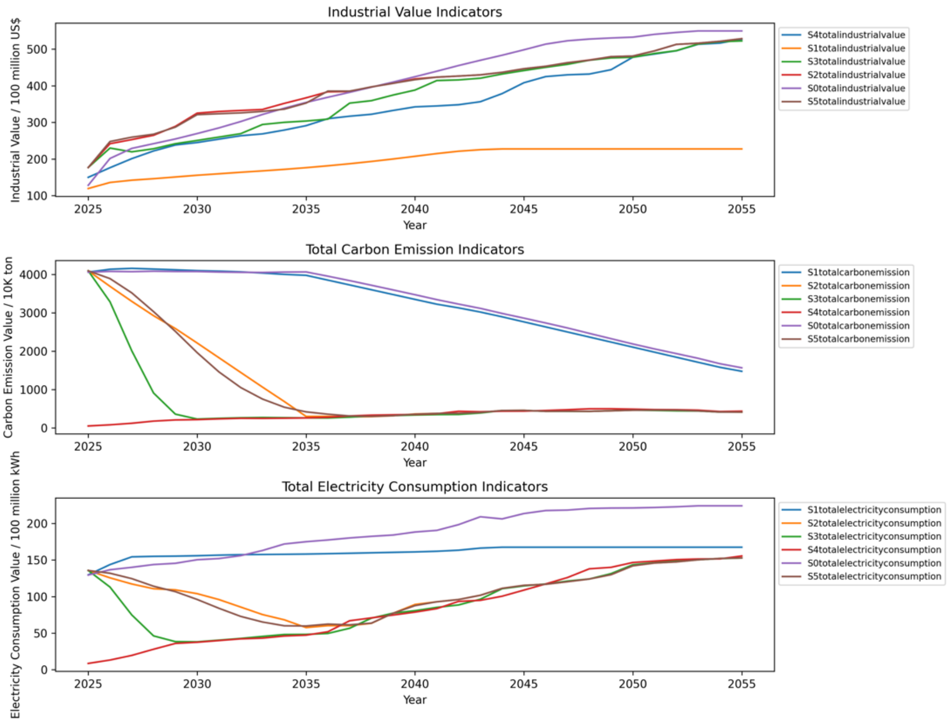

Supplementary Figure 35 Indicators of industrial value, total carbon emission, and electricity consumption. The model simulations obtained from 2025 to 2055 industrial value, total carbon emission, and electricity consumption. For industrial value, there is an increase in each of the scenarios, but the S1 scenario has the lowest increase. For S2, S3, and S5, total carbon emission and total electricity consumption decrease and then increase.

Supplementary Notes

Supplementary Note 1 Detailed information of Qinghai saline lake industrial zone

The Qinghai Salt Lake Chemical Industry Zone is situated in the Haixi Mongol and Tibetan Autonomous Prefecture within Qinghai Province, primarily encompassing the Qaidam Basin, as

shown in Supplementary Figure 36. This area is endowed with abundant resources and extensive reserves. As of 2008 statistics, the industrial zone boasts discoveries of 86 distinct mineral types, 57 of which have been confirmed as reserves. These reserves constitute approximately 28% of the nation's total and primarily consist of salt lake minerals, energy minerals, metallic minerals, and non-metallic minerals.

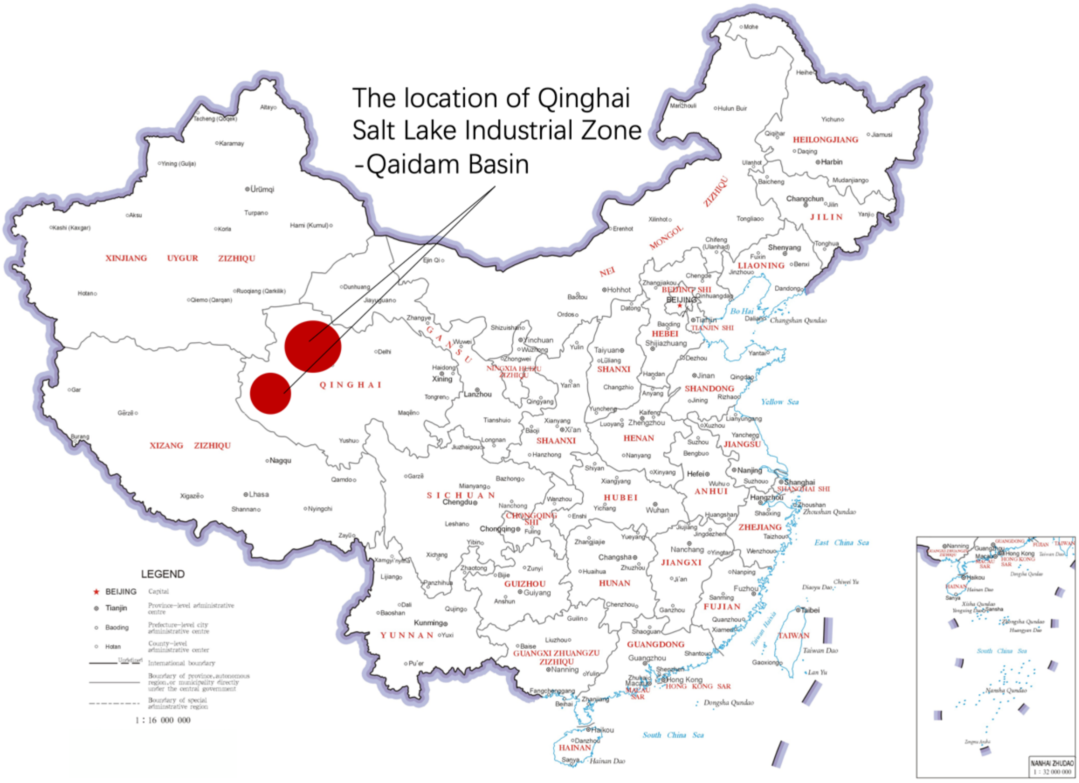

Supplementary Figure 36 The location of Qinghai Salt Lake industrial zone. The subject of this study is the Qinghai Salt Lake, located in the Qaidam Basin, Qinghai Province, China.

As of 2017, the industrial zone has been identified as having a diverse range of 111 mineral types, which constitutes approximately 82.84% of the 134 types of minerals discovered in Qinghai Province. Out of these, 90 types of minerals have been verified to have resource reserves, making up around 72% of the province's confirmed mineral reserves. Among these minerals, potash,

magnesium salt, lithium, strontium, calcium carbide tuff, alkali-production tuff, and asbestos hold the top ranking in China. Additionally, salt, manganese, bromine, and piezoelectric crystals occupy the second-ranking position in the country, while nickel, boron, talc, niobium-tantalum, and slate used for cement batching are ranked third nationally.

Supplementary Table 13 Table of Mineral Reserves in the Industrial Area as of 2017.

Category	Resource reserves/10^4 ton	National share
Potassium salt	Solid 25958.0<br>Liquid 55976.0	79.78%
Magnesium salt	Solid 1876.0<br>Liquid 53229.0	82.56%
Lithium	Liquid 154525.0	83.16%
Salt	Solid 29994064.0<br>Liquid 934194.0	22.13%
Manganese	1011675.0	9.32%
Bromine	18.0	4.18%
Piezo Crystal	29382.0	18.16%
Nickel	118.3	10.58%
Boron	Solid 858.2	26.19%
	Liquid 1228.2	
Talc	4035.0	13.16%
Niobium-tantalum	214.5	4.62%

Category	Resource reserves/10^4 ton	National share
Slate for cement batching	764.0	7.54%

As of now, the primary industrial chain in the Qinghai Salt Lake Chemical Industry Zone is centered around the comprehensive utilization of salt lake brine, with downstream deep-processing products primarily focused on five key elements: sodium, potassium, magnesium, lithium, and boron. In addition to this, the principal industries located within the industrial zone encompass coal chemistry, natural gas chemistry, petrochemicals, and metal smelting.

Supplementary Note 2 Support of carbon reduction measures

In this study, alongside the primary approach of optimizing and adjusting the industrial structure for carbon reduction, three measures that facilitate carbon emissions reduction are employed: CCUS (Carbon Capture, Utilization, and Storage)[1, 2], clean energy deployment [3, 4, 5, 6] , and steam boiler retrofitting [7] [8]. The intention is for the gradual implementation of these three measures within the industrial development path. Simultaneously, it aims to conduct a comparative assessment of the merits and drawbacks of this combination of measures against industrial structure optimization. The goal is to achieve a reduction in both direct and indirect carbon emissions within the industrial network, with direct emissions being mitigated through CCUS and indirect emissions through clean energy adoption[4].

Frequently used interchangeably with Carbon Capture, Utilization, and Storage (CCUS), Carbon Capture and Storage (CCS) projects are experiencing significant growth on a global scale in response to increasing climate objectives[9] Numerous CCS projects are underway in regions such as Europe, the Asia-Pacific, and the United States [1, 10]. In 2021, there were 135 operational commercial CCS projects, each with an average capacity of 149.4 million tons of CO_2/year[1]. Despite the current high costs associated with CCUS, its economic viability remains promising for the future[11, 12].

In this study, CCUS is primarily employed for technologies characterized by high process carbon emissions. For example, processes such as hydrogen, ammonia, and methanol production within the coal pathway are known for substantial process carbon emissions. The gradual deployment of CCUS for these technologies aims to systematically reduce their process carbon emissions[13, 14]. In the deployment of clean energy, the objective is to progressively enhance access to low-carbon electricity within the energy network. This endeavor aims to achieve a reduction in the volume of indirect carbon emissions resulting from process energy consumption. The full potential of this reduction is expected to be more evident in the future as the chemical industry undergoes full electrification[13, 15]. Additionally, in the context of steam boiler retrofitting, the gradual shift from coal-based boilers to natural gas-based ones is another strategy for reducing carbon emissions associated with the energy source. This is attributed to the fact that the carbon footprint of natural gas is approximately half that of coal[8]. The successful incorporation of these three carbon-negative measures into the model relies on alterations in the corresponding process and energy-related parameters. Future research will necessitate the exploration of other carbon-negative technologies, including those associated with CCU (Carbon Capture and Utilization).

To enhance feasibility representation, the scenarios do not rely on the full-scale application of a single measure as the foundation for carbon reduction. Instead, an incremental combination of measures is employed. This approach considers the progressive promotion and deployment of clean energy, the maturation of CCUS technology application, and steam boiler retrofitting (transitioning from coal to natural gas). The aim is to assess the cumulative impact of this combination of measures on carbon emissions.

Each scenario incorporates three measures (clean power, CCUS, and steam boiler retrofit), as shown in Supplementary Figure 19. These measures are incrementally deployed over the timelines spanning from 2030 to 2055, 2035 to 2055, and 2030 to 2055, respectively.

Incorporating clean energy assumes a progressive increase in the share of clean energy power generation over time, resulting in a gradual reduction in its associated carbon footprint. This inclusion of a time-series change within the model is designed to simulate the gradual maturation of clean energy technology and its influence on the low-carbon transformation of industrial zones. While the integration of clean energy power generation can effectively reduce the carbon footprint of electricity usage, it's worth noting that the future maturity of clean energy technology, its stability, and the extent of electrification may impact the model's outcomes.

Incorporating CCUS (Carbon Capture, Utilization, and Storage), it is anticipated that this approach will reduce direct carbon emissions from technological processes. However, it's important to note that the current maturity of CCUS deployments is limited, and the technology carries a high cost, coupled with an uncertain cost range[1, 10].

In this study, the approach used to simulate the cost of CCUS (Carbon Capture, Utilization, and Storage) involves considering a cost range and assuming a gradual deployment maturity.

Specifically, the CCUS carbon reduction cost is directly matched with the generation of process carbon emissions. This allows for the calculation of the overall CCUS carbon reduction cost. Regarding the cost of CCUS, it is noteworthy that the current uniform cost of CCUS has not stabilized, and there is still significant room for cost reduction. As a result, we choose to explore the potential cost of carbon reduction within a fluctuation range of 15-53 USD/ton CO_2[1, 10]. The focus of this analysis is on the ratio of expenditure allocated to CCUS (Carbon Capture, Utilization, and Storage) carbon reduction to the total profit generated by the overall industrial network. It becomes apparent that in the S1 scenario, carbon reduction through CCUS accounts for a minimum of approximately 10-25% of the overall industrial network's profit. The exact percentage is influenced by specific CCUS costs, which are generally higher. Furthermore, examining the optimization results reveals that steam boiler retrofit contributes the least to carbon reduction across different pathways. In S0 and S1, the carbon reduction achieved by CCUS is on par with that of clean energy. This is primarily because S0 and S1 retain the original industrial structure characterized by high direct carbon emissions, necessitating assistance from CCUS to achieve substantial carbon emission reductions. Conversely, in S2-S5, where the original inefficient industrial structure is less restrictive, more carbon emissions result from energy consumption. As a result, the contribution of clean energy to carbon reduction is more significant.

Supplementary Note 3 Sensitive analysis

In terms of parameter selection, we have chosen representative parameters from the model's input, coefficient, and output parameters. These parameters include saline brine supply, Li_2CO_3 price, Li_2CO_3 upper limit, Potash fertilizer upper limit, K_2CO_3 upper limit, Si supply, Coal supply,

and K_2CO_3 upper limit. In total, seven parameters were selected for analysis. We explored the impact of fluctuations in each parameter at various levels, ranging from ±5%, ±10%, ±20%, ±50%, ±80%. As illustrated in Supplementary Figure 37 and Supplementary Figure 38, this visualization demonstrates the effect of a ±50% change on the model using sensitivity coefficients, which depict the relative sensitivity of each parameter to the overall model.

$$Sensitivity\ coefficient = \frac{\frac{\Delta Index}{Index}}{\frac{\Delta para}{Para}} \tag{76}$$

The sensitivity coefficient quantifies the model's responsiveness to variations in different parameters when small alterations in the model's parameters impact the model's output, as shown in Equation (76).

Specifically, we focused on three key input parameters within the model during the parameter selection process. These parameters include saline lake brine, coal, and silicon metal. Among them, saline lake brine, serving as the fundamental raw material within the entire industrial network, possesses the potential for significant repercussions on the overall model if subjected to alterations. Our findings indicate that changes in the supply of saline lake brine are closely linked to numerous output indicators within the model.

Coal and silicon metal also stand out as primary inputs to the network. Unlike other substances, these materials cannot be internally generated by nodes within the network; they necessitate external supply sources. In addition to these inputs, we considered the price fluctuations of lithium carbonate. This inclusion enables us to simulate market dynamics. Within the model, lithium carbonate represents a pivotal high-value product and serves as the primary raw material for subsequent stages of deep processing within the lithium industry. Therefore, any fluctuations in the price of lithium carbonate and the associated uncertainties merit significant attention in the model.

Sensitivity coefficient	IV	CE	FC	ELEC	SDP	Pro	ENC	WW	FWI	CEI	EI	CCI	SDPI	FWP
Saline brine supply	14.97%	−24.77%	6.96%	−9.53%	11.93%	20.742%	−3.77%	6.86%	−9.42%	−46.73%	−28.81%	−22.03%	−3.57%	8.61%
Li_2CO_3 price	8.83%	−0.26%	0.31%	0.04%	0.45%	2.748%	−0.06%	0.31%	−8.56%	−9.16%	−8.85%	−8.95%	−8.42%	8.53%
Li_2CO_3 upper limit	3.91%	−2.65%	2.19%	0.29%	3.60%	5.857%	−0.73%	2.16%	−1.80%	−6.83%	−3.77%	−4.83%	−0.32%	1.77%
Potash fertilizer upper limit	14.77%	−23.41%	7.02%	−8.86%	11.78%	20.269%	−3.60%	6.93%	−9.09%	−44.79%	−27.73%	−21.55%	−3.50%	8.33%
K_2CO_3 upper limit	−0.13%	−1.62%	−0.85%	−0.52%	−0.01%	-0.112%	−0.49%	−0.84%	−0.72%	−1.49%	−0.39%	−0.36%	0.12%	0.71%
Si supply	1.85%	−15.78%	−4.87%	−4.18%	−0.07%	0.427%	−1.02%	−4.81%	−6.85%	−17.96%	−6.14%	−2.92%	−1.96%	6.41%
Coal supply	0.95%	24.91%	4.64%	19.24%	−4.63%	1.909%	0.49%	4.57%	3.66%	23.74%	18.12%	−0.45%	−5.53%	−3.53%

IV	Industrial output	SDP	Solidwaste production	FWI	Freshwater intensity	SDPI	Solid intensity
CE	Carbon emission	Pro	Profit	CEI	Carbon emission intensity		
FC	Freshwater consumption	ENC	Energy consumption	EI	Energy intensity	FWP	Water productivity
ELEC	Electricity consumption	WW	Waste water	CCI	Coal consumption intensity		

Supplementary Figure 37 Sensitive analysis of parameters. The sensitivity analysis takes into account the impact of fluctuations in the supply of brine from salt lakes, lithium carbonate price, lithium carbonate demand upper limit, potash demand upper limit, potassium carbonate demand ceiling, silicon supply, coal supply, and other factors on different indicators, such as industrial output (IV), carbon emission (CE), freshwater consumption (FC), electricity consumption (ELEC), solid waste production (SDP), profit (Pro), energy consumption (ENC), wastewater (WW), freshwater intensity (FWI), carbon emission intensity (CEI), energy intensity (EI), coal consumption intensity (CCI), solid intensity (SDPI), water productivity (FWP).

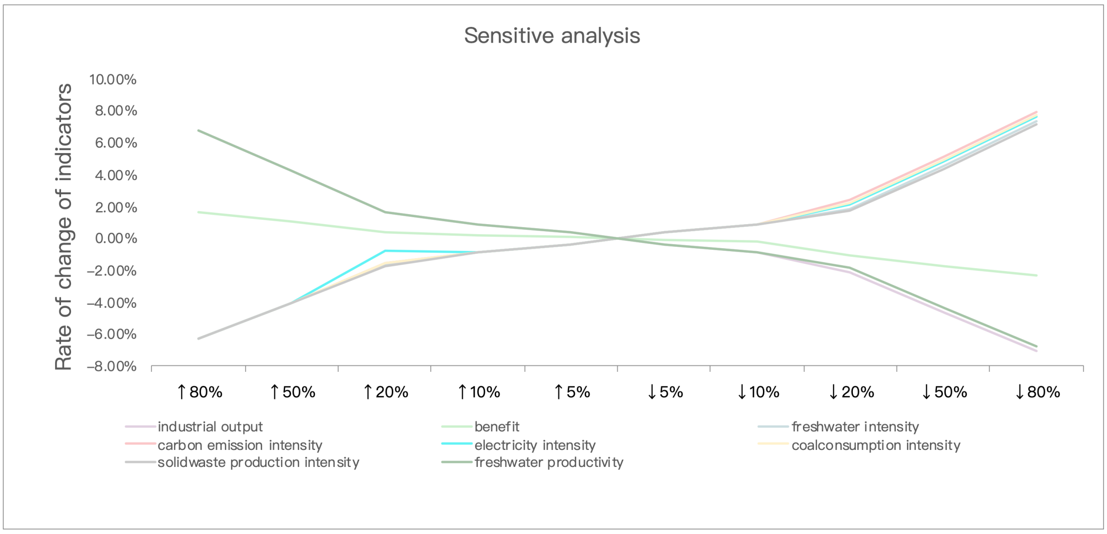

Supplementary Figure 38 Sensitive analysis of Li_2CO_3 price fluctuation (±5%, ±10%, ±20%, ±50%, ±80%). The sensitivity analysis considers the impact of lithium carbonate price fluctuations of ±5%, ±10%, ±20%, ±50%, and ±80% on different indicators such as industrial output, carbon emission intensity, solid waste production intensity, benefit, electricity intensity, freshwater

productivity, freshwater intensity, coal consumption intensity, electricity intensity, freshwater productivity, freshwater intensity, and coal consumption intensity.

Supplementary Discussions

Supplementary Discussion 1 Optimal paths and industrial symbiosis times for RRIEDOM decision making

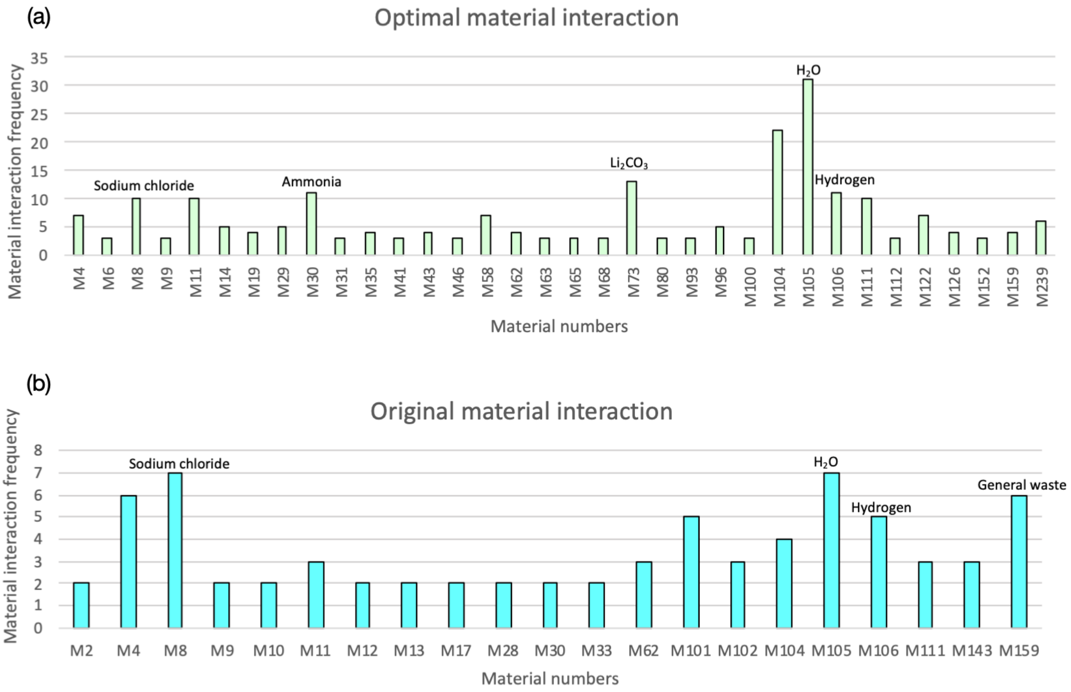

Supplementary Figure 39 Material symbiosis times of the original structure, and the optimal structure. The material interactions in the original and optimal structures are analyzed, and the material interactions in the optimal structure (a) have a complex diversity compared with the original structure (b). For example, the material symbiosis times of Li_2CO_3 in the optimal structure is 13, indicating that there are 13 exchanges of the input and output nodes occurring owing to

Li_2CO_3-related technologies in the optimal structure.

By analyzing the frequency of material symbiosis, it is concluded that the overall frequency of material exchange in the network increases after optimization. This observation, indicates an increased robustness of the network, which increases the resilience of the industrial network against risks.

Supplementary Figure 39 provides a comprehensive representation of material symbiosis and its frequency within the optimal industrial structure. The initial configuration of the entire industrial network includes nearly 250 types of materials, including raw materials, products, transformation intermediates, and wastes. These materials interact with nodes to form an extensive superstructure network. After optimizing the mathematical model, the exchange frequency of materials within the resulting network can be analyzed.

The analysis shows that the exchange frequency of materials generally increases before and after network optimization, with materials such as H_2O, NH_3, and NaCl showing elevated exchange rates. Of particular note is Li_2CO_3, which has the highest growth rate. This can be attributed to the increase in the proportion of the lithium resource deep-processing industry in the overall industrial structure after optimization. As the main product of lithium extraction from saline lakes and the key raw material for the subsequent lithium battery processing technology, Li_2CO_3 occupies an important position in the network.

Simultaneously, an increase in the symbiosis frequency of NaCl is observed, although its overall ratio is decreasing. This implies that while the technology industry associated with NaCl evolves post-optimization, its importance within the overarching industry diminishes.

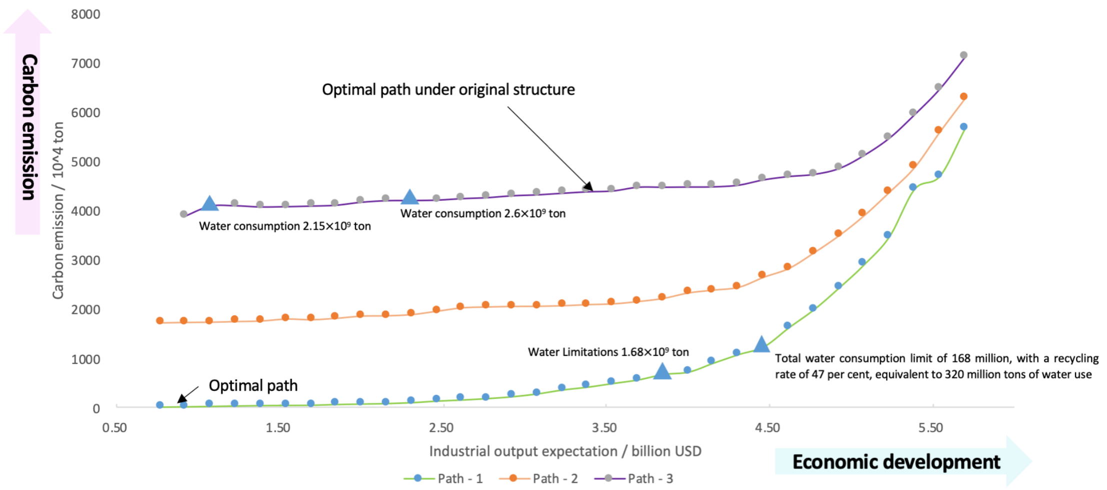

Supplementary Figure 40 Original development path and optimal development path of saline lake industries. Three paths were designed in order to explore the properties of the different development paths. Path - 1 (purple solid line): Initial structure of the industrial area as a starting point, followed by the resource-based regional industrial economy development optimization model (RRIEDOM) modeling to guide development. Path - 2(orange solid line): Starting point with PVC demand of 1 million tons, followed by RRIEDOM modeling. Path - 3 (green solid line): No initial structure setup, direct development using RRIEDOM modeling.

Supplementary Figure 40 presents notable results that illustrate the optimal simulation under each economic objective, namely the lowest environmental cost or material resource cost. Depending on the vertical coordinate parameter of interest, all environmental impacts, such as carbon emissions and water depletion, can be summarized and converted into a material resource cost. This can be done by weighting and simulating the industrial network structure under the prevailing economic objective through the use of RRIEDOM.

Path-3, which starts the simulation without any local initial industry in the setup area and takes a biennial step size (which only affects the resolution), shows a development curve. This curve

corresponds to the minimum material resource cost for each development objective and is identified as the optimal development curve or path. Each identified economic objective on the optimal development path represents the minimum material resource cost under that objective. Therefore, a regional entity seeking to achieve a particular economic objective cannot have a material resource cost lower than the cost under the optimal development path. Thus, the optimal development path represents the minimum material resource cost for the region's economic objective.

Under this premise, the closer a region's development curve is to the locally desired optimal development curve, the more optimal the region's development can be considered in a historically integrated perspective. This conclusion accounts for potentially lower material resource costs given the same economic goals.

In the meantime, the network is programmed to produce at least 1 million tons of PVC resin product via either the calcium carbide or ethylene path (Path-2). The same optimization strategy as described above is used, and this process derives the Path-2. This approach aims to explore the potential shape of regional development under the constraint of a fixed network minimum output product volume. It can be observed that with a fixed product demand, the network still experiences a higher material resource cost when the economic objective is lower compared to the initial no-demand scenario.

When the regional network has additional demand, it is difficult to reduce the material resource cost of the industrial network solely by optimizing and adjusting the industrial network structure. However, during the subsequent network development, it is still possible to maintain a development path similar to the optimal development path, which can be attributed to the foresighted planning guidance of RRIEDOM.

At the same time, the optimal development path is simulated under the initial industrial structure, represented by Path-1, or the optimal development path with higher additional demand costs. Under the optimal development path of the industrial zone, the water consumption reaches the limit of 0.168 billion tons/year at about 240 billion RMB. Meanwhile, under the optimal development path with the initial industrial structure, the region requires consumption of 0.215 billion tons/year at about 75 billion RMB. This suggests that if the initial industrial structure is missing, the region will face higher water demand.

In essence, advocating a development pattern closer to the optimal development path can facilitate the region's progress toward long-term economic goals while alleviating environmental pressures.

Regarding how the model determines what products to produce, in what quantities, and the methods to produce them for a given region

In traditional human decision-making processes, such as expert-driven determinations of regional product selection and production volumes, considerations are primarily based on local factors like available resources, market capacity, pricing dynamics, and profitability. Typically, well-established technologies are associated with certain empirical parameters, such as economically viable production scales. The choice of production methods often hinges upon the local availability of viable technological pathways and the maturity level of these technologies.

Within the framework of this research model, aspects that could only be roughly assessed through human intervention are quantitatively delineated and scrutinized with greater precision. It is worth noting that the fundamental distinction lies in the fact that conventional human decision-making is predominantly capable of addressing straightforward aspects, such as basic resource-

product combinations. However, it faces significant limitations when confronted with multifaceted considerations encompassing multiple resources, products, technologies, and extended time horizons.

In contrast, this research model possesses a degree of complexity that enables it to address scenarios that extend far beyond the capabilities of human decision-makers. Moreover, it facilitates the explicit consideration of intricate interrelationships among various factors and allows for the assessment of coupled effects and interactions that are often challenging for human decision-makers to discern and incorporate into their deliberations.

In further detail, the initial phase of this research involved conducting assessments of local resource endowments through comprehensive resource surveys and on-site investigations. It is essential to emphasize that this approach applies not only to the area under study but also extends to other regions with similar characteristics. The rationale behind this strategy is rooted in the principle of initiating industrial value chains based on the exploitation of predominant local resources.

Subsequently, starting from the compilation of resource endowments, this research delved into an examination of potential technologies for resource utilization. This stage was instrumental in establishing a comprehensive technical database. Within the context of process industries, this framework considered the entire spectrum from upstream raw material processing to downstream precision processing.

It is paramount to recognize that these initial phases have effectively laid the groundwork for outlining potential development directions. Importantly, these directions encompass a comprehensive array of possible resource utilization technology combinations, often referred to as

industrial structures. This approach significantly contrasts with conventional expert-driven decision-making processes.

Following these preparatory phases, the model then employs mathematical optimization methods to determine the optimal industrial structure from within the set of technologies assembled. This also includes an evaluation of the appropriate scales for these technologies. The universality, applicability, and transferability of this approach make it suitable for resource-rich regions across various geographical contexts. Moreover, it is imperative to underscore that the establishment of the technology database adheres to the principles of comprehensiveness and reliability to ensure the decisions solid and instructive.

Regarding the industrial symbiosis in the optimal network structure

In this study, the optimized integrated industrial network has significantly improved industrial symbiosis. For instance, coupling the techniques of potash extraction from salt lakes with lithium extraction from the same source has increased the overall efficiency of lithium resource utilization from salt lakes. Traditional lithium extraction methods directly from salt lakes are associated with high purification costs due to low lithium ion concentrations and high impurity levels. However, through the coupling of potash extraction with lithium extraction from old brine, a byproduct of potash extraction, can be directly utilized for lithium extraction. This synergistic coupling of the two techniques has reduced the overall production costs. Furthermore, potash extraction from salt lakes generates another byproduct, magnesium chloride hexahydrate, also called bischofite, which typically contains low levels of valuable elements. Traditionally, these minerals are stockpiled, but within the network, these minerals can be coupled with magnesium oxide production and other technologies, enhancing resource efficiency and reducing resource consumption. Currently, a

production line with an annual capacity of 30,000 tons of electro-fused magnesium oxide has been established in the industrial area, equivalent to the utilization of 400,000 tons of magnesium chloride hexahydrate annually.

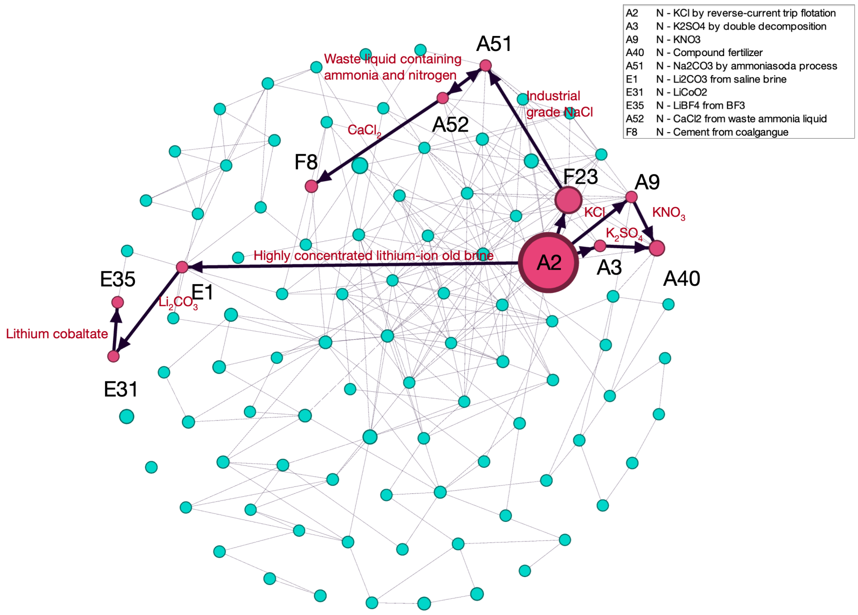

Supplementary Figure 41 Analysis of the industry chains for industrial network symbiosis.

By parsing the optimized results, different industrial chains in the industrial network superstructure can be obtained, for example, A2 technology is the production of potassium chloride, and its by-products of lithium-containing brine can be used for lithium extraction from salt lakes technology (E1) as raw materials, and E1 is used for the production of lithium cobaltate in E31 after lithium carbonate is extracted from the lithium-containing brine.

Additionally, potash extraction from salt lakes generates waste tailings containing relatively high concentrations of NaCl. However, due to the high impurity content, these tailings are commonly stockpiled. In the optimized network, these tailings are efficiently utilized through a

utilization node, where they can be processed into industrial or food-grade NaCl. Presently, an annual production capacity of 7,000,000 tons of potash tailings processing has been established in the industrial zone, enhancing resource utilization efficiency and reducing environmental impacts. Supplementary Figure 41 illustrates specific industrial chains within the optimal industrial structure, highlighting the interrelationships and synergies among different technologies. In summary, complex industrial networks can offer diverse symbiotic systems that are challenging for human decision-making to consider comprehensively.

Research comparison

We compared the studies related to this study and summarized the similarities and differences between the different studies as shown in Supplementary Table 14.

Supplementary Table 14 Comparison of related research.

	Other studies	This study
Material types	Water[16, 17]	Involving water, energy and more than 250 types of material interactions
	Energy[18, 19 20]	
	Few material interactions[21, 22, 23, 24]	
Complexity of industrial network	No more than 50 nodes[15, 21, 25, 26]	Complex interactions of over 180 technologies
Decarbonization	Low-carbon technologies[2, 5, 13, 15]	Low-carbon technologies and industrial structure improvement

Evolutionary path		Optimal path evolution on long time scales
Practical significance	Regional industrial planning adjustments	Regional industrial planning guidance

Supplementary Discussion 2 Data description

To substantiate the research presented in this article, we required a substantial volume of technical data. We collected detailed technical databases from diverse sources, including the Intergovernmental Panel on Climate Change (IPCC)[27], China's National Bureau of Statistics[28], China's industry standards[29], government-industry reports[30, 31], park industry reports[31], and project environmental impact assessment documents, among others. In Supplementary Data 1, we have provided each data type.

Raw data encompasses both text data and technological process data.

Text data comprises information such as the technology's nomenclature, industrial category, primary input raw materials, principal output products, and any associated by-products. Technological process data encompasses various parameters, including unit energy consumption, water consumption, electricity consumption, solid waste emissions, carbon emissions, wastewater emissions, investment costs, profit margins, industrial output values, and more, specific to each technology and standardized according to the principal products.

The textual data primarily serves the purpose of constructing the industrial network

superstructure, a term in systems engineering that denotes a topological framework encompassing a range of potential solutions to a given problem. By amalgamating textual information related to various technologies, we can extract the superstructure governing interactions between each technology.

Conversely, the process data associated with technologies primarily function as mathematical optimization parameters and constraints for each node within the network. These constraints encompass variables such as water consumption, energy consumption, and carbon emissions, among others. Mathematical optimization aims to attain the desired quantitative network structure as per the research objectives.

The development of the technology list evolves progressively during the establishment of the industrial node information database. The initial focus is on comprehensive resource utilization technologies associated with salt lakes, encompassing technologies for extracting and utilizing resources such as brine, coal, oil, natural gas, and various mineral resources. The primary products resulting from these technologies serve as a foundational framework for further research, thereby facilitating the expansion of data within the information repository. These technologies undergo a rigorous screening process, ensuring their readiness for industrialization or large-scale industrial demonstration. This comprehensive collection effort has culminated in the identification of an extensive node information repository, comprising textual details concerning the inputs and outputs of these technologies, as well as their associated industrial categories. This repository encompasses an extensive inventory, numbering close to 358 technologies.

Supplementary References

1. Hong WY. A techno-economic review on carbon capture, utilisation and storage systems for achieving a net-zero CO2 emissions future. *Carbon Capture Science & Technology* **3**, 100044 (2022).

2. Hasan MF, First EL, Boukouvala F, Floudas CA. A multi-scale framework for CO2 capture, utilization, and sequestration: CCUS and CCU. *Computers & Chemical Engineering* **81**, 2-21 (2015).

3. Pehl M, Arvesen A, Humpenöder F, Popp A, Hertwich EG, Luderer G. Understanding future emissions from low-carbon power systems by integration of life-cycle assessment and integrated energy modelling. *Nature Energy* **2**, 939-945 (2017).

4. Jacobson MZ. Review of solutions to global warming, air pollution, and energy security. *Energy & Environmental Science* **2**, 148-173 (2009).

5. Kittner N, Lill F, Kammen DM. Energy storage deployment and innovation for the clean energy transition. *Nature Energy* **2**, 1-6 (2017).

6. Chu S, Majumdar A. Opportunities and challenges for a sustainable energy future. *nature* **488**, 294-303 (2012).

7. Zheng C, Wu X, Chen X. Low-carbon transformation of ethylene production system through deployment of carbon capture, utilization, storage and renewable energy technologies. *Journal of Cleaner Production* **413**, 137475 (2023).

8. Mahmoud A, Sunarso J. A comparative study on targeting CO2 emissions reduction from small-scale utility system.). 1 edn. IOP Publishing.

9. IEA. About CCUS (Available from: https://www.iea.org/reports/about-ccus, Accessed 30 August 2023) (2021).

10. Edwards RW, Celia MA. Infrastructure to enable deployment of carbon capture, utilization, and storage in the United States. *Proceedings of the National Academy of Sciences* **115**, E8815-E8824 (2018).

11. Wei Y-M*, et al.* A proposed global layout of carbon capture and storage in line with a 2 C climate target. *Nature Climate Change* **11**, 112-118 (2021).

12. Hasan MF, Boukouvala F, First EL, Floudas CA. Nationwide, regional, and statewide CO2 capture, utilization, and sequestration supply chain network optimization. *Industrial & Engineering Chemistry Research* **53**, 7489-7506 (2014).

13. Khan MHA, Daiyan R, Neal P, Haque N, MacGill I, Amal R. A framework for assessing economics of blue hydrogen production from steam methane reforming using carbon capture storage & utilisation. *International Journal of Hydrogen Energy* **46**, 22685-22706 (2021).

14. Hosseini SE, Wahid MA. Hydrogen production from renewable and sustainable energy resources: Promising green energy carrier for clean development. *Renewable and Sustainable Energy Reviews* **57**, 850-866 (2016).

15. Kätelhön A, Meys R, Deutz S, Suh S, Bardow A. Climate change mitigation potential of carbon capture and utilization in the chemical industry. *Proceedings of the National Academy of Sciences* **116**, 11187-11194 (2019).

16. Lee J-Y, Chen C-L, Lin C-Y, Foo DCY. A two-stage approach for the synthesis of inter-plant water networks involving continuous and batch units. *Chemical Engineering Research and Design* **92**, 941-953 (2014).

17. Leong YT, Lee J-Y, Chew IML. Incorporating timesharing scheme in ecoindustrial multiperiod chilled and cooling water network design. *Industrial & Engineering Chemistry Research* **55**, 197-209 (2016).

18. Chae SH, Kim SH, Yoon S-G, Park S. Optimization of a waste heat utilization network in an eco-industrial park. *Applied Energy* **87**, 1978-1988 (2010).

19. Liew PY*, et al.* Total Site Heat Integration planning and design for industrial, urban and renewable systems. *Renewable and Sustainable Energy Reviews* **68**, 964-985 (2017).

20. Ramos M, Boix M, Aussel D, Montastruc L, Domenech S. Optimal design of water exchanges in eco-industrial parks through a game theory approach. In: *Computer Aided Chemical Engineering*). Elsevier (2016).

21. Cimren E, Fiksel J, Posner ME, Sikdar K. Material flow optimization in by-product synergy networks. *Journal of Industrial Ecology* **15**, 315-332 (2011).

22. Kantor I, Elkamel A, Fowler MW. Optimisation of material and energy exchange in an eco-park network considering three fuel sources. *International Journal of Advanced Operations Management* **6**, 285-308 (2014).

23. Kantor I, Fowler M, Elkamel A. Optimized production of hydrogen in an eco-park network accounting for life-cycle emissions and profit. *international journal of hydrogen energy* **37**, 5347-5359 (2012).

24. Mamoune A, Yassine A. Creating an inductive model of industrial development with

optimized flows for reducing its environmental impacts. *Energy Procedia* **6**, 396-403 (2011).

25. Fan J, Hu S, Chen D, Zhou Y. Study on the construction and optimization of a resource-based industrial ecosystem. *Resources, Conservation and Recycling* **119**, 97-108 (2017).

26. Zhu Q, Hu S. Improved interactive inference approach for constructing a complex multi-industrial symbiosis network. *Environmental Science and Pollution Research* **28**, 55401-55418 (2021).

27. IPCC. IPCC data (https://www.ipcc.ch/data/).) (2023).

28. (CNBS) CNBoS. China Statistical Yearbook (http://www.stats.gov.cn/sj/ndsj/).) (2023).

29. Platform CSIPS. industry standard (https://std.samr.gov.cn/hb).) (2023).

30. Zone TPCE. Haixi Mineral Reserves Summary Table (http://cdm.qinghai.gov.cn/).) (2023).

31. Zone TPCE. Golmud Industrial Park Industrial Development Plan (http://cdm.qinghai.gov.cn/).) (2018).
