# Supplementary material for: Integrated optimization modelling framework for low-carbon and green regional transitions through resource-based industrial symbiosis
Source: Nat Commun. 2024 May 7;15:3842. doi: 10.1038/s41467-024-48249-6 (PMC11076570; doi:10.1038/s41467-024-48249-6)
Supplement: Supplementary file 2 — Description of Additional Supplementary Files [file 41467_2024_48249_MOESM2_ESM.pdf]

<u>Description of Additional Supplementary Files</u>

Supplementary Data Legend:

Supplementary Data 1: The supplemental data file provides information in a total of seven sections, namely: Node database; SNA information; Input output matrix; Materials constraints; Nodes limitations; Data of sensitivity analysis; Scenarios results.
