# Supplementary material for: Integrated optimization modelling framework for low-carbon and green regional transitions through resource-based industrial symbiosis
Source: Nat Commun. 2024 May 7;15:3842. doi: 10.1038/s41467-024-48249-6 (PMC11076570; doi:10.1038/s41467-024-48249-6)
Supplement: Supplementary file 4 — Reporting Summary [file 41467_2024_48249_MOESM4_ESM.pdf]

nature portfolio

Corresponding author(s): NCOMMS-23-18882

Last updated by author(s): Apr 12, 2024

Reporting Summary

Nature Portfolio wishes to improve the reproducibility of the work that we publish. This form provides structure for consistency and transparency in reporting. For further information on Nature Portfolio policies, see our Editorial Policies and the Editorial Policy Checklist.

Statistics

For all statistical analyses, confirm that the following items are present in the figure legend, table legend, main text, or Methods section.

n/a	Confirmed	
☒	☐	The exact sample size (n) for each experimental group/condition, given as a discrete number and unit of measurement
☒	☐	A statement on whether measurements were taken from distinct samples or whether the same sample was measured repeatedly
☒	☐	The statistical test(s) used AND whether they are one- or two-sided<br>*Only common tests should be described solely by name; describe more complex techniques in the Methods section.*
☒	☐	A description of all covariates tested
☒	☐	A description of any assumptions or corrections, such as tests of normality and adjustment for multiple comparisons
☒	☐	A full description of the statistical parameters including central tendency (e.g. means) or other basic estimates (e.g. regression coefficient) AND variation (e.g. standard deviation) or associated estimates of uncertainty (e.g. confidence intervals)
☒	☐	For null hypothesis testing, the test statistic (e.g. F, t, r) with confidence intervals, effect sizes, degrees of freedom and P value noted<br>*Give P values as exact values whenever suitable.*
☒	☐	For Bayesian analysis, information on the choice of priors and Markov chain Monte Carlo settings
☒	☐	For hierarchical and complex designs, identification of the appropriate level for tests and full reporting of outcomes
☒	☐	Estimates of effect sizes (e.g. Cohen's d, Pearson's r), indicating how they were calculated

Our web collection on statistics for biologists contains articles on many of the points above.

Software and code

Policy information about availability of computer code

Data collection	Not applicable in this study.
Data analysis	Not applicable in this study.

For manuscripts utilizing custom algorithms or software that are central to the research but not yet described in published literature, software must be made available to editors and reviewers. We strongly encourage code deposition in a community repository (e.g. GitHub). See the Nature Portfolio guidelines for submitting code & software for further information.

Data

Policy information about availability of data

All manuscripts must include a data availability statement. This statement should provide the following information, where applicable:

- Accession codes, unique identifiers, or web links for publicly available datasets
- A description of any restrictions on data availability
- For clinical datasets or third party data, please ensure that the statement adheres to our policy

Source data are provided with this paper. Other data supporting the main findings of this study and the important parameters used in the modeling analysis can be found in the Supplementary Information and the Supplementary Data files.

Research involving human participants, their data, or biological material

Policy information about studies with human participants or human data. See also policy information about sex, gender (identity/presentation), and sexual orientation and race, ethnicity and racism.

Reporting on sex and gender	Not applicable in this study.
Reporting on race, ethnicity, or other socially relevant groupings	Not applicable in this study.
Population characteristics	Not applicable in this study.
Recruitment	Not applicable in this study.
Ethics oversight	Not applicable in this study.

Note that full information on the approval of the study protocol must also be provided in the manuscript.

Field-specific reporting

Please select the one below that is the best fit for your research. If you are not sure, read the appropriate sections before making your selection.

☐ Life sciences ☐ Behavioural & social sciences ☒ Ecological, evolutionary & environmental sciences

For a reference copy of the document with all sections, see nature.com/documents/nr-reporting-summary-flat.pdf

Ecological, evolutionary & environmental sciences study design

All studies must disclose on these points even when the disclosure is negative.

Study description	The development and utilization of bulk resources provide the basic material needs for industrial systems. However, the majority of the current resource utilization patterns are unsustainable. The pattern of the regional industrial area is unsustainable with low efficiencies, high carbon emissions, and low-added value. Here, we report a quantitative tool for achieving sustainable and low-carbon footprint planning for regional resource-based industries to facilitate sustainable and low-carbon transitions within the regional economy. To evaluate the effectiveness of this tool, the Qinghai saline lake region was chosen as a case study. After optimizing the industrial structure, the benefits of economic output, resource efficiency, and carbon emission reductions can be obtained. The scenario analyses exhibit disparities in the transition path from the original to the optimal industry structure. Notably, benefits from implementing carbon mitigation measures significantly exceeded the implementation of that of end-of-pipe measures.
Research sample	The object of the study is the selected Qinghai Salt Lake, a region rich in natural resources, such as salt lake brine, coal, natural gas, lithium resources, abundant solar and wind energy, committed to low-carbon transition and new energy development, and a typical industrial development cluster area. The object of the study is the selected Qinghai Salt Lake, a region rich in natural resources, such as salt lake brine, coal, natural gas, lithium resources, abundant solar and wind energy, committed to low-carbon transition and new energy development, and a typical industrial development cluster area. A database of more than 350 chemical industry technologies has been constructed, covering six industries and involving the processing and production of more than 250 materials and chemicals; based on the existing industrial structure of Salt Lake, industrial restructuring and optimization has been carried out, and six different scenarios have been designed to simulate the minimum transition path.
Sampling strategy	Salt lake brine supply, lithium carbonate price, lithium carbonate capacity constraints, potash capacity constraints, and various types of ore supply were selected as the parameters of specific interest for sensitivity analysis to corroborate the robustness of the model, and the parameters selected were sufficiently representative of the inputs, coefficients, and various types of parameters in the output of the model.
Data collection	The data of this study include: (1) technical process data from local research in the industrial zone; (2) data on resource types and reserves obtained from the local government in the industrial zone; (3) data on the emission factors of various types of energy sources obtained from the IPCC; and (4) process data on the technologies obtained from the literature, technical process descriptions, and other sources.
Timing and spatial scale	The data used for modeling are process data from as early as 2005 to the present, as well as local development plans and government reports of the industrial zone for the past 10 years. These data support the time scale of the article
Data exclusions	No data was excluded in this simulation analysis.
Reproducibility	Our study provides quantifiable tools for use in regional resource development and utilization, providing detailed raw data with code packages.
Randomization	Randomization

Randomization	This is not relevant to our study because our work is not an "experimental"study but an integrated data analysis. We collected enough data to represent and support various technology process, industrial structure and optimization.
Blinding	Blinding is not applicable to our study, because we collected enough data to represent and support various various technology process, industrial structure and optimization.

Did the study involve field work? ☐ Yes ☒ No

Reporting for specific materials, systems and methods

We require information from authors about some types of materials, experimental systems and methods used in many studies. Here, indicate whether each material, system or method listed is relevant to your study. If you are not sure if a list item applies to your research, read the appropriate section before selecting a response.

Materials & experimental systems

n/a	Involved in the study
☒	☐ Antibodies
☒	☐ Eukaryotic cell lines
☒	☐ Palaeontology and archaeology
☒	☐ Animals and other organisms
☒	☐ Clinical data
☒	☐ Dual use research of concern
☒	☐ Plants

Methods

n/a	Involved in the study
☒	☐ ChIP-seq
☒	☐ Flow cytometry
☒	☐ MRI-based neuroimaging

Plants

Seed stocks	Not applicable in this study.
Novel plant genotypes	Not applicable in this study.
Authentication	Not applicable in this study.
